# Design, Synthesis, and Antifouling Activity of Glucosamine-Based Isocyanides

**DOI:** 10.3390/md15070203

**Published:** 2017-06-29

**Authors:** Taiki Umezawa, Yuki Hasegawa, Ira S. Novita, Junya Suzuki, Tatsuya Morozumi, Yasuyuki Nogata, Erina Yoshimura, Fuyuhiko Matsuda

**Affiliations:** 1 Division of Environmental Materials Science, Graduate School of Environmental Science, Hokkaido University, N10W5, Sapporo 060-0810, Japan; djebelmybabumilesianpartholon@yahoo.co.jp (Y.H.); ira.novita.s@mail.ugm.ac.id (I.S.N.); suzujun55@ees.hokudai.ac.jp (J.S.); moro@ees.hokudai.ac.jp (T.M.); fmatsuda@ees.hokudai.ac.jp (F.M.); 2 Environmental Science Research Laboratory, Central Research Institute of Electric Power Industry, 1646 Abiko, Abiko, Chiba 270-1194, Japan; 3 CERES, Inc., 1-4-5 Midori, Abiko, Chiba 270-1153, Japan; megabalanusrosa@yahoo.co.jp

**Keywords:** antifouling, glucosamine, isocyanide

## Abstract

Biofouling, an undesirable accumulation of organisms on sea-immersed structures such as ship hulls and fishing nets, is a serious economic issue whose effects include oil wastage and clogged nets. Organotin compounds were utilized since the 1960s as an antifouling material; however, the use of such compounds was later banned by the International Maritime Organization (IMO) due to their high toxicity toward marine organisms, resulting in masculinization and imposex. Since the ban, there have been extensive efforts to develop environmentally benign antifoulants. Natural antifouling products obtained from marine creatures have been the subject of considerable attention due to their potent antifouling activity and low toxicity. These antifouling compounds often contain isocyano groups, which are well known to have natural antifouling properties. On the basis of our previous total synthesis of natural isocyanoterpenoids, we envisaged the installation of an isocyano functional group onto glucosamine to produce an environmentally friendly antifouling material. This paper describes an effective synthetic method for various glucosamine-based isocyanides and evaluation of their antifouling activity and toxicity against cypris larvae of the barnacle *Amphibalanus amphitrite*. Glucosamine isocyanides with an ether functionality at the anomeric position exhibited potent antifouling activity, with EC_50_ values below 1 μg/mL, without detectable toxicity even at a high concentration of 10 μg/mL. Two isocyanides had EC_50_ values of 0.23 and 0.25 μg/mL, comparable to that of CuSO_4_, which is used as a fouling inhibitor (EC_50_ = 0.27 μg/mL).

## 1. Introduction

Many essential human activities, such as transportation and fishing, are carried out in the ocean, and submarine structures—seawater intakes pipes for power plants, breeding nets, and so on—are also widely used. Ocean-based activities such as offshore energy generation will continue to increase in the future [1]. Any structure immersed in the ocean, including ship hulls and fishing nets, will encounter biofouling, that is, an undesirable accumulation of organisms such as barnacles and mussels. Biofouling can have serious economic consequences [2], including fuel waste—up to 40% [3]—and clogging of pipes and nets [4]. Removal of the fouling organisms for annual maintenance must be done at huge cost [5]. Since the 1960s, organotin compounds such as tributyltin (TBT) and triphenyltin (TPT) were widely employed to prevent fouling [6]. However, organotin compounds were suspected to be toxic, and biological consequences for marine organisms included growth inhibition, masculinization of shellfish, abnormal shell development, brittle shells, poor weight gain, and a disorder known as imposex in snails [7]. Harmful effects of TBT were also reported for marine organisms such as fish [8,9], crustaceans [10,11], and particularly mollusks [12]. These unfavorable aspects drove the International Maritime Organization (IMO) to ban the use of organotin compounds on ships worldwide in 2008 [13]. Alternative antifouling agents in current use mainly contain copper [6], and there have been some reports of negative consequences for the ocean environment and ecosystem [14,15]. The development of an environmentally friendly antifouling agent that is suitable for the ocean environment is therefore a priority.

**Figure 1:**
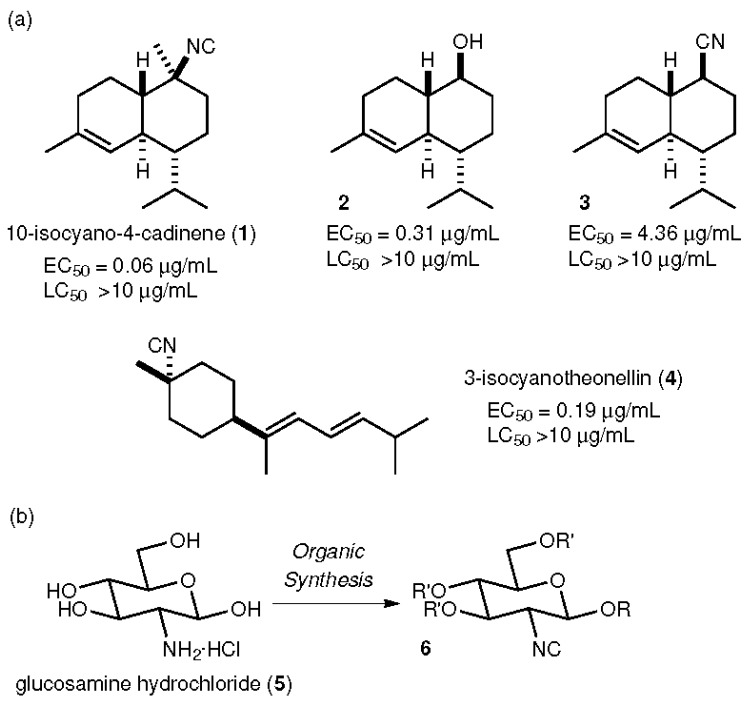
Materials containing isocyano groups exhibit potent antifouling properties. (**a**) Previously reported natural products and derivatives with antifouling properties, along with their antifouling activities. (**b**) Synthetic plan for glucosamine-based isocyanides.

In current research on green antifouling agents [16], natural products obtained from marine creatures have attracted much attention [17,18,19,20,21,22,23,24]. In particular, isocyano compounds are well-known examples of natural antifouling products. For example, 10-isocyano-4-cadinene (**1**), obtained from nudibranchs of the family *Phyllidiidae* by Okino, shows strong antifouling activity (50% effective concentration (EC_50_) = 0.14 μg/mL) against cypris larvae of the barnacle *Amphibalanus amphitrite*, comparable to CuSO_4_ (EC_50_ = 0.27 μg/mL), along with low toxicity (50% lethal concentration (LC_50_) >10 μg/mL) [25], as shown in Figure 1. Our group achieved the first total synthesis of **1** and examined the biological activity of a synthetic sample against the same cypris larvae, obtaining similar activities (EC_50_ = 0.06 μg/mL, LC_50_ > 10 μg/mL) compared to those of the natural compound [26,27,28]. Alcohol **2** and the nitrile **3**, synthetic intermediates in the total synthesis of **1**, were found to have less potency than **1**. Other isocyanide compounds such as 3-isocyanotheonellin (**4**) also exhibited potent antifouling profiles [29,30,31,32,33]. These results clearly indicate that the isocyano group confers potent antifouling properties. We envisaged a compound in which the isocyano functionality was installed on a glucosamine unit (**5**), obtained from crustaceans such as crabs and shrimp as a cheap and abundant biomass platform, through organic synthesis, because **5** possesses an amino group, which is essential for introduction of the isocyano group. In addition, the four hydroxy groups in **5** enable easy access to a wide variety of synthetic isocyanides. Furthermore, the compound, which is derived from a sugar-based structure, was expected to have low toxicity. In this study, glucosamine-based isocyanides (**6**) were designed and synthesized in short reaction sequences. The antifouling activities and toxicities of the synthetic compounds **6** against the cypris larvae of *Amphibalanus amphitrite* were evaluated to prove the feasibility of these compounds as alternative environmentally friendly antifouling agents, including through investigation of substituent effects at the C-1 and C-3, 4, and 6 positions.

## 2. Results

### 2.1. Compounds with Ester Groups at the C-1 Position

**Figure 2:**
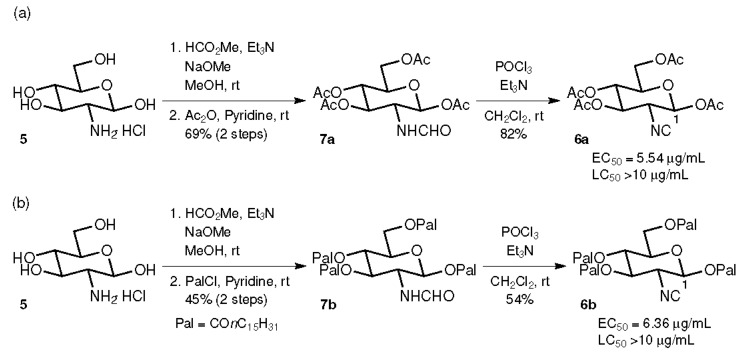
Synthesis and biological activity of tetraacylated glucosamine-based isocyanides. (**a**) Synthesis of tetraacetate **6a**. (**b**) Synthesis of tetrapalmitate **6b**.

Initially, the tetraacetate **6a** and the tetrapalmitate **6b** were prepared from d-glucosamine hydrochloride (**5**) by a sequence of formamide formation with HCO_2_Me and NaOMe [34], the acylation of hydroxy groups with Ac_2_O or palmitoyl chloride (PalCl), respectively, and the dehydration of formamides **7a** and **7b** with POCl_3_ and Et_3_N [35] (Figure 2). The antifouling activities of **6a** and **6b** were evaluated against the cypris larvae of *Amphibalanus amphitrite* after a 48 h incubation period, showing moderate activity (EC_50_ = 5.54 and 6.36 μg/mL, respectively) and very low toxicity (lethal larva was not observed at the concentration of 10 μg/mL).

### 2.2. Compounds with Ether at the C-1 Position

**Figure 3:**
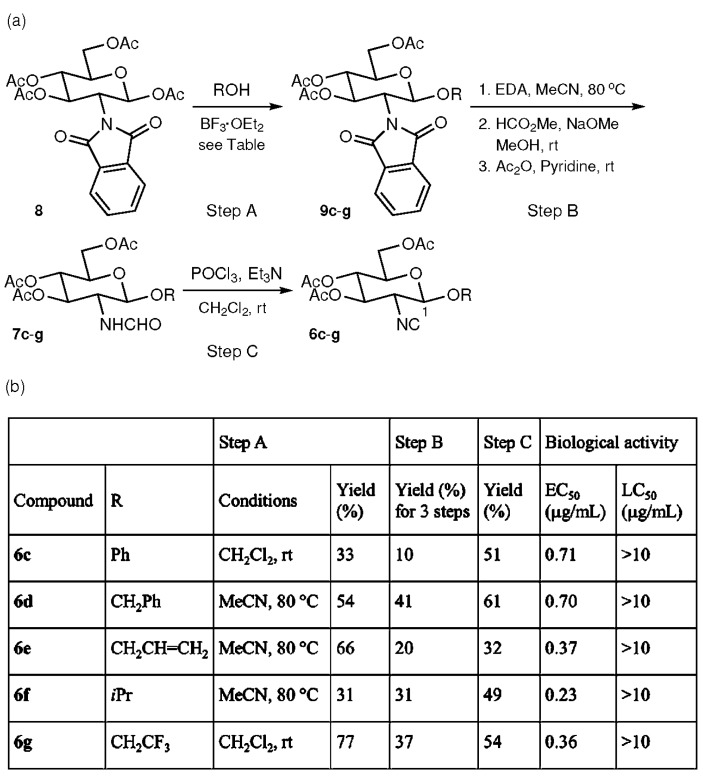
Synthesis and biological activities of glucosamine-based isocyanides. (**a**) Synthesis of isocyanides **6c**–**g**. (**b**) Table of reactions and biological activities.

Next, the effect of replacement of the C-1 ester groups of **6a** and **6b** with ether groups upon antifouling activity and toxicity against *Amphibalanus amphitrite* was examined. Phenyl (Ph), benzyl (CH_2_Ph), allyl (CH_2_CH = CH_2_), isopropyl (CH(CH_3_)_2_, *i*Pr), and 2,2,2-trifluoroethyl (CH_2_CF_3_) groups were chosen as the substituent R of the C-1 alkoxy group. Figure 3 summarizes the synthesis and biological activities of isocyanides **6c**–**g**. For the synthesis of **6c**–**g**, the commercially available d-glucosamine derivative **8** was employed as a starting compound because glycosidation reactions between formamide **7a** and alcohols such as isopropyl alcohol under various conditions did not afford the desired glycosidation products. In glycosidation reactions of **8** promoted by BF_3_·OEt_2_ (Step A), two solvent systems (CH_2_Cl_2_/room temperature, and MeCN/80 °C) were employed for optimization of chemical yield. Phenyl glycoside **9c** and trifluoroethyl glycoside **9g** were obtained in higher yields by allowing **8** to react with phenol and 2,2,2-trifluoroethanol at room temperature in CH_2_Cl_2_. However, treatment of **8** with benzyl alcohol, allyl alcohol, and isopropyl alcohol at 80 °C in MeCN produced benzyl glycoside **9d**, allyl glycoside **9e**, and isopropyl glycoside **9f** in better yields. During each of the glycosidation reactions, the *β*-anomer was formed as the sole glycosidation product. The *β*-configurations at the anomeric position (C-1) of **9c**–**g** were determined based on coupling constant analysis. Thus, a large vicinal coupling constant, *J*_12_ = 9 Hz (axial/axial relationship between the vicinal methine protons at C-1 and 2), was observed in all the ^1^H NMR spectra. The conversion of **9c**–**g** into formamides **7c**–**g** was accomplished in the following three steps (Step B): (i) removal of the phthaloyl and acetyl groups with ethylenediamine (EDA); (ii) a formamide formation reaction with HCO_2_Me and NaOMe; and (iii) acetylation of the three resulting hydroxyl groups with Ac_2_O and pyridine. Isocyanides **6c**–**g** were derived from **7c**–**g** using the same dehydration protocol with POCl_3_ and Et_3_N (Step C) that was used in the preparation of **7a** and **7b**. The biological activities of synthetic **6c**–**g** against the cypris larvae of *Amphibalanus amphitrite* were then evaluated to reveal enhanced potency for **6c**–**g** (EC_50_ = 0.23–0.71 μg/mL) relative to **6a** and **6b** (EC_50_ = 5.54 and 6.36 μg/mL, respectively). Furthermore, it was noteworthy that mortality among the cypris larvae was not observed during the assay, even at a high concentration of 10 μg/mL.

### 2.3. Effects of Substituents at the C-3, 4, and 6 Positions

**Figure 4:**
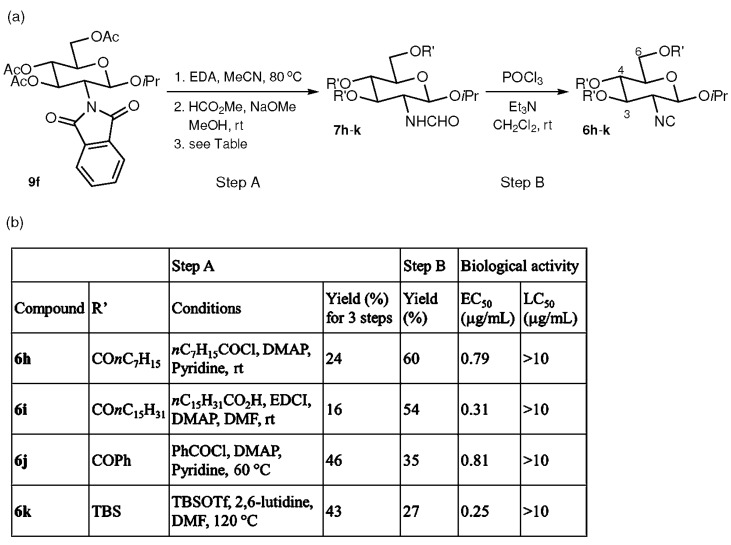
Synthesis and biological activity of glycosylated glucosamine-based isocyanides. (**a**) Synthesis of isocyanides **6h**–**k**. (**b**) Table of reactions and biological activities.

Further modifications were made by replacing the acetyl groups at C-3, 4, and 6 of the isopropyl glycoside **6f** with other substituents. As illustrated in Figure 4, after removal of the phthaloyl and acetyl groups of **9f** followed by formamide formation, octanoyl (CO*n*C_7_H_15_), palmitoyl (CO*n*C_15_H_31_), benzoyl (COPh), and TBS (SiMe_2_*t*Bu) groups were installed to afford isopropyl glycosides **7h**–**k**, respectively. Each of **7h**–**k** was transformed to the corresponding isocyanide (**6h**–**k**) under dehydration conditions that were the same as those used for the preparation of **6a**–**g**. Isocyanides **6h**–**k** were tested against *Amphibalanus amphitrite* and exhibited similar antifouling activities (EC_50_ = 0.25–0.81 μg/mL) to **6f** (EC_50_ = 0.23 μg/mL). Again, none of **6h**–**k** showed detectable toxicity (LC_50_ > 10 μg/mL).

## 3. Discussion

These procedures are short syntheses (up to 5 steps from commercially available materials) allowing access to various glucosamine-based isocyanides using inexpensive reagents. In contrast, 27 steps were required for the total synthesis of **1**. Although the chemical yields should be improved by further optimization, the synthetic scheme seems to be a reliable one for the synthesis of various isocyanides with latent antifouling activity. All of the samples obtained in the current study showed not only antifouling profiles but also extremely low toxicity against the cypris larvae of *Amphibalanus amphitrite*. Compounds **6a** and **6b** with the ester group at the C-1 position had EC_50_ values above 5 μg/mL, but **6c**–**g** with the ether group at the C-1 position were more active, with EC_50_ values below 1 μg/mL. Compounds **6f** and **6k** exhibited EC_50_ values of 0.23 and 0.25 μg/mL, comparable to that of CuSO_4_, which is used as a fouling inhibitor (EC_50_ = 0.27 μg/mL) [27]. Substituent effects at the C-1 position of glucosamine were clearly evident in the higher EC_50_ values of **6a** and **6b** compared to **6c**–**g**, suggesting that more detailed investigations of ether substituents at this position may enable the discovery of more potent compounds, and that substituents at the C-1 position affect the antifouling mechanism through steric or electronic effects [36,37] (Figure 2 and Figure 3). On the other hand, no significant effect on antifouling activity was obtained by changing the substituents attached to the three hydroxy groups at C-3, 4, and 6 (Figure 4). This result is advantageous from the point of view of the development of nontoxic and environmentally benign antifouling agents through the synthesis of functionalized glucosamine derivatives—for example, monomers for the synthesis of polymers that can be used in combination with paint—since it is possible to introduce various functional groups at the C-3, 4, and 6 positions without significant loss of antifouling activity.

The results obtained in this study are promising in terms of the development of green antifouling agents for practical use. Further preparation of a wide variety of glucosamine-based isocyanides along with relevant field assay and biodegradability test will be reported in our laboratory.

## 4. Materials and Methods

Optical rotations were obtained using a Horiba SEPA-300 instrument (Horiba, Kyoto, Japan). IR spectra were recorded on a JASCO FT/IR 4100 spectrometer using an NaCl cell. ^1^H and ^13^C NMR spectra were recorded using a JNM-EX 400 (400 MHz and 100 MHz) spectrometer (see Supplementary Materials). *N*,*N*-dimethylformamide (DMF), methanol (MeOH), and acetonitrile (MeCN) were purchased from Kanto Chemical Co. Inc. (Tokyo, Japan). Dichloromethane (CH_2_Cl_2_) was distilled from CaH_2_. All commercially available reagents were employed as received. Analytical TLC was carried out using pre-coated silica gel plates (TLC silica gel 60 F_254_). The silica gel used for column chromatography was Wakogel 60N 63–212 μm.

### 4.1. General Procedure for the Preparation of Formamides ***7a*** and ***7b***

To a solution of d-glucosamine hydrochloride (**5**) (1.0 equiv) in MeOH (0.40 M) and HCO_2_Me (1.0 M), NaOMe (1.4 equiv) and Et_3_N (1.0 equiv) was added at room temperature under an Ar atmosphere. The mixture was stirred at room temperature overnight and then concentrated in vacuo. To a solution of the crude formamide in pyridine (15 equiv), Ac_2_O (6.0 equiv) for **7a** or palmitoyl chloride (6.0 equiv) for **7b** was added at room temperature under an Ar atmosphere. After stirring at room temperature overnight, MeOH (6.0 equiv) was added at 0 °C. The mixture was extracted with EtOAc, washed successively with 1.0 M NaOH, 3.0 M HCl, and brine, dried over Na_2_SO_4_, filtered, and concentrated in vacuo. The residue was purified by silica gel column chromatography (EtOAc/hexane = 40:60) to yield **7a** or **7b**, respectively.

### 4.2. General Procedure for the Preparation of Isocyanides ***6a***–***k***

To a solution of each of the series of formamides **7a**–**k** (1.0 equiv) in CH_2_Cl_2_ (0.20 M), Et_3_N (9.0 equiv) and POCl_3_ (3.0 equiv) were added dropwise at 0 °C under an Ar atmosphere. After stirring at 0 °C for 10 min, the mixture was warmed to room temperature, stirred at room temperature for 1 h, quenched with saturated NaHCO_3_, and extracted with EtOAc. The combined organic extracts were washed with brine, dried over Na_2_SO_4_, filtered, and concentrated in vacuo. The residue was purified by silica gel column chromatography (EtOAc/hexane = 10:90) to yield isocyanides **6a**–**k**, respectively.

### 4.3. General Procedure for the Preparation of Glycosides ***9d***–***f***

To a solution of **8** (1.0 equiv) in MeCN (0.25 M), benzyl alcohol, allyl alcohol, or isopropyl alcohol (1.0 equiv) and BF_3_·OEt_2_ (3.0 equiv) were added at room temperature under an Ar atmosphere. The mixture was stirred at 80 °C overnight, quenched with saturated aqueous NaHCO_3_, and extracted with EtOAc. The combined organic extracts were washed with brine, dried over Na_2_SO_4_, filtered, and concentrated in vacuo. The residue was then purified by silica gel column chromatography (EtOAc/hexane = 20:80) to yield **9d**–**f**, respectively.

### 4.4. General Procedure for the Preparation of Glycosides ***9c*** and ***9g***

To a solution of **8** (1.0 equiv) in CH_2_Cl_2_ (0.20 M), phenol or 2,2,2-trifluoroethanol (2.0 equiv) and BF_3_·OEt_2_ (2.0 equiv) were added at room temperature under an Ar atmosphere. The mixture was stirred at room temperature overnight, quenched with saturated aqueous NaHCO_3_, and extracted with AcOEt. The combined extracts were washed with brine, dried over Na_2_SO_4_, filtered, and concentrated in vacuo. The residue was then purified by silica gel column chromatography (EtOAc/hexane = 30:70) to yield **9c** or **9g**, respectively.

### 4.5. General Procedure for the Preparation of Formamides ***7c***–***g*** from Glycosides ***9c**–**g***

To a solution of each of **9c**–**g** (1.0 equiv) in MeCN (0.20 M), EDA (4.7 equiv) was added at room temperature under an Ar atmosphere. The mixture was stirred at 80 °C overnight and concentrated in vacuo. To a solution of the crude amine in MeOH (0.40 M) and HCO_2_Me (0.40 M), NaOMe (1.2 equiv) was added at room temperature under an Ar atmosphere. After stirring at room temperature overnight, DOWEX was added for neutralization. The mixture was filtered through a celite pad and concentrated in vacuo. To a solution of the crude formamide in pyridine (30 equiv), Ac_2_O (25 equiv) was added at room temperature under an Ar atmosphere. After stirring at room temperature overnight, MeOH (25 equiv) was added at 0 °C. The mixture was extracted with EtOAc, washed successively with 1.0 M NaOH (25 equiv), 3.0 M HCl, and brine, dried over Na_2_SO_4_, filtered, and concentrated in vacuo. The residue was purified by silica gel column chromatography (EtOAc/hexane = 40:60) to yield **7c**–**g**, respectively.

### 4.6. Synthesis of ***7h***

To a solution of crude formamide in pyridine (50 equiv) and DMAP (0.50 equiv), obtained from **9f** in two steps, octanoyl chloride (6.0 equiv) was added at room temperature under an Ar atmosphere. The mixture was stirred for 24 h, quenched with 3.0 M HCl (60 equiv), diluted with AcOEt, washed with brine, dried over Na_2_SO_4_, filtered and concentrated in vacuo. The residue was purified by silica gel column chromatography (EtOAc:Hexane = 5:95) to yield **7h** (24% over three steps) as a white solid.

### 4.7. Synthesis of ***7i***

To a solution of crude formamide obtained from **9f** in two steps, DMF (0.050 M), palmitic acid (5.0 equiv), DMAP (0.50 equiv), and EDCI (5.0 equiv) were added at room temperature under an Ar atmosphere. The mixture was stirred for 20 h, supplemented with EtOH (2.0 equiv) and EDCI (2.0 equiv), quenched with saturated aqueous NH_4_Cl, diluted with AcOEt, washed with brine, dried over Na_2_SO_4_, filtered, and concentrated in vacuo. The residue was purified by silica gel column chromatography (EtOAc:hexane = 10:90) to yield **7i** (16% over three steps) as a white solid.

### 4.8. Synthesis of ***7j***

To the solution of crude formamide in pyridine (15 equiv) and DMAP (0.50 equiv), obtained from **9f** in two steps, benzoyl chloride (5.0 equiv) was added at 60 °C under an Ar atmosphere. The mixture was stirred for 24 h, quenched with 3.0 M HCl (60 equiv), diluted with AcOEt, washed with brine, dried over Na_2_SO_4_, filtered and concentrated in vacuo. The residue was purified by silica gel column chromatography (EtOAc:hexane = 30:70) to yield **7j** (46% over three steps) as a white solid.

### 4.9. Synthesis of ***7k***

To the solution of crude formamide, obtained from **9f** in two steps, in DMF (0.050 M) and 2,6-Lutidine (15 equiv), TBSOTf (20 equiv) was added at 120 °C under an Ar atmosphere. The mixture was stirred for 1.5 h, quenched with saturated aqueous NaHCO_3_, diluted with AcOEt, washed with brine, dried over Na_2_SO_4_, filtered, and concentrated in vacuo. The residue was purified by silica gel column chromatography (EtOAc:hexane = 10:90) to yield **7k** (43% over three steps) as a white solid.

### 4.10. Antifouling Assay

Adult barnacles (*Amphibalanus amphitrite*) procured from oyster farms at Lake Hamana and a pier at Shimizu bay, Shizuoka, were kept in an aquarium at 20 °C and fed on *Artemia salina* nauplii. Broods were released as I–II stage nauplii upon immersion in seawater after drying overnight. The nauplii thus obtained were cultured in filtered natural seawater (salinity 28) containing penicillin G (20 μg/mL) and streptomycin sulfate (30 μg/mL) and were fed on the diatom *Chaetoceros gracilis* at concentrations of 40 × 10^4^ cells/mL. Larvae reached the cyprid stage in 5 days. The cyprids were collected and stored at 4 °C until use (0 days old).

The test compounds were dissolved in ethanol and aliquots of the solution were transferred to wells of a 24-well polystyrene culture plate and air-dried. Four wells were used for each concentration. To each well, filtered seawater (2.0 mL, salinity 28) and six 2-day-old cyprids were added. The plates were kept in the dark at 25 °C for 48 h. The numbers of cyprids that attached, metamorphosed, died, and did not settle were counted under a microscope. Three or four trials were done for each concentration. Probit analysis was used to calculate the EC_50_ values.

## Supplementary Materials

The following are available online at www.mdpi.com/1660-3397/15/7/203/s1; characterization of all compounds and 1H and 13C NMR data are available.
