# Supplementary material for: Design, Synthesis, and Antifouling Activity of Glucosamine-Based Isocyanides
Source: Mar Drugs. 2017 Jun 29;15(7):203. doi: 10.3390/md15070203 (PMC5532645; doi:10.3390/md15070203)
Supplement: Supplementary file 1 [file marinedrugs-15-00203-s001.pdf]

Design, Synthesis, and Antifouling Activity of Glucosamine-based Isocyanides

Taiki Umezawa[1,*], Yuki Hasegawa[1], Ira S. Novita[1], Junya Suzuki[1], Tatsuya Morozumi[1], Yasuyuki Nogata[2*], Erina Yoshimura[3], Fuyuhiko Matsuda[1]

[1]Division of Environmental Materials Science, Graduate School of Environmental Science, Hokkaido University, N10W5 Sapporo 060-0810, Japan. [2]Environmental Science Research Laboratory, Central Research Institute of Electric Power Industry, 1646 Abiko, Abiko, Chiba 270-1194, Japan. [3]CERES, Inc., 1-4-5 Midori, Abiko, Chiba 270-1153, Japan.

*T.U. (email: umezawa@ees.hokudai.ac.jp),

* Y.N. (email: noga@criepi.or.jp)

7a. **7a** was synthesized according to the general procedure in the main text (69% yield). $[\alpha]_D^{26}$ +65.3 (*c* 7.51, $CHCl_3$); IR (neat) 3327, 3024, 2959, 1747, 1692, 1529, 1432, 1370, 1220, 1145, 1031, 937, 755, 667 cm^{-1}; ^1H NMR (400 MHz, $CDCl_3$) δ 2.03 (3H, s), 2.05 (3H, s), 2.08 (3H, s), 2.18 (3H, s), 4.00 (1H, ddd, J = 2.4, 3.9, 9.8 Hz), 4.06 (1H, dd, J = 1.8, 12.6 Hz), 4.25 (1H, dd, J = 3.9, 12.2 Hz), 4.55 (1H, td, J = 2.9, 9.7 Hz), 5.18 – 5.31 (2H, m), 5.73 - 5.31 (1H, m), 6.20 (1H, t, J = 3.9 Hz), 8.13 (1H, s); ^{13}C NMR (100 MHz, $CDCl_3$) δ 20.4, 20.9, 21.0, 21.2, 49.8, 61.7, 67.7, 70.4, 70.7, 90.6, 160.9, 168.8, 169.4, 171.0, 171.9; HRMS (ESI) m/z: $[M + Na]^+$ Calcd for $C_{15}H_{21}O_{10}NNa$, 398.1057; Found 398.1050.

6a. **6a** was synthesized according to the general procedure in the main text (82% yield). $[\alpha]_D^{16}$ +145.5 (*c* 2.01, $CHCl_3$); IR (neat) 3024, 2963, 2854, 2148, 1757, 1432, 1371, 1214, 1144, 1090, 1014, 973, 937, 889, 756, 668 cm^{-1}; ^1H NMR (400 MHz, $CDCl_3$) δ 2.04 (3H, s), 2.07 (3H, s), 2.11 (3H, s), 2.24 (3H, s), 3.98 (1H, dd, J = 3.9, 10.7 Hz), 4.03 (1H, dd, J = 2.4, 12.2 Hz), 4.07 – 4.11 (1H, m), 4.28 (1H, dd, J = 3.9, 12.2 Hz), 5.01 (1H, t, J = 9.7 Hz), 5.54 (1H, t, J = 10.7 Hz), 6.40 (1H, t, J = 3.4 Hz); ^{13}C NMR (100 MHz, $CDCl_3$) δ 20.5, 20.6, 54.7, 61.1, 67.1, 69.7, 70.0, 88.3, 168.3, 169.3, 169.7, 170.4; HRMS (ESI) m/z: $[M + Na]^+$ Calcd for $C_{15}H_{19}O_9NNa$, 380.0958; Found 380.0951.

7b. **7b** was synthesized according to the general procedure in the main text (45% yield). $[\alpha]_D^{26}$ +24.5 (*c* 1.02, $CHCl_3$); IR (neat) 2955, 2918, 2850, 1743, 1684, 1520, 1467, 1377, 1243, 1220, 1166, 1113, 1029, 931, 758, 721 cm^{-1}; ^1H NMR (400 MHz, $CDCl_3$) δ 0.87 (12H, t, J = 6.8 Hz), 1.25 – 1.71 (104H, m), 2.23 – 2.55 (8H, m), 3.99 - 4.33 (4H, m), 5.15 – 5.33 (2H, m), 5.63 - 5.70 (1H, m), 6.21 (1H, t, J = 3.4 Hz), 8.11 (1H, s); ^{13}C NMR (100 MHz, $CDCl_3$) δ14.1, 22.7, 24.7, 24.8, 29.09, 29.12, 29.23, 29.36, 29.44, 29.48, 29.51, 29.62, 29.66, 29.70, 31.9, 49.7, 61.1, 61.3, 61.5, 61.7, 62.6, 63.6, 65.8, 66.8, 67.0, 69.9, 90.0, 90.1, 95.3, 119.9, 158.7, 159.8, 171.31, 171.35, 172.4, 173.4, 174.6 .

6b. **6b** was synthesized according to the general procedure in the main text (54% yield). $[\alpha]_D^{16}$ +59.3 (*c* 0.91, $CHCl_3$); IR (neat) 2955, 2917, 2850, 2150, 1754, 1467, 1379, 1265, 1243, 1220, 1168, 1108, 1032, 924, 721, cm^{-1}; ^1H NMR (400 MHz, $CDCl_3$) δ 0.88 (12H, t, J = 6.8 Hz), 1.25 – 1.71 (104H, m), 2.23 – 2.55 (8H, m), 3.96 (1H, dd, J = 3.4, 10.7 Hz), 4.03 – 4.09 (2H, m), 4.22 (1H, dd, J = 4.4, 10.7 Hz), 5.03 (1H, t, J = 9.7 Hz), 5.56 (1H, t, J = 10.2 Hz), 6.41 (1H, t, J = 3.9 Hz); ^{13}C NMR (100 MHz, $CDCl_3$) δ 14.1, 22.7, 24.68, 24.71, 29.0, 29.1, 29.21, 29.25, 29.3, 29.45, 29.49, 29.61, 29.66, 29.7, 31.9, 33.88, 33.91, 33.94, 54.9, 61.0, 66.8, 69.6, 70.1, 88.1, 171.2, 172.0, 172.4, 173.2.

9c. 9c was synthesized according to the general procedure in the main text (33% yield). $[\alpha]_D^{27}$ +57.9 (*c* 4.52, $CHCl_3$); IR (neat) 3481, 3027, 2947, 1747, 1718, 1590, 1494, 1468, 1429, 1384, 1337, 1220, 1173, 1082, 1040, 973, 900, 873, 818, 794, 756, 725, 691, 667, 639 cm^{-1}; ^1H NMR (400 MHz, $CDCl_3$) δ 1.88 (3H, s), 2.04 (3H, s), 2.11 (3H, s), 4.01 (1H, ddd, *J* = 2.4, 4.9, 10.2 Hz), 4.17 (1H, dd, *J* = 2.4, 12.2 Hz), 4.35 (1H, dd, *J* = 5.4, 10.7 Hz), 4.60 (1H, dd, *J* = 8.3, 10.7 Hz), 5.22 (1H, dd, *J* = 9.3, 10.2 Hz), 5.88 (1H, dd, *J* = 9.3, 10.7 Hz), 5.98 (1H, d, *J* = 8.3 Hz), 6.90 (1H, dd, *J* = 1.0, 8.9 Hz), 6.99 (1H, t, *J* = 7.8 Hz), 7.20 (2H, dd, *J* = 7.3, 7.8 Hz), 7,72 (2H, t, *J* = 3.4, 5.3 Hz), 7.84 (1H, t, *J* = 2.9, 5.3 Hz); ^{13}C NMR (100 MHz, $CDCl_3$) δ 20.4, 20.5, 20.6, 54.4, 61.9, 68.9, 70.6, 72.0, 96.2, 117.0, 123.2, 123.6, 129.4, 131.2, 134.3, 156.3, 169.4, 170.0, 170.6; HRMS (ESI) m/z: $[M + Na]^+$ Calcd for $C_{26}H_{25}O_{10}NNa$, 534.1370; Found 534.1365.

7c. 7c was synthesized according to the general procedure in the main text (10% overall yield). $[\alpha]_D^{25}$ +12.9 (*c* 0.28, $CHCl_3$); IR (neat) 3749, 3734, 3674, 3648, 3336, 2958, 2883, 2374, 2348, 2310, 1748, 1671, 1590, 1520, 1374, 1224, 1070, 1043, 903, 758, 692 cm^{-1}; ^1H NMR (400 MHz, $CDCl_3$) δ 2.03 (3H, s), 2.05 (3H, s), 2.06 (3H, s), 3.88 (1H, ddd, *J* = 2.4, 5.3, 7.8 Hz), 4.13 – 4.23 (1H, m), 4.29 (1H, dd, *J* = 5.3, 12.2 Hz), 4.89 (1H, d, *J* = 8.3 Hz), 5.10 – 5.18 (1H, m), 5.26 (1H, d, *J* = 8.3 Hz), 5.44 (1H, dd, *J* = 9.3, 10.7 Hz), 5.75 – 5.81 (1H, m), 6.97 – 7.13 (3H, m), 7.28 (2H, dd, *J* = 1.4, 8.4 Hz), 8.20 (1H, s); ^{13}C NMR (100 MHz, $CDCl_3$) δ 20.5, 20.6, 20.7, 53.6, 56.8, 61.9, 62.0, 68.0, 68.5, 71.5, 71.8, 71.9, 72.0, 98.8, 99.8, 116.8, 117.0, 123.2, 123.7, 129.5, 129.7, 156.6, 156.8, 161.0, 164.8, 169.4, 170.5, 170.6, 170.8; HRMS (ESI) m/z: $[M + Na]^+$ Calcd for $C_{19}H_{23}O_9NNa$, 432.1265; Found 432.1260.

6c. 6c was synthesized according to the general procedure in the main text (51% yield). $[\alpha]_D^{25}$ +8.0 (*c* 0.13, $CHCl_3$); IR (neat) 2959, 2361, 2342, 2147, 1757, 1591, 1493, 1368, 1221, 1051, 898, 817, 757, 692 cm^{-1}; ^1H NMR (400 MHz, $CDCl_3$) δ 2.04 (3H, s), 2.08 (3H, s), 2.14 (3H, s), 3.86 (1H, ddd, *J* = 2.4, 5.3, 7.3 Hz), 3.94 (1H, dd, *J* = 7.8, 10.2 Hz), 4.15 (1H, dd, *J* = 2.4, 12.7 Hz), 4.31 (1H, dd, *J* = 5.3, 12.2 Hz), 5.02 (1H, t, *J* = 9.8 Hz), 5.09 (1H, d, *J* = 8.3 Hz), 5.39 (1H, dd, *J* = 9.3, 10.2 Hz), 7.09 (2H, dd, *J* = 1.0, 8.8 Hz), 7.13 (1H, t, *J* = 7.3 Hz), 7.35 (2H, t, *J* = 7.3 Hz); ^{13}C NMR (100 MHz, $CDCl_3$) δ 20.7, 20.8, 20.9, 57.4, 61.9, 67.8, 72.3, 72.5, 99.5, 117.9, 124.5, 130.0, 156.7,169.7, 169.9, 170.8; HRMS (ESI) m/z: $[M + Na]^+$ Calcd for $C_{19}H_{21}O_8NNa$, 414.1159; Found 414.1168.

9d. 9d was synthesized according to the general procedure in the main text (54% yield). $[\alpha]_D^{28}$ +7.9 (*c* 0.32, $CHCl_3$); IR (neat) 2928, 2342, 1752, 1718, 1379, 1369, 1228, 1139, 1079, 1040, 975, 899, 722, 701 cm^{-1}; ^1H NMR (400 MHz, $CDCl_3$) δ 1.86 (3H, s), 2.02 (3H, s), 2.13 (3H, s), 3.86 (1H, ddd, *J* = 2.4, 4.4, 10.2 Hz), 4.20 (1H, dd, *J* = 2.0, 12.4 Hz), 4.32 – 4.37 (1H, m), 4.38

(1H, t, J = 10.7 Hz), 4.53 (1H, d, J = 12.2 Hz), 4.85 (1H, d, J = 11.7 Hz), 5.19 (1H, t, J = 9.8 Hz), 5.37 (1H, d, J = 8.3 Hz), 5.78 (1H, dd, J = 9.3, 10.7 Hz), 7.07 – 7.16 (5H, m), 7.71 – 7.79 (4H, m); ^{13}C NMR (100 MHz, $CDCl_3$) δ 20.7, 20.9, 21.1, 54.9, 62.3, 69.3, 70.9, 72.1, 97.4, 123.8, 128.0, 128.1, 128.5, 134.4, 136.2, 169.8, 170.4, 171.0; HRMS (ESI) m/z: $[M + Na]^+$ Calcd for $C_{27}H_{27}O_{10}NNa$, 548.1527; Found 548.1519.

7d. 7d was synthesized according to the general procedure in the main text (41% overall yield). $[\alpha]_D^{25}$ –31.6 (c 1.57, $CHCl_3$); IR (neat) 2928, 2342, 2342, 1752, 1718, 1379, 1369, 1228, 1139, 1079, 1040, 975, 899, 722, 701 cm^{-1}; ^{1}H NMR (400 MHz, $CDCl_3$) δ 2.02 (3H, s), 2.10 (3H, s), 2.16 (3H, s), 3.61 – 3.71 (1H, m), 4.03 (1H, dt, J = 2.9, 12.2 Hz), 4.24 – 4.37 (2H, m), 4.58 – 4.72 (2H, m), 4.90 (1H, d, J = 11.7 Hz), 5.09 (1H, td, J = 2.4, 9.8 Hz), 5.27 (1H, t, J = 9.3 Hz), 5.46 – 5.49 (1H, m), 7.36 – 7.28 (5H, m), 8.13 (1H, s); ^{13}C NMR (100 MHz, $CDCl_3$) δ 20.8, 20.9, 21.0, 21.1, 21.1, 31.2, 53.0, 57.2, 62.2, 62.3, 68.4, 68.8, 71.0, 71.5, 72.1, 72.2, 72.2, 72.4, 99.4, 99.8, 128.4, 128.4, 128.5, 128.7, 128.8, 128.9, 136.8, 161.2, 165.1, 169.7, 170.9, 171.0, 171.0, 170.2; HRMS (ESI) m/z: $[M + Na]^+$ Calcd for $C_{27}H_{27}O_{10}NNa$, 446.1421; Found 446.1415.

6d. 6d was synthesized according to the general procedure in the main text (61% yield). $[\alpha]_D^{25}$ +4.5 (c 0.09, $CHCl_3$); IR (neat) 3035, 2952, 2881, 2360, 2147, 1754, 1497, 1455, 1367, 1226, 1164, 1109, 1054, 969, 898, 752, 700cm^{-1}; ^{1}H NMR (400 MHz, $CDCl_3$) δ 2.03 (3H, s), 2.10 (3H, s), 2.10 (3H, s), 3.68 (1H, ddd, J = 2.2, 4.4, 10.7 Hz), 3.71 (1H, dd, J = 7.8, 10.7 Hz), 4.14 (1H, dd, J = 2.0, 12.8 Hz), 4.28 (1H, dd, J = 4.4, 12.8 Hz), 4.60 (1H, d, J = 8.3 Hz), 4.70 (1H, d, J = 11.7 Hz), 4.95 (1H, d, J = 11.7 Hz), 4.96 (1H, d, J = 8.8 Hz), 5.27 (1H, t, J = 9.3 Hz), 7.32 – 7.44 (5H, m); ^{13}C NMR (100 MHz, $CDCl_3$) δ 20.7, 20.8, 21.0, 54.3, 54.4, 61.9, 68.0, 71.7, 72.3, 72.4, 98.8, 128.6, 128.8, 129.0, 135.9, 169.7, 169.9, 170.8; HRMS (ESI) m/z: $[M + Na]^+$ Calcd for $C_{20}H_{23}O_8NNa$, 428.1216; Found 428.1324.

9e. 9e was synthesized according to the general procedure in the main text (66% yield). $[\alpha]_D^{27}$ +42.1 (c 1.04, $CHCl_3$); IR (neat) 3480, 3022, 2925, 1747, 1716, 1468, 1428, 1384, 1225, 1128, 1079, 1038, 899, 772, 725 cm^{-1}; ^{1}H NMR (400 MHz, $CDCl_3$) δ 1.80 (3H, s), 1.97 (3H, s), 2.06 (3H, s), 3.80 (1H, ddd, J = 2.4, 4.9, 10.2 Hz), 4.00 (1H, dd, J = 6.8, 13.2 Hz), 4.12 (1H, dd, J = 2.0, 12.2 Hz), 4.20 – 4.34 (3H, m), 5.04 (2H, ddd, J = 1.0, 10.2, 14.6 Hz), 5.12 (1H, t, J = 10.2 Hz), 5.35 (1H, d, J = 8.3 Hz), 5.62 – 5.71 (1H, m), 5.73 (1H, dd, J = 9.3, 10.7 Hz), 5.73 (1H, dd, J = 9.3, 10.7 Hz), 7.68 (2H, dd, J = 2.9, 5.4 Hz), 7.80 (2H, dd, J = 2.9, 5.4 Hz); ^{13}C NMR (100 MHz, $CDCl_3$) δ 20.8, 20.9, 21.0, 54.9, 62.3, 69.3, 70.5, 71.0, 72.1, 97.4, 118.2, 123.9, 131.7, 133.5, 134.6, 169.8, 170.5, 171.1; HRMS (ESI) m/z: $[M + Na]^+$ Calcd for $C_{23}H_{25}O_{10}NNa$, 498.1370; Found 498.1366.

7e. 7e was synthesized according to the general procedure in the main text (20% overall yield). $[\alpha]_D^{26}$ +26.0 (*c* 0.41, $CHCl_3$); IR (neat) 3084, 2941, 2881, 2148, 1752, 1366, 1223, 1170, 1110, 1053, 929, 771 cm^{-1}; ^1H NMR (400 MHz, $CDCl_3$) δ 2.02 (3H, s), 2.08 (3H, s), 2.11 (3H, s), 3.67 (1H, dd, *J* = 8.3, 10.2 Hz), 3.67 – 3.75 (1H, m), 4.17 (1H, dd, *J* = 2.4, 12.2 Hz), 4.20 (1H, dd, *J* = 6.8, 12.6 Hz), 4.27 (1H, dd, *J* = 4.4, 12.2 Hz), 4.62 (1H, d, *J* = 9.8 Hz), 4.95 (1H, d, *J* = 7.8 Hz), 5.41 – 5.26 (3H, m), 5.92 (1H, ddd, *J* = 5.8, 11.2, 17.0 Hz); ^{13}C NMR (100 MHz, $CDCl_3$) δ 20.1, 20.2, 20.4, 29.4, 57.0, 61.2, 67.3, 67.4, 70.4, 70.5, 71.7, 71.8, 98.5, 98.6,118.6, 132.3, 169.1, 169.3, 170.2; HRMS (ESI) m/z: $[M + Na]^+$ Calcd for $C_{16}H_{21}O_8NNa$, 378.1159; Found 378.1166.

6e. 6e was synthesized according to the general procedure in the main text (32% yield). $[\alpha]_D^{26}$ +26.0 (*c* 0.41, $CHCl_3$); IR (neat) 3084, 2941, 2881, 2148, 1752, 1366, 1223, 1170, 1110, 1053, 929, 771 cm^{-1}; ^1H NMR (400 MHz, $CDCl_3$) δ 2.02 (3H, s), 2.08 (3H, s), 2.11 (3H, s), 3.67 (1H, dd, *J* = 8.3, 10.2 Hz), 3.67 – 3.75 (1H, m), 4.17 (1H, dd, *J* = 2.4, 12.2 Hz), 4.20 (1H, dd, *J* = 6.8, 12.6 Hz), 4.27 (1H, dd, *J* = 4.4, 12.2 Hz), 4.62 (1H, d, *J* = 9.8 Hz), 4.95 (1H, d, *J* = 7.8 Hz), 5.41 – 5.26 (3H, m), 5.92 (1H, ddd, *J* = 5.8, 11.2, 17.0 Hz); ^{13}C NMR (100 MHz, $CDCl_3$) δ 20.1, 20.2, 20.4, 29.4, 57.0, 61.2, 67.3, 67.4, 70.4, 70.5, 71.7, 71.8, 98.5, 98.6,118.6, 132.3, 169.1, 169.3, 170.2; $[M + Na]^+$ Calcd for $C_{16}H_{21}O_8NNa$, 378.1159; Found 378.1166.

9f. 9f was synthesized according to the general procedure in the main text (31% yield). $[\alpha]_D^{26}$ +21.8 (*c* 4.31, $CHCl_3$); IR (neat) 3481, 3024, 2972, 1754, 1718, 1613, 1468, 1428, 1384, 1335, 1217, 1170, 1144, 1113, 1072, 1029, 973, 932, 900, 873, 829, 795, 756, 725, 667, 640, 629, 602 cm^{-1}; ^1H NMR (400 MHz, $CDCl_3$) δ 0.83 (3H, d, *J* = 5.8 Hz), 1.10 (3H, d, *J* = 5.8 Hz), 1.81 (3H, s), 1.98 (3H, s), 2.05 (3H, s), 3.85 – 3.80 (2H, m), 4.11 (1H, dd, *J* = 2.4, 12.2 Hz), 4.24 (1H, dd, *J* = 8.4, 10.7 Hz), 4.27 (1H, dd, *J* = 4.8, 12.2 Hz), 5.11 (1H, t, *J* = 9.2 Hz), 5.38 (1H, d, *J* = 8.4 Hz), 5.74 (1H, dd, *J* = 9.2, 10.7 Hz), 7.70 (2H, dd, *J* = 2.9, 5.3 Hz), 7.81 (2H, dd, *J* = 3.4, 5.8 Hz); ^{13}C NMR (100 MHz, $CDCl_3$) δ 20.7, 20.9, 21.0, 22.0, 23.4, 55.1, 62.5, 69.4, 71.1, 71.9, 72.8, 97.07, 123.8, 131.6, 134.5, 169.7, 170.4, 170.9; HRMS (ESI) m/z: $[M + Na]^+$ Calcd for $C_{23}H_{27}O_{10}NNa$, 500.1527; Found 500.1525.

7f. 7f was synthesized according to the general procedure in the main text (31% overall yield). $[\alpha]_D^{26}$ –7.9 (*c* 2.23, $CHCl_3$); IR (neat) 3325, 2974, 2876, 1748, 1671, 1528, 1435, 1372, 1320, 1230, 1159, 1112, 1065, 1038, 986, 935, 904, 835, 754, 667 cm^{-1}; ^1H NMR (400 MHz, $CDCl_3$) δ 1.08 – 1.25 (6H, m), 2.01 (3H, s), 2.04 (3H, s), 2.06 (3H, s), 3.33 – 3.80 (1H, m), 3.89 – 4.00 (1H, m), 4.08 – 4.14 (1H, m), 4.23 – 4.31 (1H, m), 4.38 – 4.42 (1H, m), 4.82 (1H, d, *J* = 8.3 Hz), 4.96 – 5.10 (1H, m), 5.40 (1H, t, *J* = 10.7 Hz), 5.78 – 6.13 (1H, m) 7.98 – 8.14 (1H, m); ^{13}C NMR (100 MHz, $CDCl_3$) δ 20.5, 20.6, 20.7, 20.7, 21.8, 21.9, 23.2, 23.2, 57.1, 62.0, 62.3, 68.4, 68.4, 71.6,

71.6, 71.9, 72.0, 72.6, 73.4, 98.9, 100.1, 161.0, 165.3, 169.4, 169.5, 170.6, 170.7, 170.8; HRMS (ESI) m/z: $[M + Na]^+$ Calcd for $C_{16}H_{25}O_9NNa$, 398.1421; Found 398.1419.

6f. 6f was synthesized according to the general procedure in the main text (49% yield). $[\alpha]_D^{24}$ +27.0 (*c* 1.02, $CHCl_3$); IR (neat) 3852, 3837, 3801, 3734, 3674, 3648, 3628, 2976, 2146, 1749, 1683, 1652, 1635, 1558, 1540, 1520, 1507, 1456, 1373, 1224, 1107, 1055, 965, 772 cm^{-1}; ^1H NMR (400 MHz, $CDCl_3$) δ 1.23 (1H, d, *J* = 6.3 Hz), 1.27 (1H, d, *J* = 6.3 Hz), 2.02 (3H, s), 2.06 (3H, s), 2.10 (3H, s), 3.60 (1H, dd, *J* = 7.8, 10.2 Hz), 3.70 (1H, ddd, *J* = 2.4, 4.9, 10.3 Hz), 4.00 - 4.05 (1H, m), 4.25 (1H, dd, *J* = 4.9, 12.2 Hz), 4.61 (1H, d, *J* = 7.8 Hz), 4.91 (1H, t, *J* = 9.8 Hz), 5.30 (1H, t, *J* = 10.7 Hz); ^{13}C NMR (100 MHz, $CDCl_3$) δ 20.8, 21.9, 23.4, 58.0, 62.1, 68.1, 71.9, 72.2, 72.4, 72.5, 73.9, 99.3, 169.7, 169.9, 170.9; HRMS (ESI) m/z: $[M + Na]^+$ Calcd for $C_{16}H_{23}O_8NNa$, 380.1315; Found 380.1324.

9g. 9g was synthesized according to the general procedure in the main text (77% yield). $[\alpha]_D^{27}$ +30.4 (*c* 7.38, $CHCl_3$); IR (neat) 3484, 3027, 2956, 1752, 1719, 1387, 1227, 1164, 1082, 1043, 972, 900, 760, 723 cm^{-1}; ^1H NMR (400 MHz, $CDCl_3$) δ 1.86 (3H, s), 2.03 (3H, s), 2.12 (3H, s), 3.90 (1H, ddd, *J* = 1.5, 4.4, 11.2 Hz), 3.91 (1H, dd, *J* = 8.3, 11.2 Hz), 4.08 (1H, dd, *J* = 8.8, 12.7 Hz), 4.18 (1H, dd, *J* = 2.4, 12.7 Hz), 4.30 – 4.39 (2H, m), 5.18 (1H, dd, *J* = 9.3, 10.2 Hz), 5.49 (1H, d, *J* = 8.3 Hz), 5.49 (1H, d, *J* = 8.3 Hz), 5.81 (1H, dd, *J* = 8.9, 10.7 Hz), 7.74 (2H, dd, *J* = 3.4, 5.9 Hz), 7.85 (2H, dd, *J* = 3.4, 5.9 Hz); ^{13}C NMR (100 MHz, $CDCl_3$) δ 20.7, 20.9, 21.0, 54.5, 62.0, 66.2, 66.5, 68.9, 70.6, 72.4, 98.5, 123.9, 131.5, 134.7, 169.7, 170.4, 170.9; HRMS (ESI) m/z: $[M + Na]^+$ Calcd for $C_{22}H_{22}O_{10}NF_3Na$, 540.1088; Found 540.1079.

7g. 7g was synthesized according to the general procedure in the main text (37% overall yield). $[\alpha]_D^{27}$ +18.2 (*c* 0.12, $CHCl_3$); IR (neat) 3316, 2921, 2851, 1745, 1691, 1678, 1547, 1529, 1441, 1369, 1220, 1161, 1038, 965, 907, 773 cm^{-1}; ^1H NMR (400 MHz, $CDCl_3$) δ 2.03 (3H, s), 2.05 (3H, s), 2.09 (3H, s), 3.74 (1H, ddd, *J* = 2.4, 4.9, 9.8 Hz), 3.91 – 4.05 (1H, m), 4.10 – 4.20 (1H, m), 4.26 – 4.31 (1H, m), 4.47 (1H, d, *J* = 7.8 Hz), 5.04 – 5.28 (2H, m), 5.38 (1H, d, *J* = 10.7 Hz), 5.60 – 5.87 (1H, m), 8.17 (1H, s); ^{13}C NMR (100 MHz, $CDCl_3$) δ 20.8, 20.9, 21.0, 30.0, 50.5, 53.8, 57.0, 61.9, 62.0, 65.7, 67.9, 68.5, 68.9, 70.5, 71.8, 72.5, 98.1, 100.5, 161.1, 161.3, 169.6, 170.0, 171.0; $[M + Na]^+$ Calcd for $C_{15}H_{20}O_9NF_3Na$, 438.0982; Found 438.0978.

6g. 6g was synthesized according to the general procedure in the main text (54% yield). $[\alpha]_D^{27}$ +18.8 (*c* 0.17, $CHCl_3$); IR (neat) 2923, 2852, 2148, 1754, 1442, 1369, 1281, 1222, 1164, 1113, 1062, 970, 902, 772 cm^{-1}; ^1H NMR (400 MHz, $CDCl_3$) δ 2.03 (3H, s), 2.09 (3H, s), 2.12 (3H, s), 3.70 (1H, dd, *J* = 8.2, 10.7 Hz), 3.74 (1H, ddd, *J* = 2.0, 4.4, 10.7 Hz), 4.05 (1H, dd, *J* =

2.1, 8.3 Hz), 4.15 – 4.22 (2H, m), 4.27 (1H, dd, *J* = 4.4, 12.2 Hz), 4.71 (1H, d, *J* = 8.3 Hz), 4.96 (1H, t, *J* = 9.8 Hz), 5.32 (1H, dd, *J* = 9.3, 10.7 Hz); ^{13}C NMR (100 MHz, $CDCl_3$) δ 20.7, 20.8, 20.9, 61.6, 67.6, 72.0, 72.7, 76.9, 100.2, 131.2, 169.6, 169.8, 170.7; HRMS (ESI) m/z: $[M + Na]^+$ Calcd for $C_{15}H_{18}O_8NF_3Na$, 420.0876; Found 420.0876.

7h. 7h was synthesized according to the general procedure in the main text (24% overall yield). $[\alpha]_D^{25}$ –3.9 (*c* 1.02, $CHCl_3$); IR (neat) 3335, 2956, 2926, 2856, 2360, 1741, 1698, 1668, 1558, 1540, 1520, 1507, 1456, 1418, 1385, 1220, 1165, 1112, 1084, 1029 cm^{-1}; ^{1}H-NMR (400 MHz, $CDCl_3$) δ 0.85 – 0.89 (12H, m), 1.10 – 1.35 (27H, m), 1.54 – 1.62 (6H, m), 2.18 – 2.40 (6H, m), 3.62 – 3.80 (1H, m), 3.82 – 3.99 (1H, m), 4.12 – 4.24 (1H, m), 4.28 – 4.47 (1H, m), 4.64 – 4.82 (1H, m), 5.10 – 5.18 (1H, m), 5.35 – 5.59 (1H, m), 7.98 – 8.14 (1H, m); ^{13}C NMR (100 MHz, $CDCl_3$) δ 14.3, 14.4, 22.1, 22.2, 22.3, 22.9, 22.9, 23.5, 23.6, 23.6, 25.1, 25.2, 25.2, 25.2, 26.0, 29.2, 29.2, 29.2, 29.3, 29.3, 29.4, 29.5, 31.9, 31.9, 32.0, 34.3, 34.3, 34.4, 34.5, 34.5, 37.2, 54.8, 55.0, 57.6, 62.3, 62.5, 63.4, 68.3, 68.9, 69.7, 71.8, 72.1, 72.2, 72.3, 72.6, 72.8, 73.7, 74.4, 75.2, 99.4, 100.0, 100.6, 161.1, 165.3, 172.4, 172.4, 173.3, 173.8, 174.0, 174.8, 175.2; HRMS (ESI) m/z: $[M + Na]^+$ Calcd for $C_{34}H_{61}O_9NNa$, 650.4239; Found 650.4257.

6h. 6h was synthesized according to the general procedure in the main text (60% yield). $[\alpha]_D^{29}$ +15.1 (*c* 9.50, $CHCl_3$); IR (neat) 3351, 2956, 2928, 2857, 2146, 1818, 1746, 1670, 1520, 1465, 1416, 1382, 1224, 1162, 1109, 1061, 922, 724 cm^{-1}; ^{1}H NMR (400 MHz, $CDCl_3$) δ 0.83 – 0.92, (12H, m), 1.18 – 1.40 (27H, m), 1.55 – 1.64 (6H, m), 2.18 – 2.42 (6H, m), 3.60 (1H, dd, *J* = 8.3, 10.7 Hz), 3.70 (1H, ddd, *J* = 2.4, 4.9, 9.8 Hz), 3.97 – 4.04 (1H, m), 4.11 (1H, dd, *J* = 2.4, 12.2 Hz), 4.19 (1H, dd, *J* = 2.4, 12.2 Hz), 4.61 (1H, d, *J* = 7.8 Hz), 4.93 (1H, t, *J* = 9.8 Hz), 5.32 (1H, t, *J* =10.7 Hz); ^{13}C NMR (100 MHz, $CDCl_3$) δ 14.0, 14.1, 21.6, 22.5, 23.1, 24.6, 24.7, 28.8, 28.9, 29.0, 31.5, 31.6, 33.7, 33.9, 57.8, 61.7, 67.5, 71.8, 72.0, 73.5, 76.6, 98.9, 172.1, 172.4, 173.3; HRMS (ESI) m/z: $[M + Na]^+$ Calcd for $C_{34}H_{59}O_8NNa$, 632.4133; Found 632.4127.

7i. 7i was synthesized according to the general procedure in the main text (16% overall yield). $[\alpha]_D^{23}$ +6.5 (*c* 0.20, $CHCl_3$); IR (neat) 3306, 2918, 2850, 1739, 1665, 1538, 1468, 1372, 1243, 1222, 1163, 1036, 720 cm^{-1}; ^{1}H NMR ($CDCl_3$, 400 MHz) δ 0.87 (9H, t, *J* = 6.8 Hz), 1.12-1.18 (78H, m), 1.62-1.80 (6H, m), 2.21-2.33 (6H, m), 3.92 (1H, septet, *J* = 6.3 Hz), 4.06-4.19 (2H, m), 4.78 (1H, d, *J* = 8.3 Hz), 5.04 (1H, t, *J* = 9.8 Hz), 5.37 (1H, dd, *J* = 10.7, 9.3 Hz), 5.43 (1H, d, *J* = 8.3 Hz), 5.65 (1H, m), 8.14 (1H, s); ^{13}C NMR ($CDCl_3$, 100 MHz) δ 14.1, 14.2, 22.0, 22.67, 22.72, 23.27, 23.31, 24.78, 24.79, 24.9, 29.1, 29.27, 29.28, 29.3, 29.5, 29.65, 29.69, 30.2, 31.91, 31.95, 34.0, 34.1, 55.5, 62.3, 68.6, 71.79, 71.82, 72.5, 99.3, 170.0, 172.1, 173.5, 173.6; HRMS (ESI) m/z: $[M + Na]^+$; Calcd for $C_{57}H_{113}O_9NNa$ 978.8308; Found 978.8330.

6i. 6i was synthesized according to the general procedure in the main text (54% yield). $[\alpha]_D^{23}$ +8.3 (*c* 0.03, $CHCl_3$); IR (neat) 2917, 2849, 2350, 2148, 1749, 1559, 1541, 1507, 1463, 1147, 1046 cm^{-1}; ^1H NMR ($CDCl_3$, 400 MHz) δ 0.88 (9H, t, *J* = 6.3 Hz), 1.25 (78H, s), 1.59 (6H, s), 2.19-2.39 (6H, m), 3.60 (1H, dd, *J* = 10.2, 8.3 Hz), 3,71 (1H, ddd, *J* = 12.2, 9.8, 2.4 Hz), 4.01 (1H, septet, *J* = 5.9 Hz), 4.11 (1H, dd, *J* = 12.2, 2.0 Hz), 4.19 (1H, dd, *J* = 12.2, 4.9 Hz), 4.91 (1H, d, *J* = 8.3 Hz), 4.93 (1H, dd, *J* = 9.8, 9.3 Hz), 5.32 (1H, dd, *J* = 10.3, 9.8 Hz); ^{13}C NMR ($CDCl_3$, 100 MHz) δ 14.12, 14.14, 21.7, 22.68, 22.70, 23.2, 24.68, 24.79, 24.83, 29.11, 29.13, 29.28, 29.30, 29.4, 29.47, 29.50, 29.65, 29.69, 29.7, 31.92, 31.94, 33.9, 34.0, 57.8, 61.7, 67.6, 71.8, 72.1, 73.6, 99.0, 160.3, 172.2, 172.4, 173.3; HRMS (ESI) m/z: $[M + Na]^+$; Calcd for $C_{58}H_{107}O_8NNa$ 968.7889; Found 968.7903.

7j. 7j was synthesized according to the general procedure in the main text (46% overall yield). $[\alpha]_D^{27}$ –19.4 (*c* 0.28, $CHCl_3$); IR (neat) 3307, 3065, 2974, 2874, 2360, 2341, 1725, 1631, 1601, 1584, 1537, 1491, 1452, 1384, 1315, 1270, 1177, 1111, 1069, 1026, 985, 936, 852, 803, 755, 709, 686 cm^{-1}; ^1H-NMR (400 MHz, $CDCl_3$) δ 1.19 (3H, d, *J* = 5.9 Hz), 1.25 (3H, d, *J* = 5.9 Hz), 3.95 – 4.15 (2H, m), 4.46 (1H, ddd, *J* = 3.9, 9.2, 12.2 Hz), 4.55 - 4.64 (2H, m), 5.03 (1H, d, J = 9.8 Hz), 5.55 – 5.78 (2H, m), 5.87 – 6.00 (1H, m), 7.28 – 7.42 (6H, m), 7.42 – 7.60 (3H, m), 7.85 – 8.08 (6H, m), 8.15 (1H,s); ^{13}C NMR (100 MHz, $CDCl_3$) δ 21.5, 21.6, 21.9, 22.9, 54.5, 57.2, 60.1, 63.1, 69.7, 71.5, 71.6, 71.9, 72, 73.3, 98.9, 100.2, 127.9, 128.1, 129.4, 132.7, 133.2, 160.8, 164.9, 165.7, 166.0; HRMS (ESI) m/z: $[M + Na]^+$ Calcd for $C_{31}H_{31}O_9NNa$, 584.1891; Found 584.1903.

6j. 6j was synthesized according to the general procedure in the main text (35% yield). $[\alpha]_D^{29}$ –26.2 (*c* 0.30, $CHCl_3$); IR (neat) 3064, 2975, 2877, 2145, 1729, 1601, 1452, 1316, 1267, 1177, 1108, 1068, 1025, 708 cm^{-1}; ^1H NMR (400 MHz, $CDCl_3$) δ 1.29 (3H, d, *J* = 6.3 Hz), 1.29 (3H, d, *J* = 5.9 Hz), 3.87 (1H, dd, *J* = 7.8, 10.7 Hz), 4.14 – 4.04 (2H, m), 4.45 (1H, dd, *J* = 5.9, 12.2 Hz), 4.57 (1H, dd, *J* = 3.4, 12.2 Hz), 4.82 (1H, d, *J* = 7.8 Hz), 5.45 (1H, t, *J* = 9.8 Hz), 5.85 (1H, t, *J* = 9.8 Hz), 7.30 – 7.42 (6H, m), 7.47 – 7.56 (3H, m), 7.87 (4H, m), 7.96 (2H, m); ^{13}C NMR (100 MHz, $CDCl_3$) δ 22.0, 23.4, 58.3, 63.3, 69.3, 72.4, 72.6, 74.1, 99.5, 128.6, 128.7, 128.8, 129.7, 129.9, 130.1, 130.2, 133.4, 133.9, 165.4, 165.4, 166.3; HRMS (ESI) m/z: $[M + Na]^+$ Calcd for $C_{31}H_{29}O_8NNa$, 566.1758; Found 566.1787.

7k. 7k was synthesized according to the general procedure in the main text (43% overall yield). $[\alpha]_D^{29}$ –13.8 (*c* 0.24, $CHCl_3$); IR (neat) 3091, 3853, 3838, 3819, 3750, 3734, 3674, 3648, 3628, 3586, 3566, 2954, 2930, 2885, 2857, 1698, 1684, 1653, 1617, 1558, 1540, 1520, 1507, 1473, 1457, 1386, 1361, 1255, 1095, 1065, 1039, 836, 776, 669 cm^{-1}; ^1H NMR (400 MHz, $CDCl_3$) δ

0.03 (3H, s), 0.04 (3H, s), 0.09 (3H, s), 0.11 (3H, s), 0.11 (3H, s), 0.12 (3H, s), 0.88 (9H, s), 0.89 (9H, s), 0.91 (9H, s), 1.10 (3H, d, J = 6.3 Hz), 1.14 (1H, d, J = 6.3 Hz), 3.66 (1H, dd, J = 4.9, 9.8 Hz), 3.72 – 3.80 (2H, m), 3.90 (1H, dd, J = 4.9, 11.2 Hz), 3.94 – 3.97 (1H, m), 4.08 (1H, d, J = 9.3 Hz), 4.32 (1H, t, J = 9.8 Hz), 4.60 – 4.72 (1H, m), 6.86 (1H, d, J = 9.5 Hz), 8.10 (1H, s); ^{13}C NMR (100 MHz, $CDCl_3$) δ –5.4, –5.3, –5.1, –5.0, –5.0, –4.8, 17.8, 17.9, 18.1, 21.6, 23.0, 25.6, 25.8, 25.8, 25.9, 49.3, 63.9, 68.4, 69.5, 70.1, 71.0, 78.7, 96.1, 159.7; HRMS (ESI) m/z: $[M + Na]^+$ Calcd for $C_{28}H_{61}O_6NNaSi_3$, 614.3699; Found 614.3697.

6k. 6k was synthesized according to the general procedure in the main text (27% yield).
$[\alpha]_D^{27}$ +6.7 (*c* 1.25, $CHCl_3$); IR (neat) 2959, 2933, 2886, 2855, 2740, 2711, 2140, 1736, 1470, 1408, 1384, 1358, 1346, 1257, 1100, 977, 939, 879, 836, 780, 673, 616 cm^{-1}; ^{1}H NMR (400 MHz, $CDCl_3$) δ 0.04 (3H, s), 0.06 (3H, s), 0.11 (3H, s), 0.12 (3H, s), 0.12 (3H, s), 0.16 (3H, s), 0.88 (9H, s), 0.90 (9H, s), 0.92 (9H, s), 1.22 (3H, d, J = 6.3 Hz), 1.25 (1H, d, J = 6.3 Hz), 3.43 (1H, dd, J = 2.4, 7.3 Hz), 3.69 – 3.79 (2H, m), 3.83 (1H, dd, J = 1.5, 3.9 Hz), 3.97 (1H, t, J = 6.3 Hz), 3.96 – 4.04 (1H, m), 4.96 (1H, d, J = 7.8 Hz); ^{13}C NMR (100 MHz, $CDCl_3$) δ –5.3, –4.6, –4.5, –4.4, –4.3, 1.0, 17.8, 18.0, 18.2, 21.5, 23.2, 25.7, 25.8, 63.4, 69.5, 71.9, 81.6, 97.3; HRMS (ESI) m/z: $[M + Na]^+$ Calcd for $C_{28}H_{59}O_5NNaSi_3$, 596.3693; Found 596.3593.

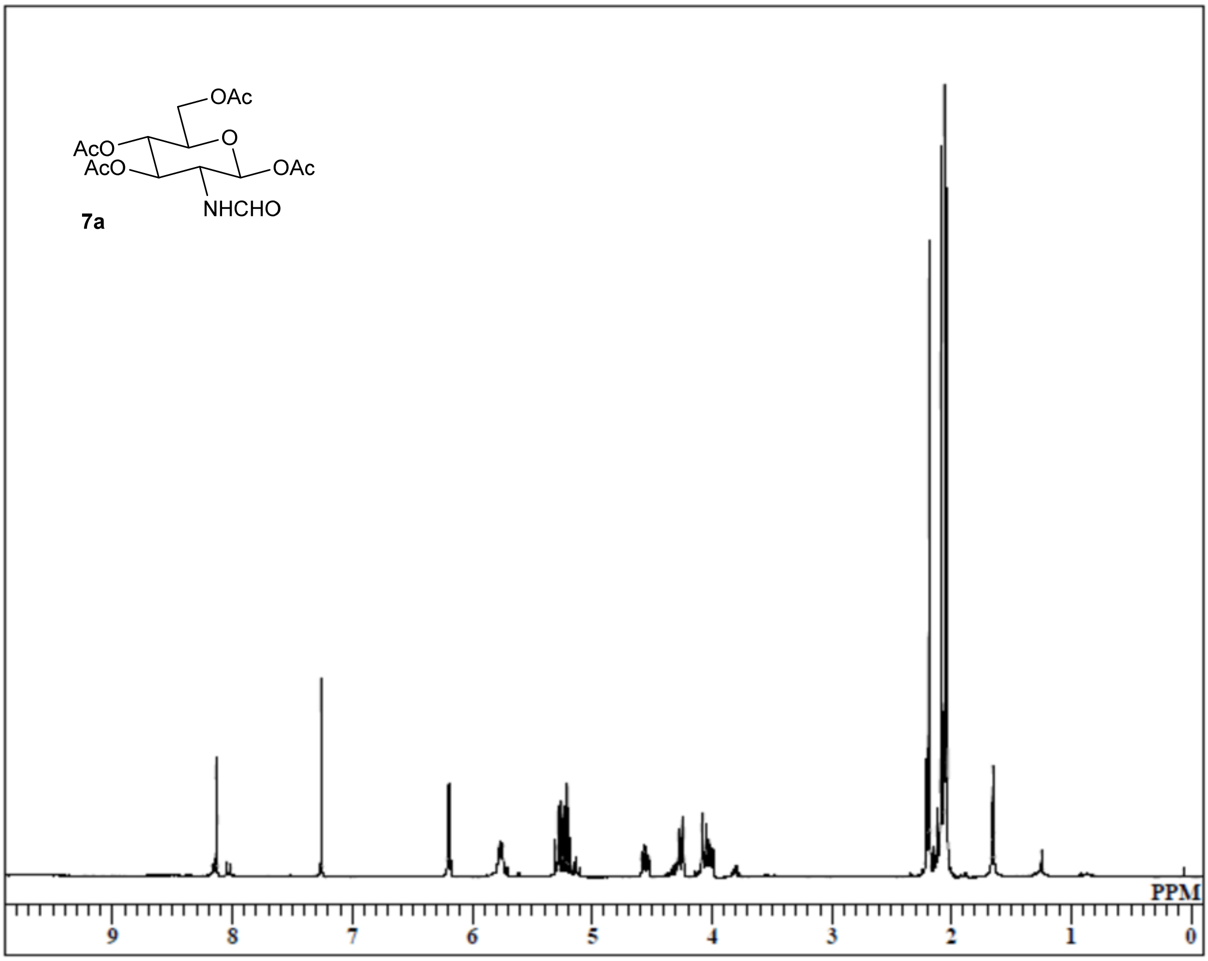
OAc
AcO
AcO
O
OAc
NHCHO
7a
PPM
9
8
7
6
5
4
3
2
1
0

7a

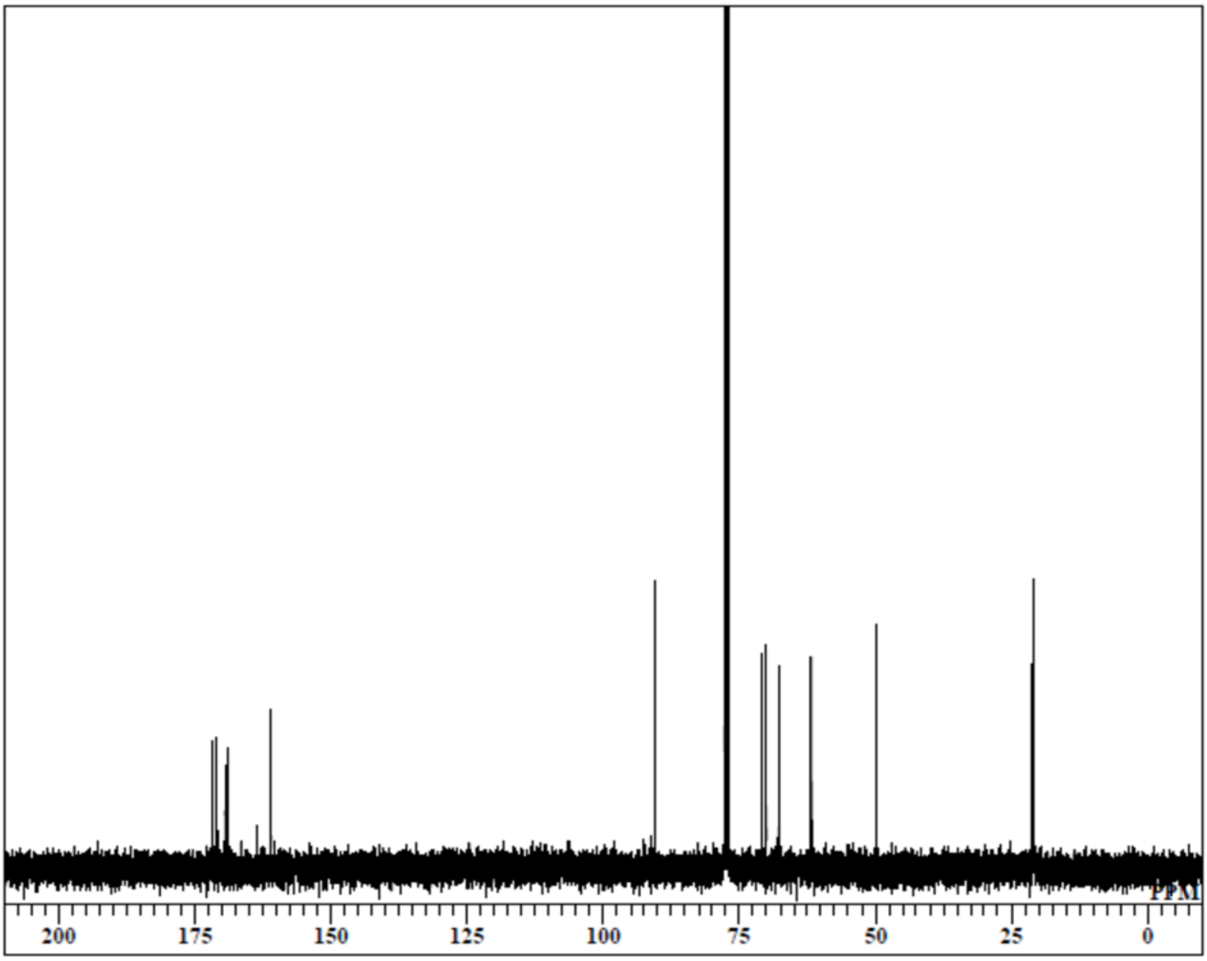
PPM
200
175
150
125
100
75
50
25
0

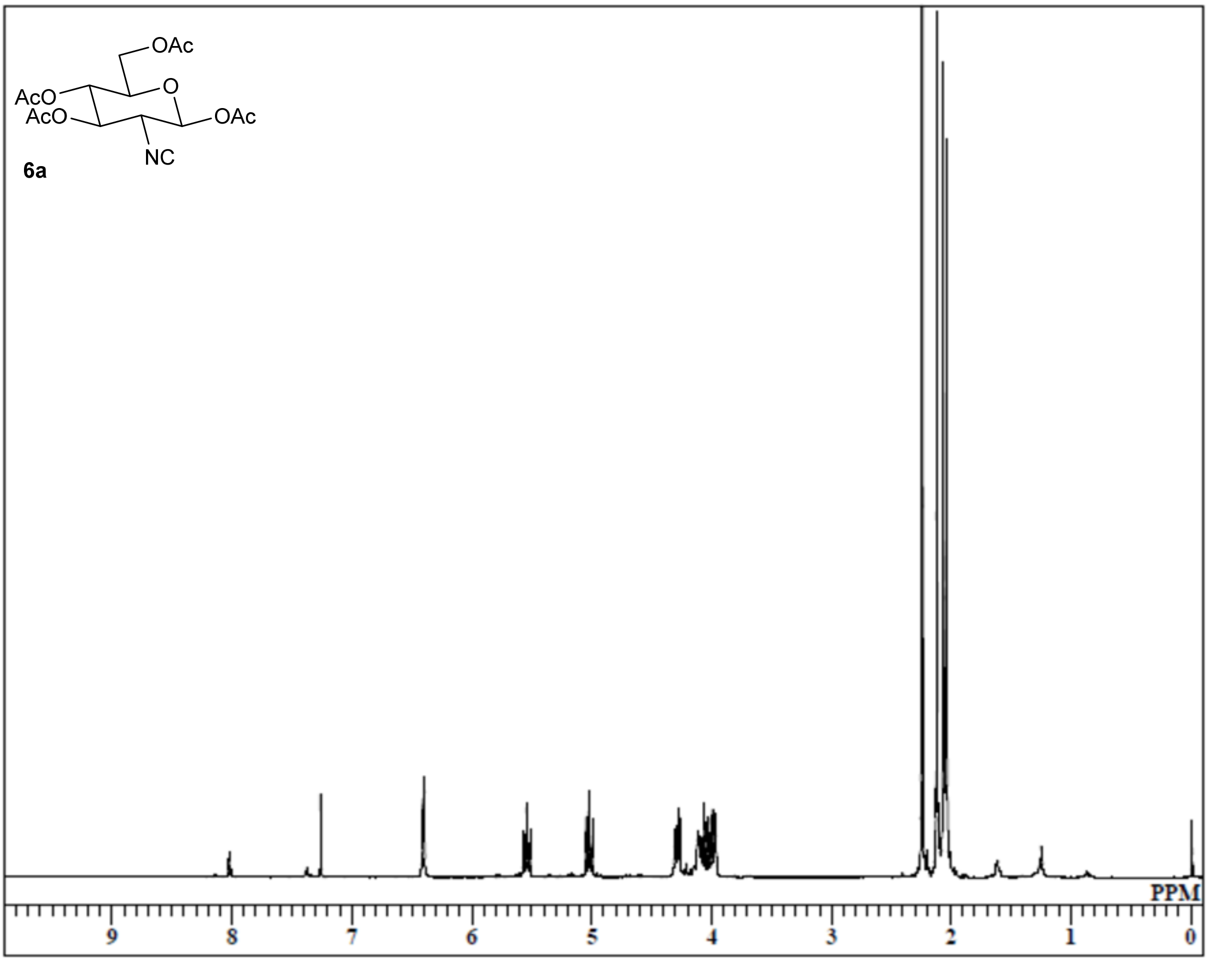
OAc
AcO
AcO
O
OAc
NC
6a
PPM
9
8
7
6
5
4
3
2
1
0

6a

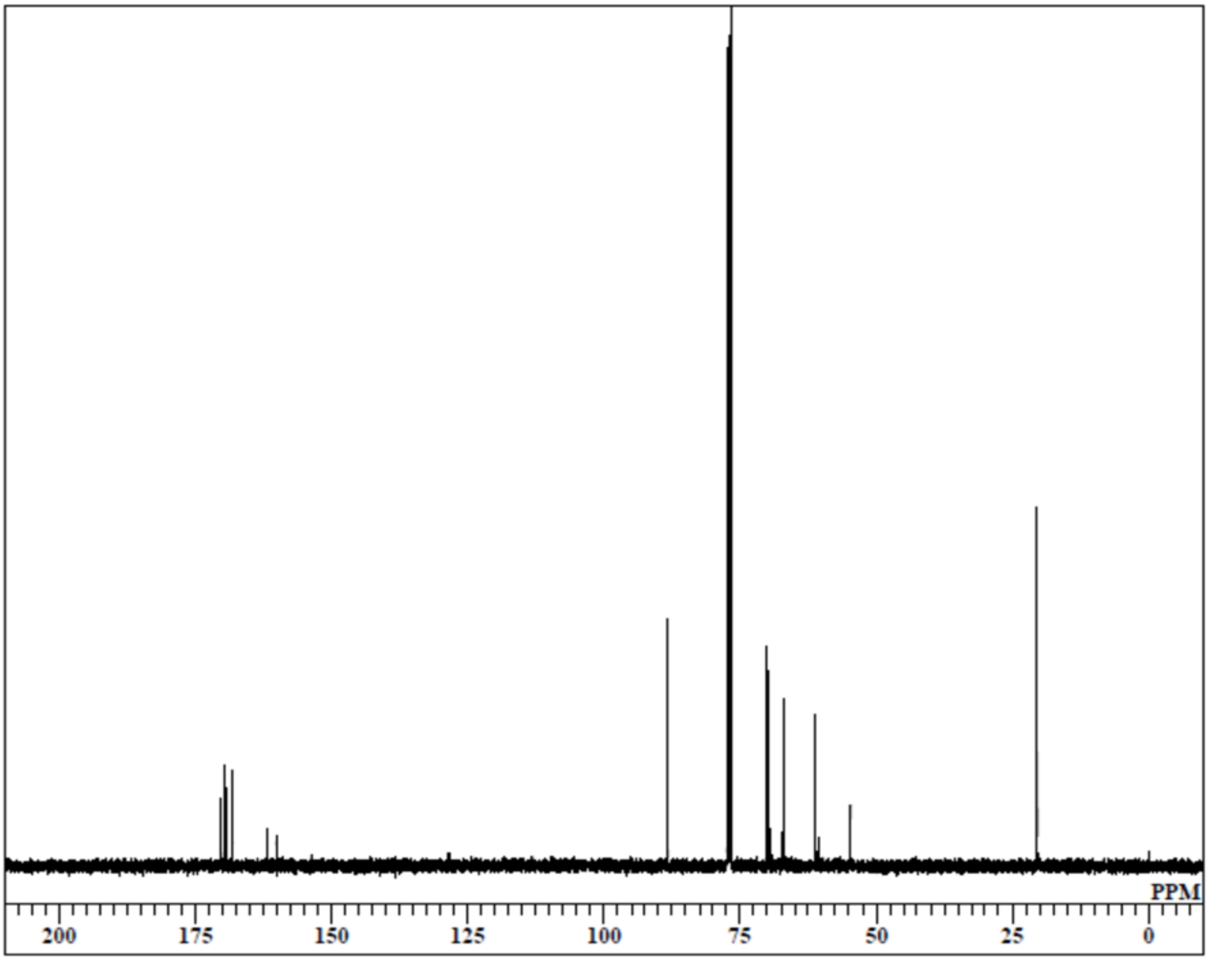
PPM
200
175
150
125
100
75
50
25
0

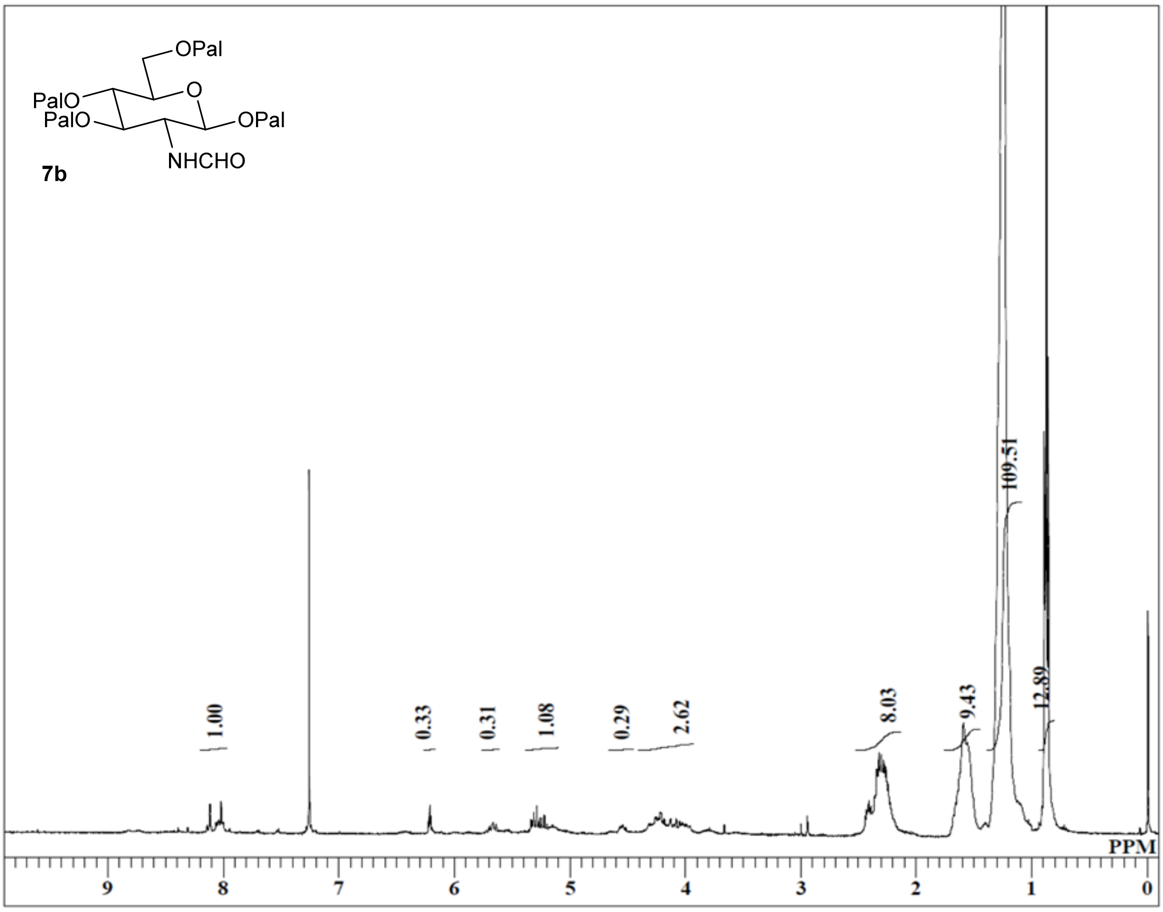
OPal
PalO
PalO
O
OPal
NHCHO
7b
1.00
0.33
0.31
1.08
0.29
2.62
8.03
9.43
109.51
12.89
PPM
9
8
7
6
5
4
3
2
1
0

7b

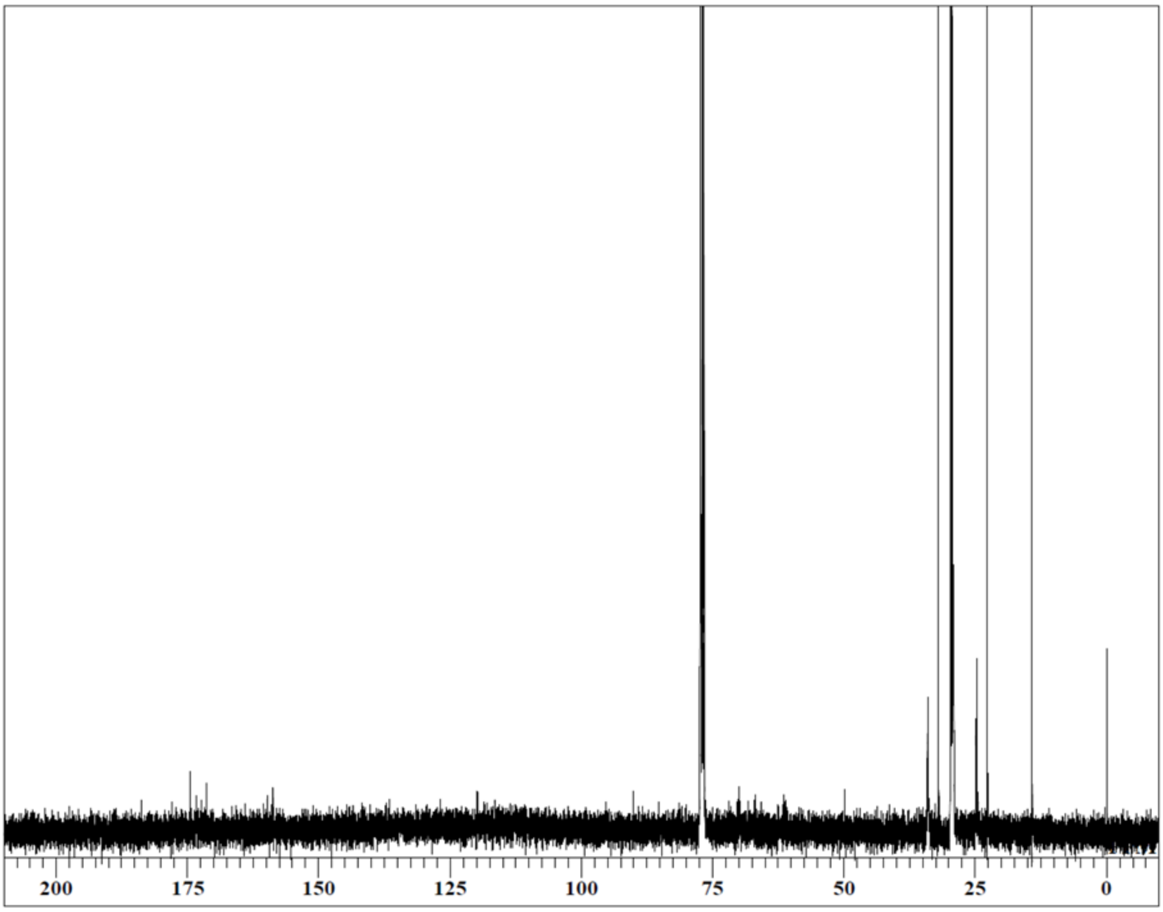
200
175
150
125
100
75
50
25
0

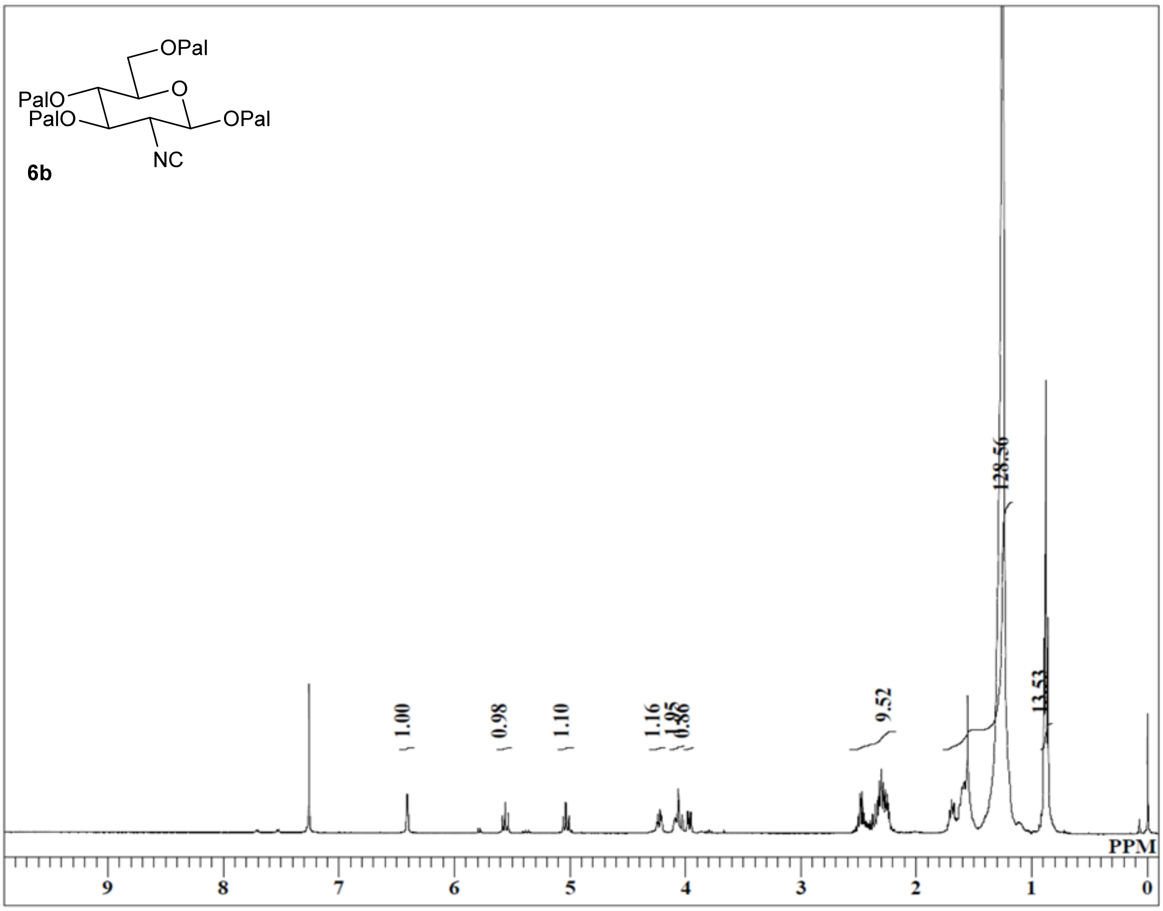
OPal
PalO
PalO
O
OPal
NC
6b
1.00
0.98
1.10
1.16
1.05
0.86
9.52
128.56
13.53
PPM
9
8
7
6
5
4
3
2
1
0

6b

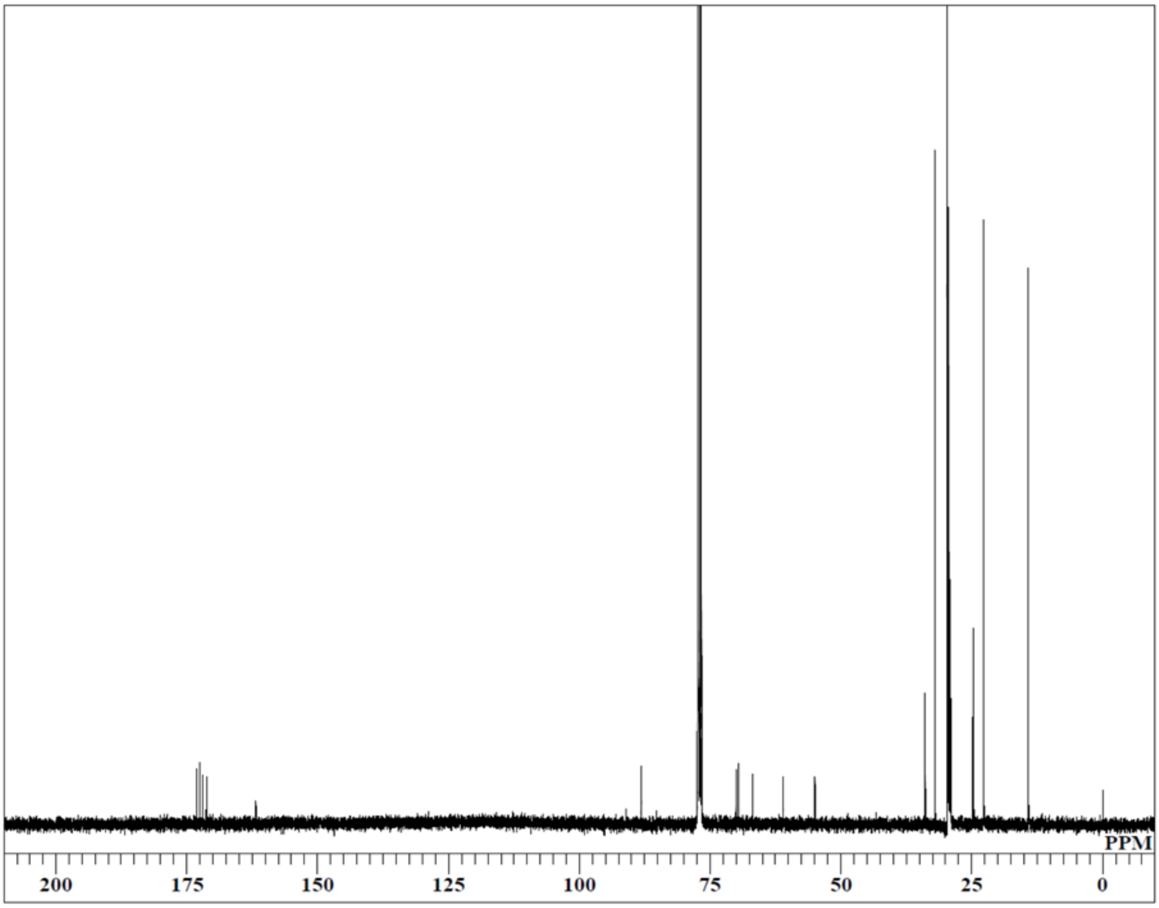
PPM
200
175
150
125
100
75
50
25
0

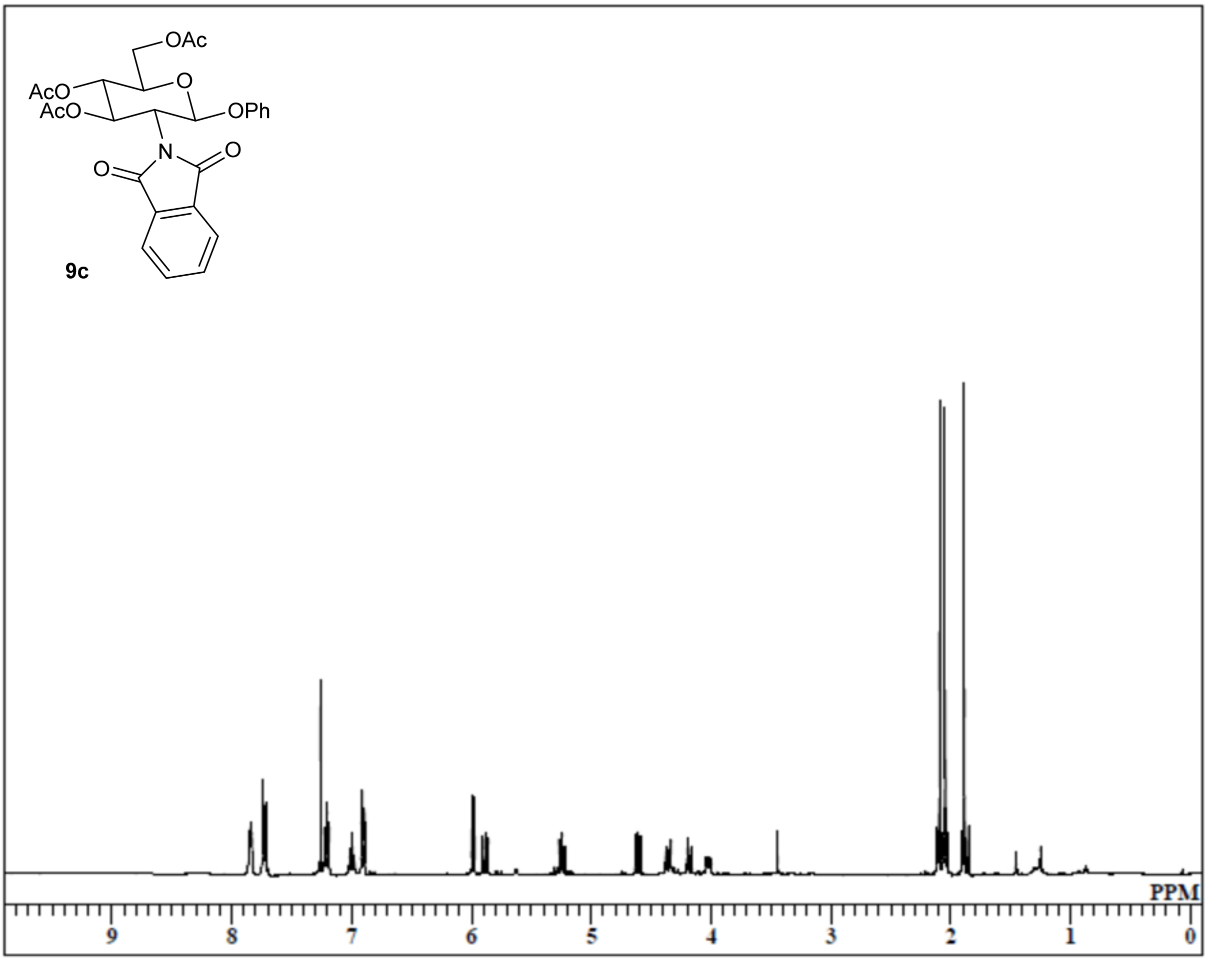
OAc
AcO
AcO
O
OPh
N
O
O
9c
PPM
9
8
7
6
5
4
3
2
1
0

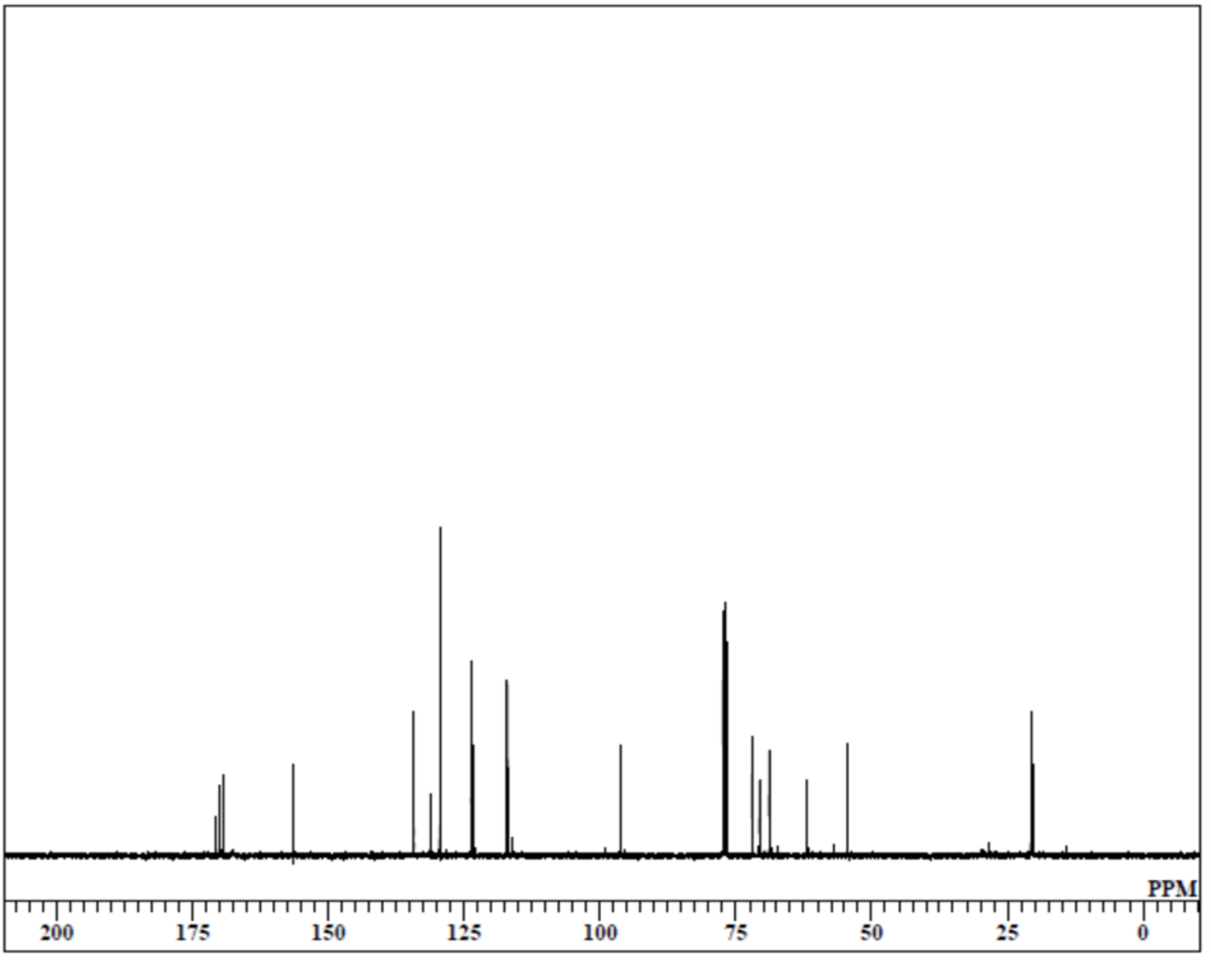
PPM
200
175
150
125
100
75
50
25
0

9c

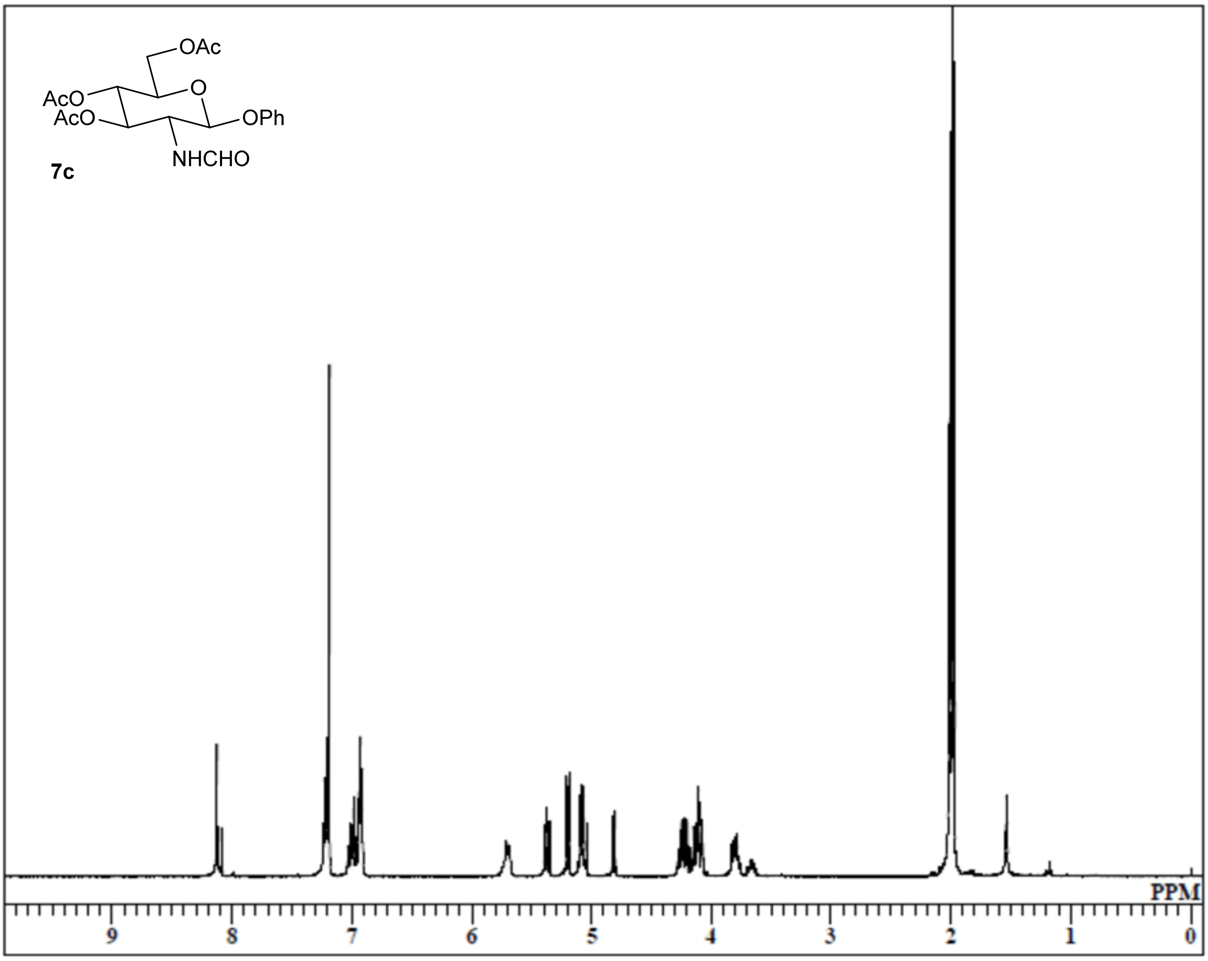
OAc
AcO
AcO
O
OPh
NHCHO
7c
PPM
9
8
7
6
5
4
3
2
1
0

7c

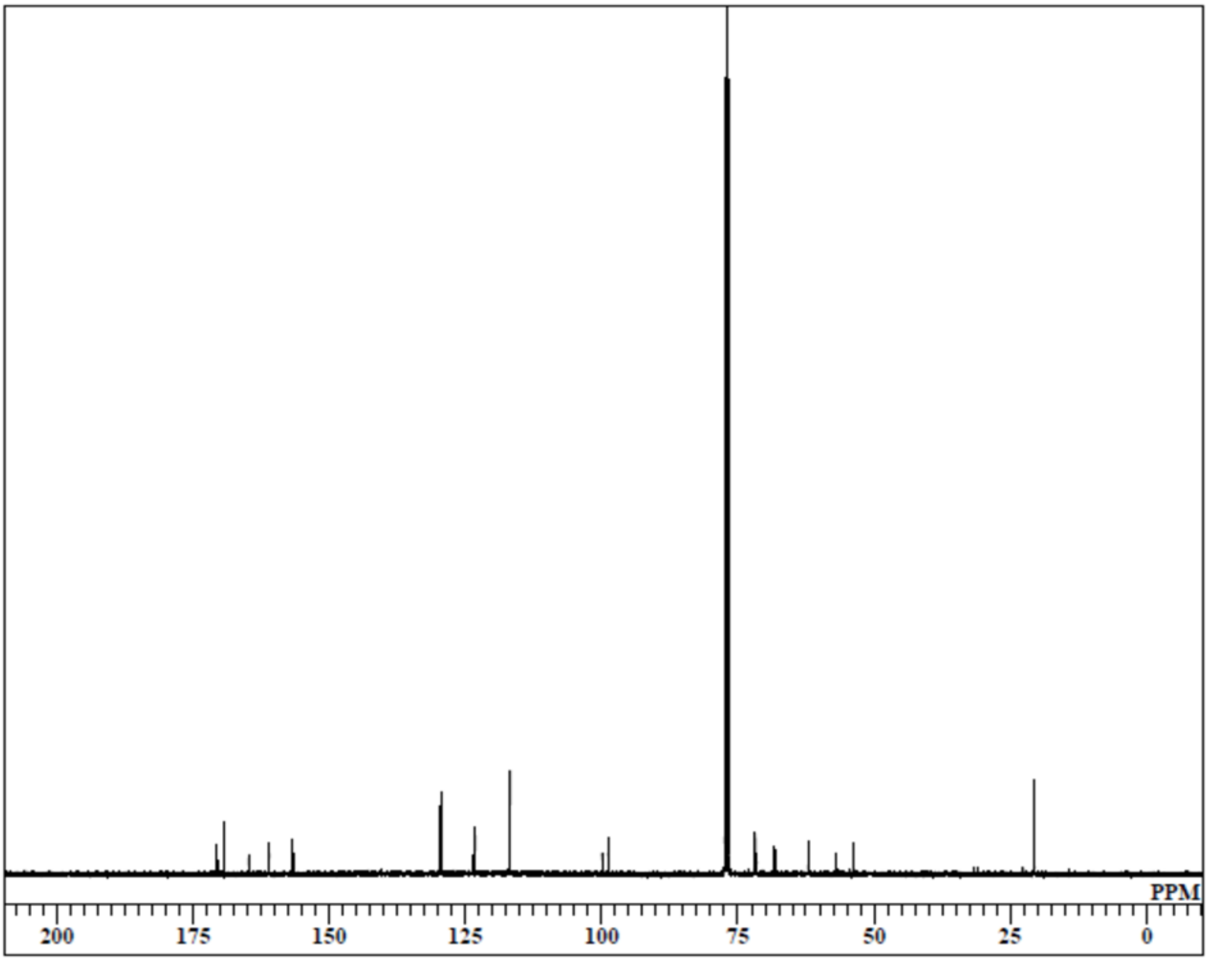
PPM
200
175
150
125
100
75
50
25
0

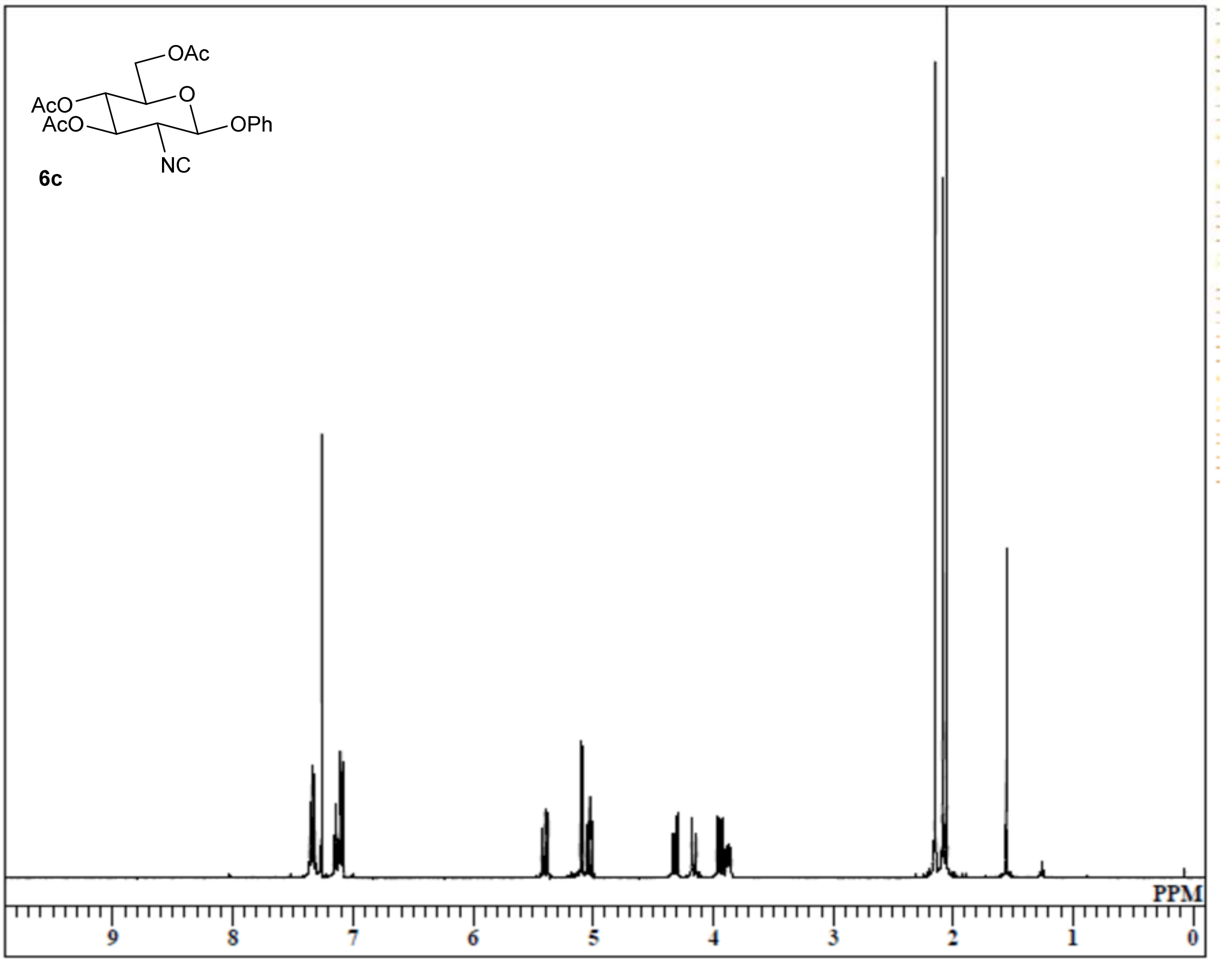
OAc
AcO
AcO
O
OPh
NC
6c
PPM
9 8 7 6 5 4 3 2 1 0

6c

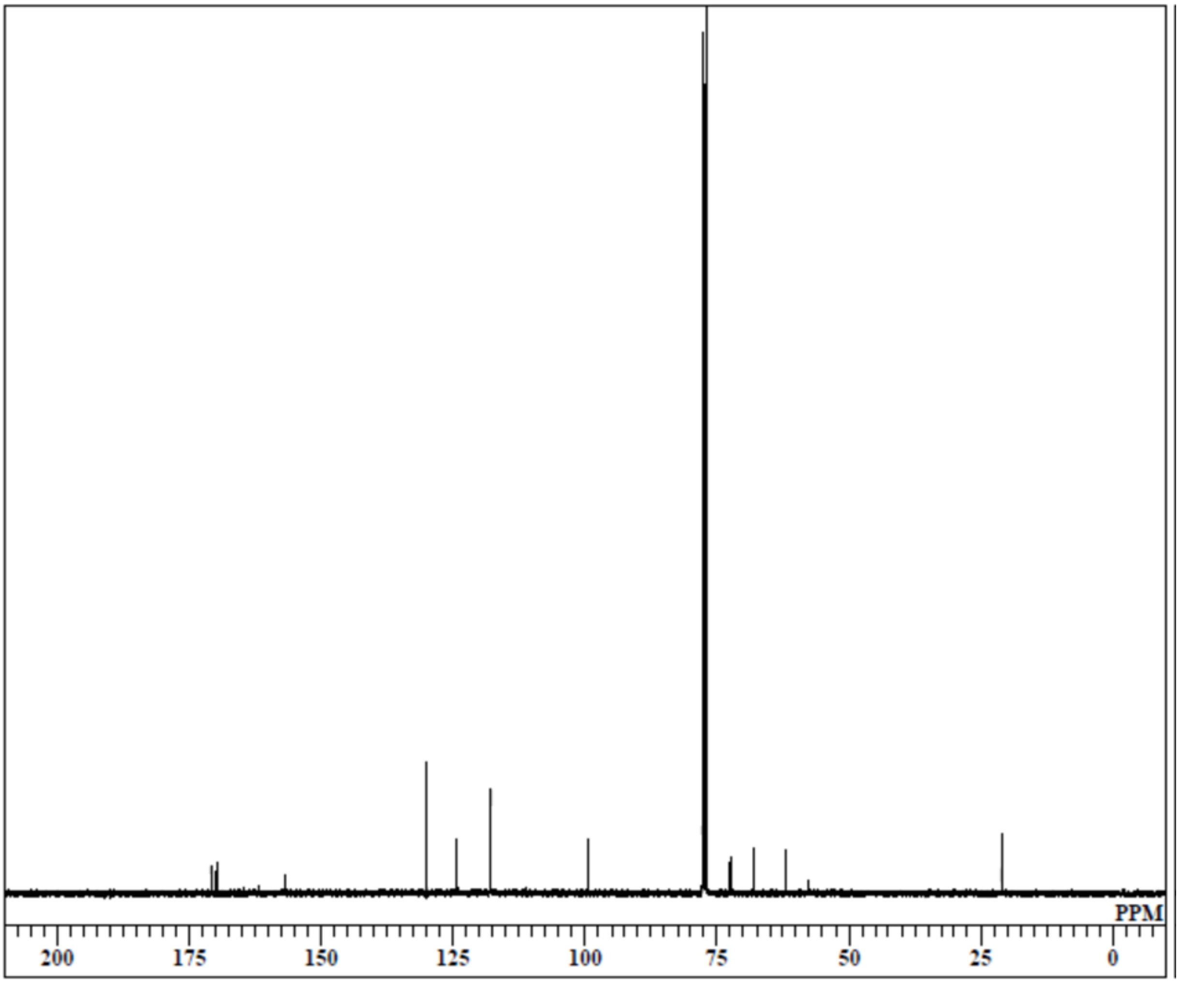
PPM
200 175 150 125 100 75 50 25 0

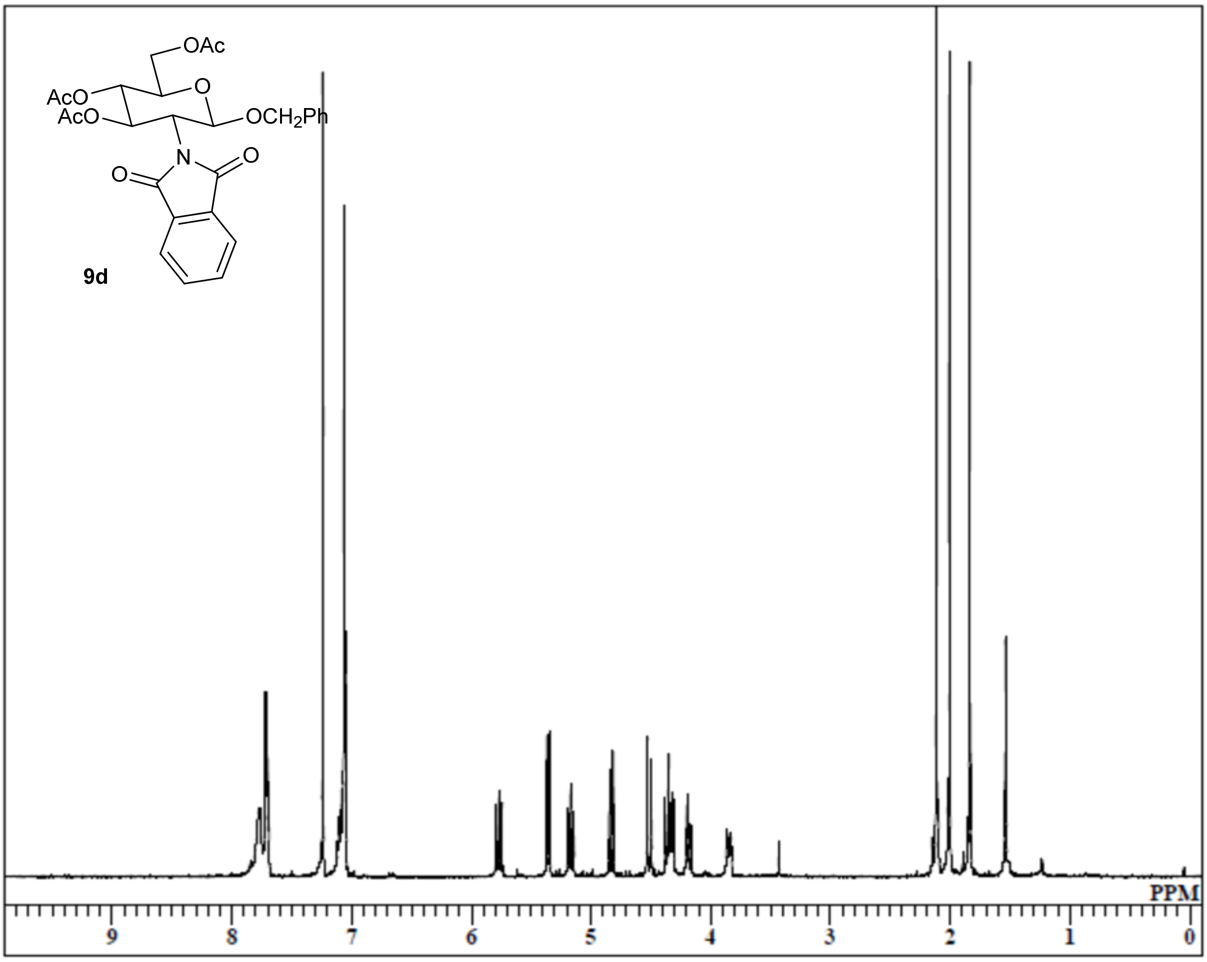
OAc
AcO
AcO
O
OCH2Ph
N
O
O
9d
PPM
9
8
7
6
5
4
3
2
1
0

9d

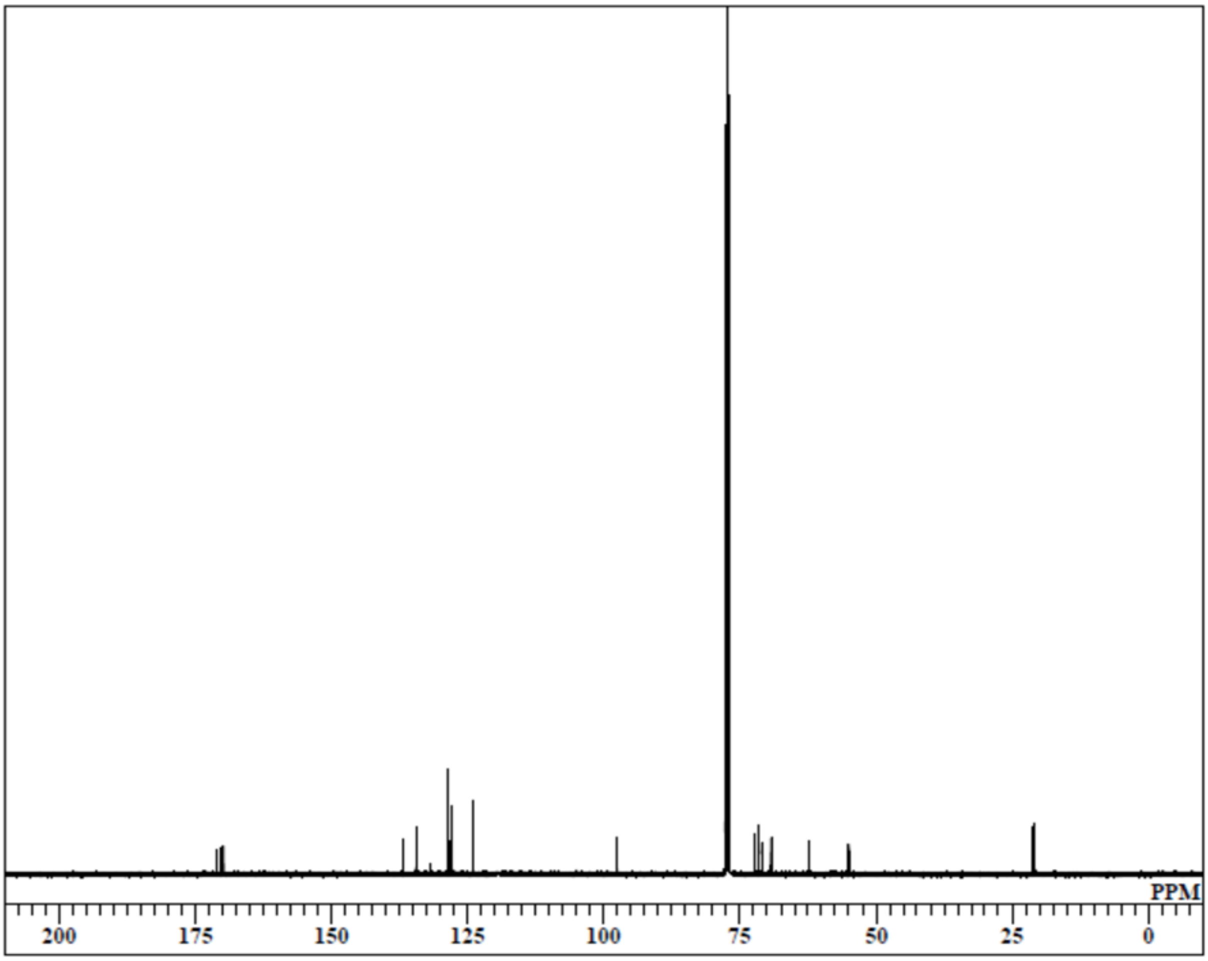
PPM
200
175
150
125
100
75
50
25
0

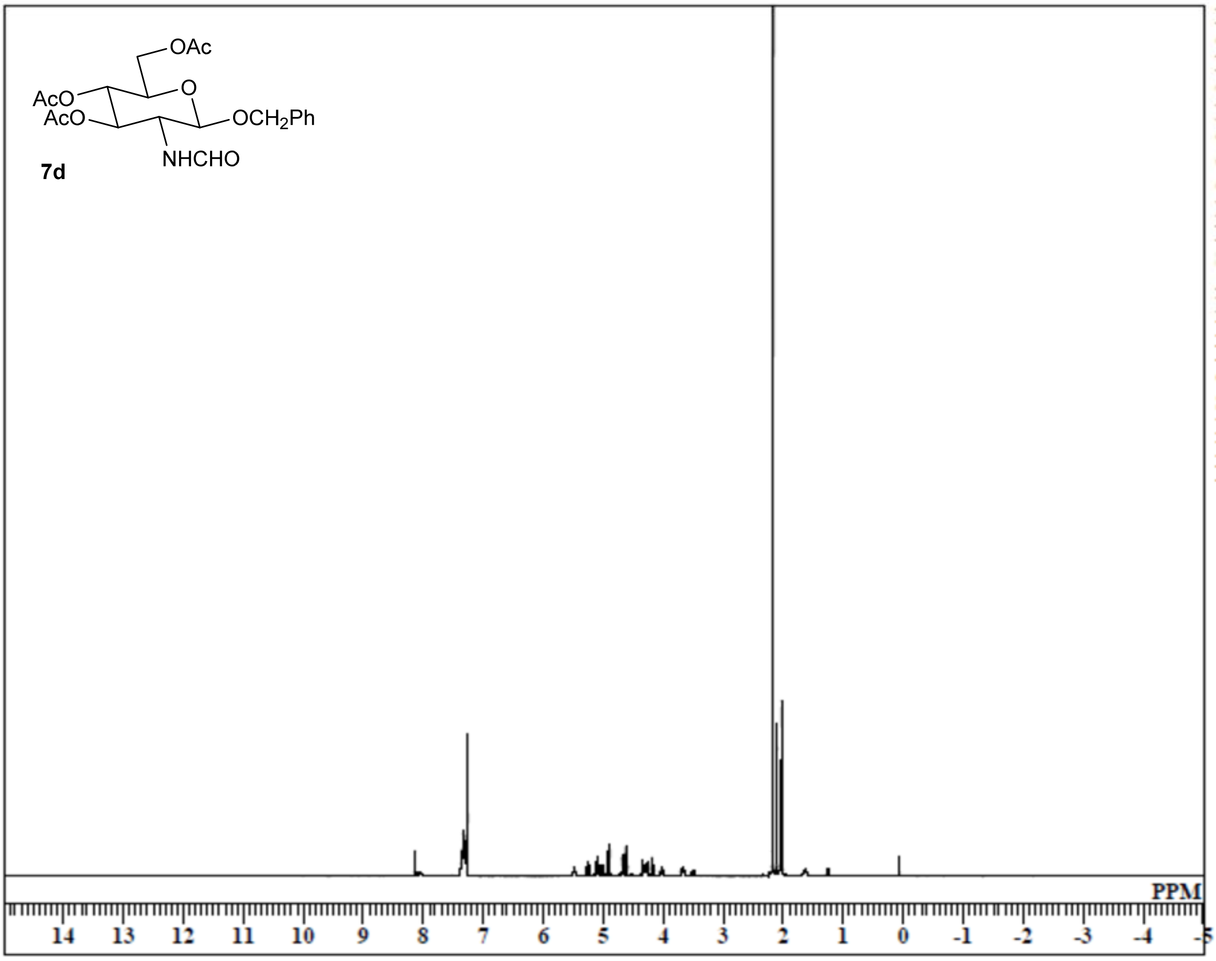
OAc
AcO
AcO
O
OCH2Ph
NHCHO
7d
PPM
14 13 12 11 10 9 8 7 6 5 4 3 2 1 0 -1 -2 -3 -4 -5

7d

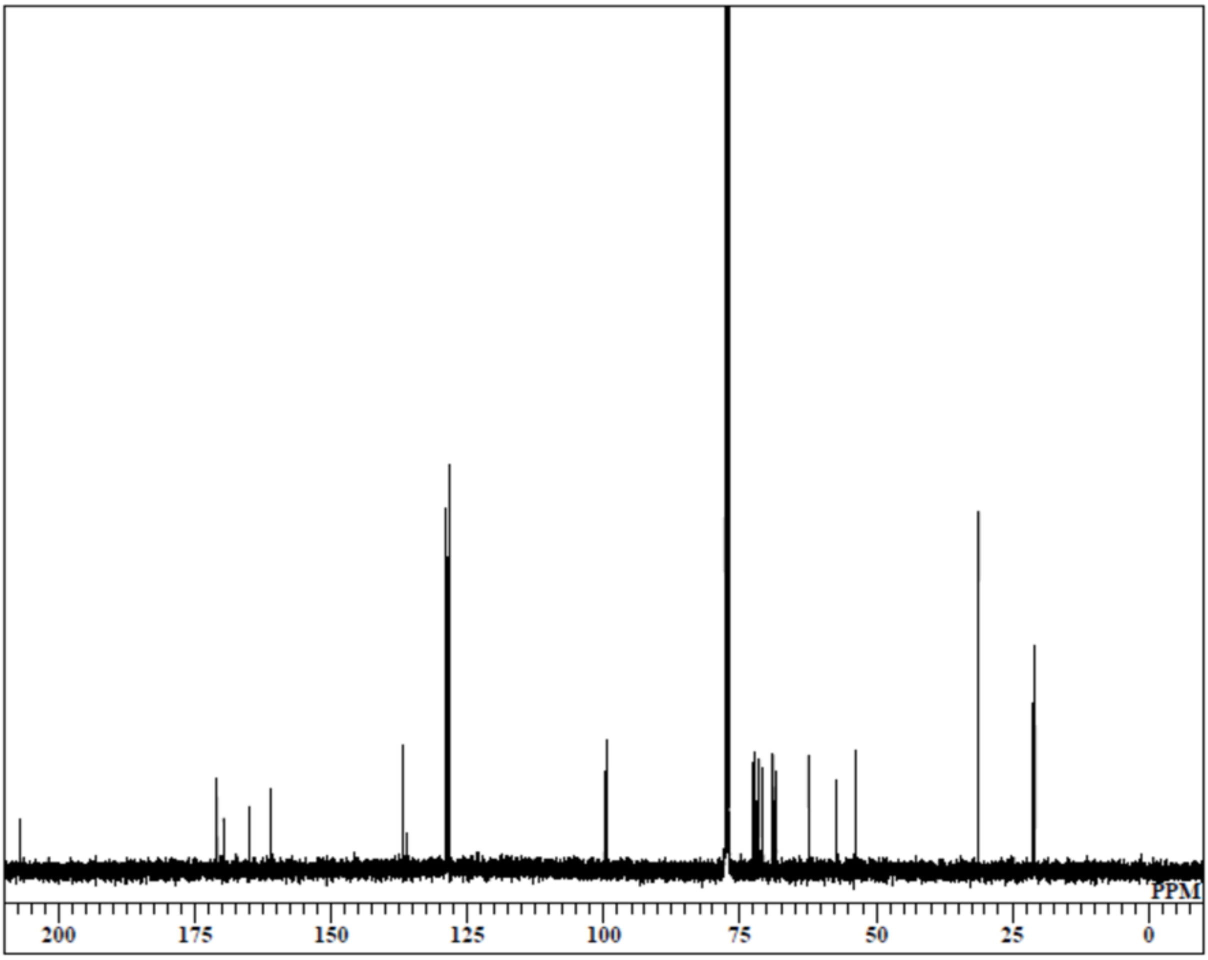
PPM
200 175 150 125 100 75 50 25 0

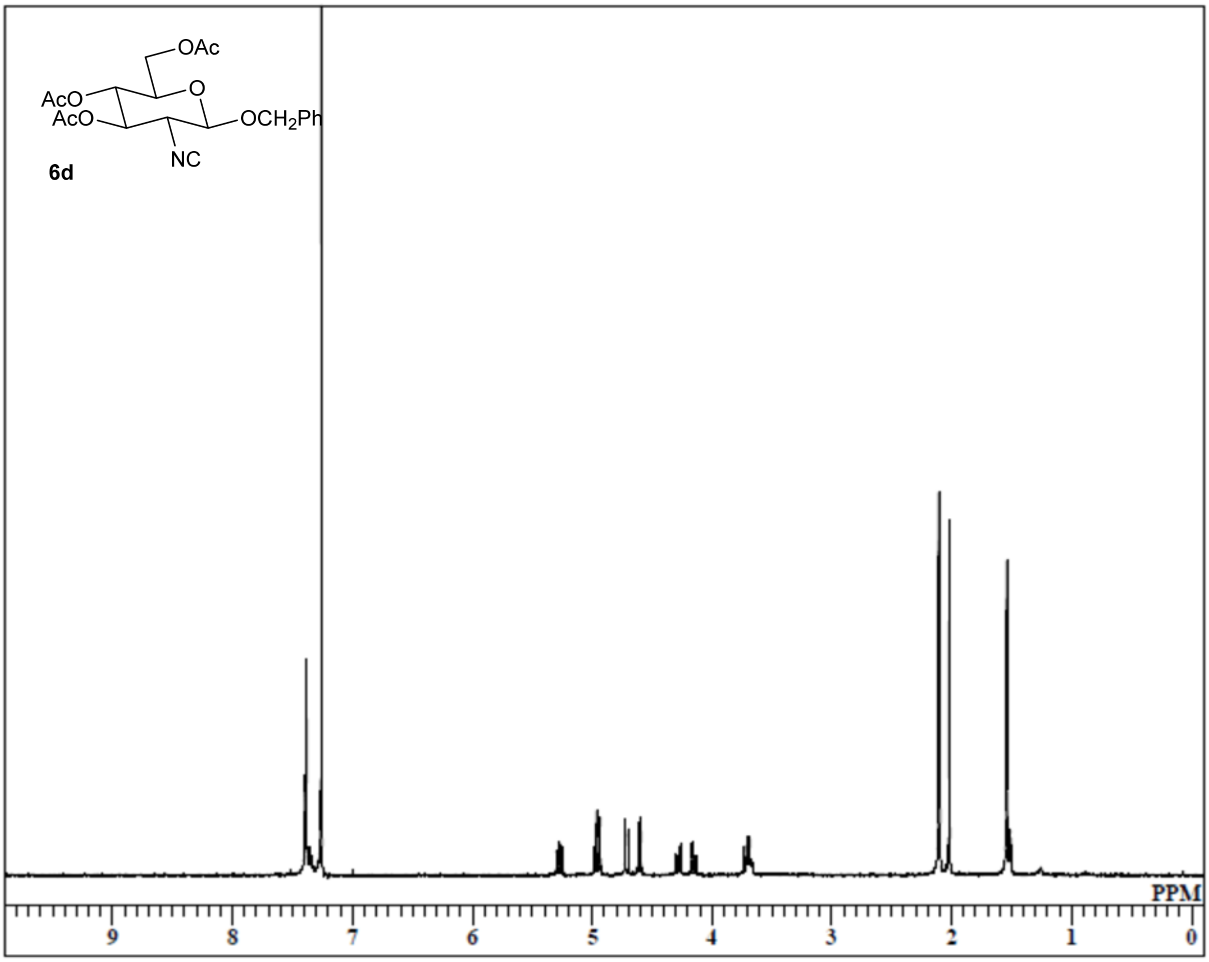
OAc
AcO
AcO
O
OCH2Ph
NC
6d
PPM
9
8
7
6
5
4
3
2
1
0

6d

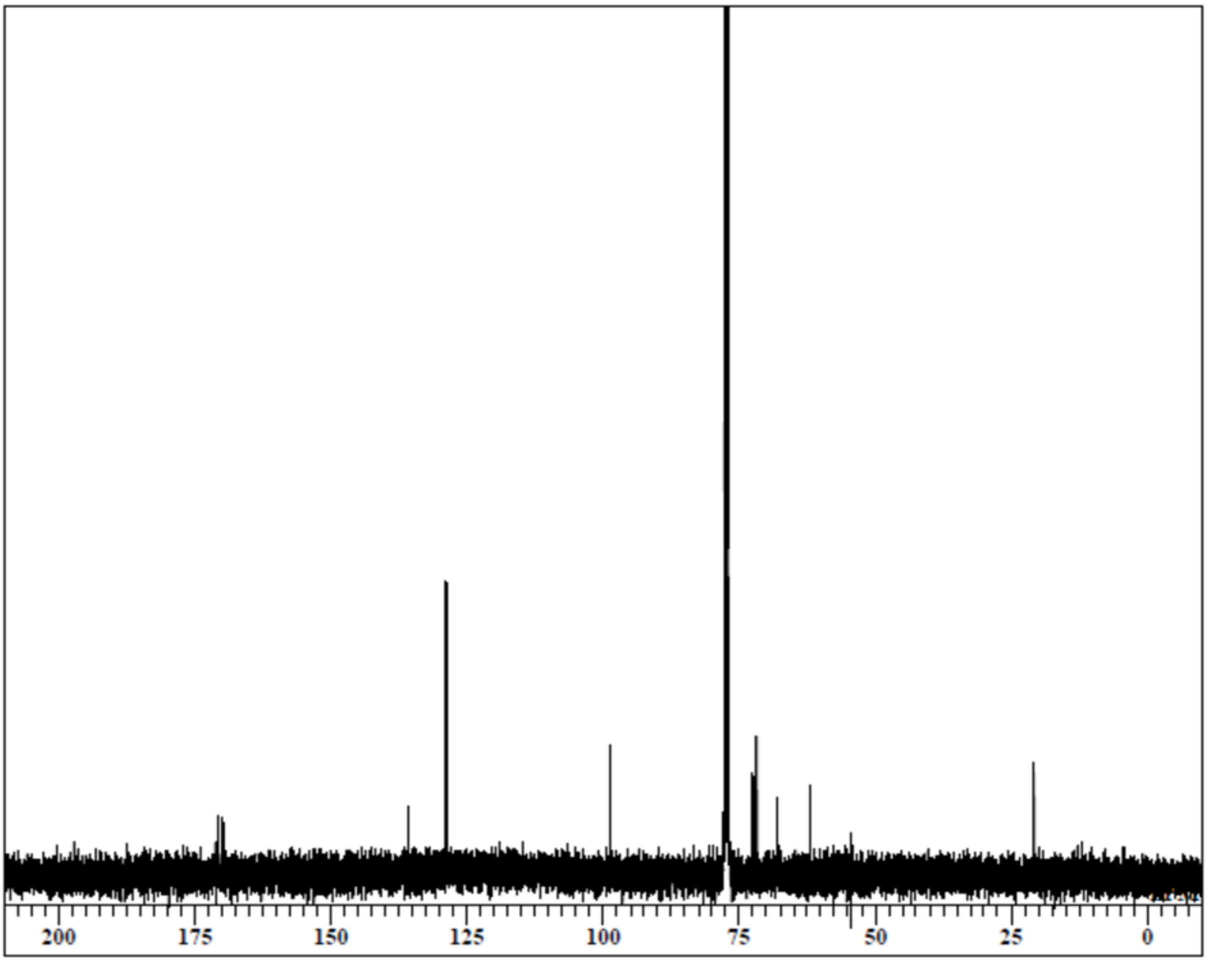
200
175
150
125
100
75
50
25
0

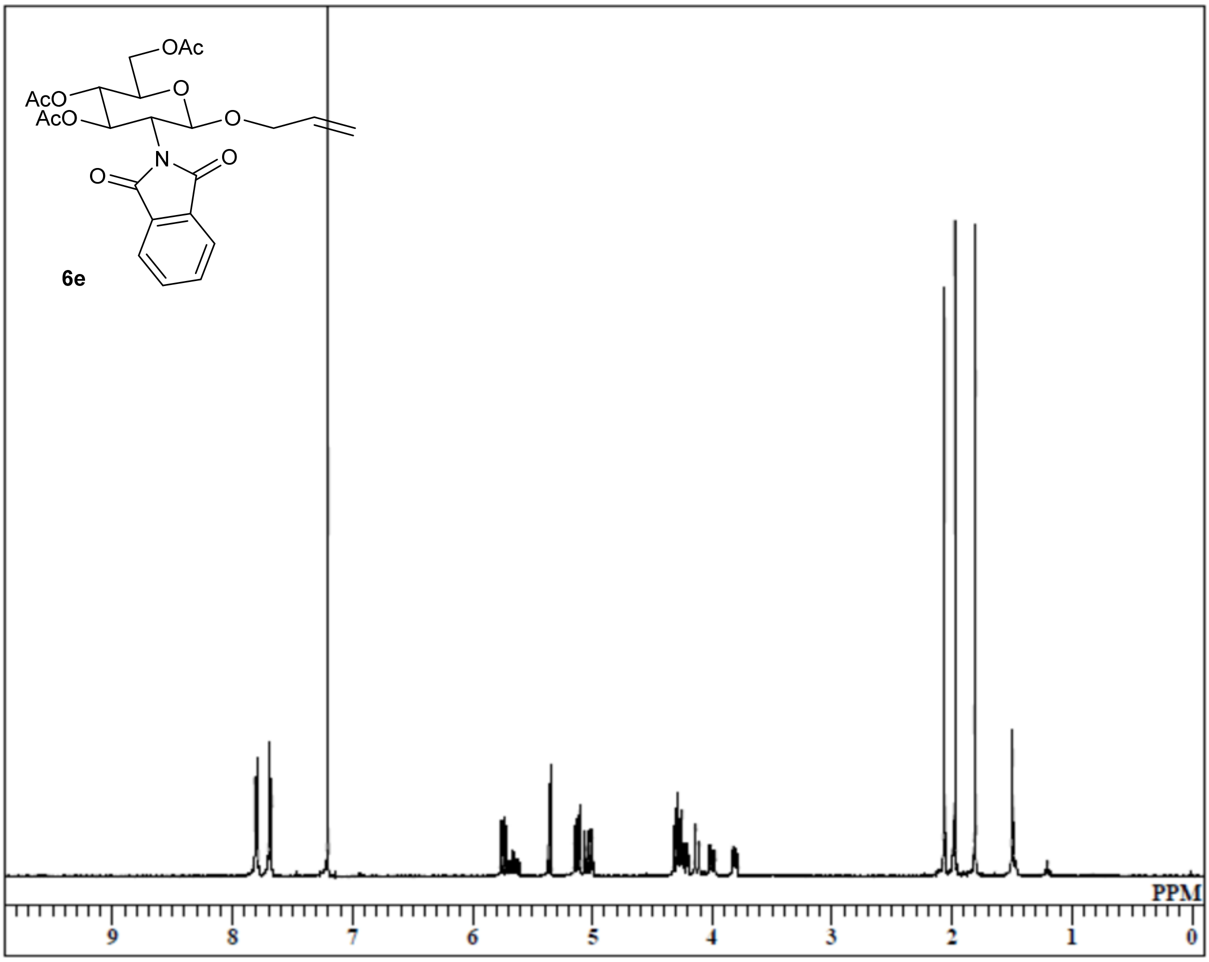
OAc
AcO
AcO
O
N
6e
PPM
9
8
7
6
5
4
3
2
1
0

9e

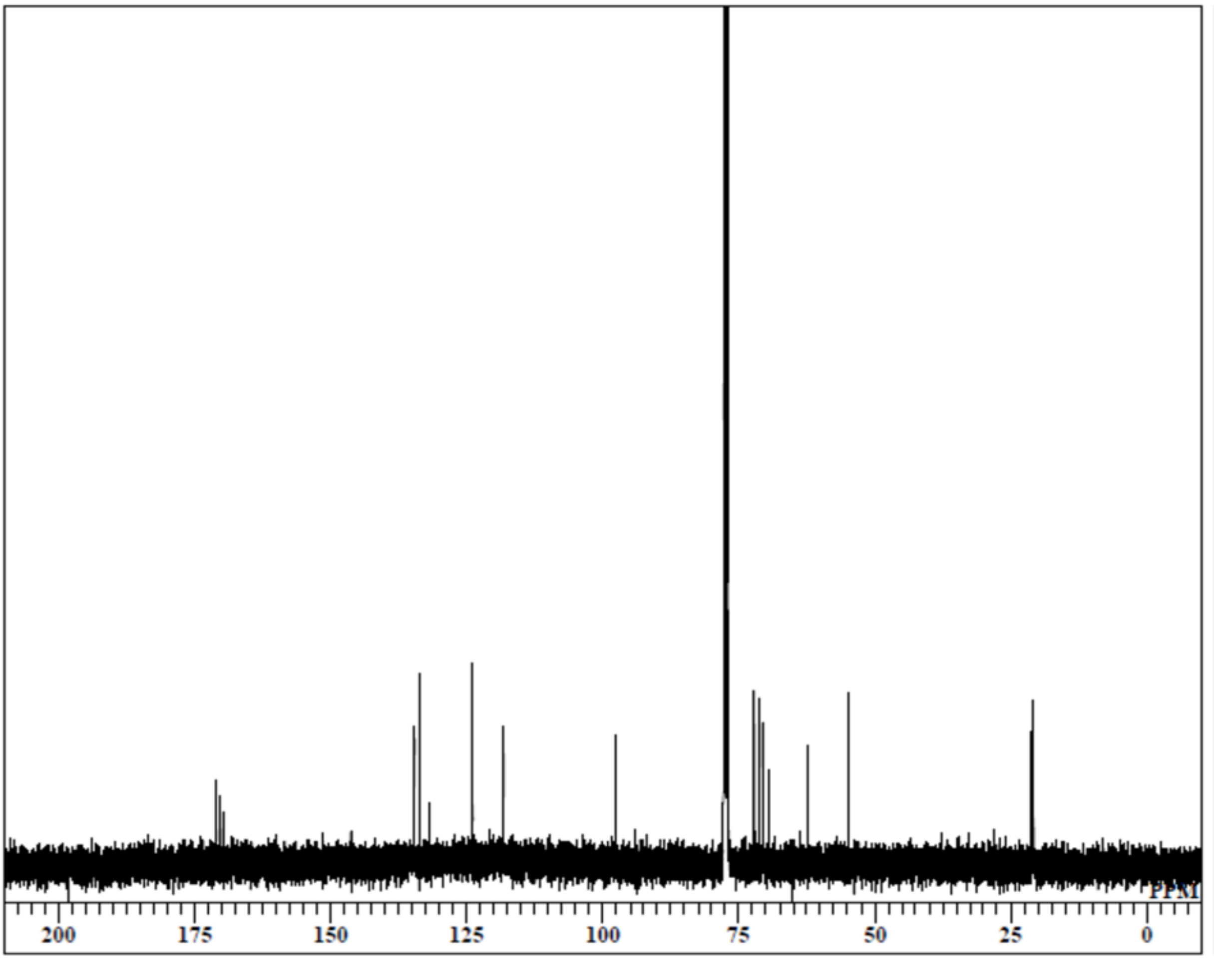
PPM
200
175
150
125
100
75
50
25
0

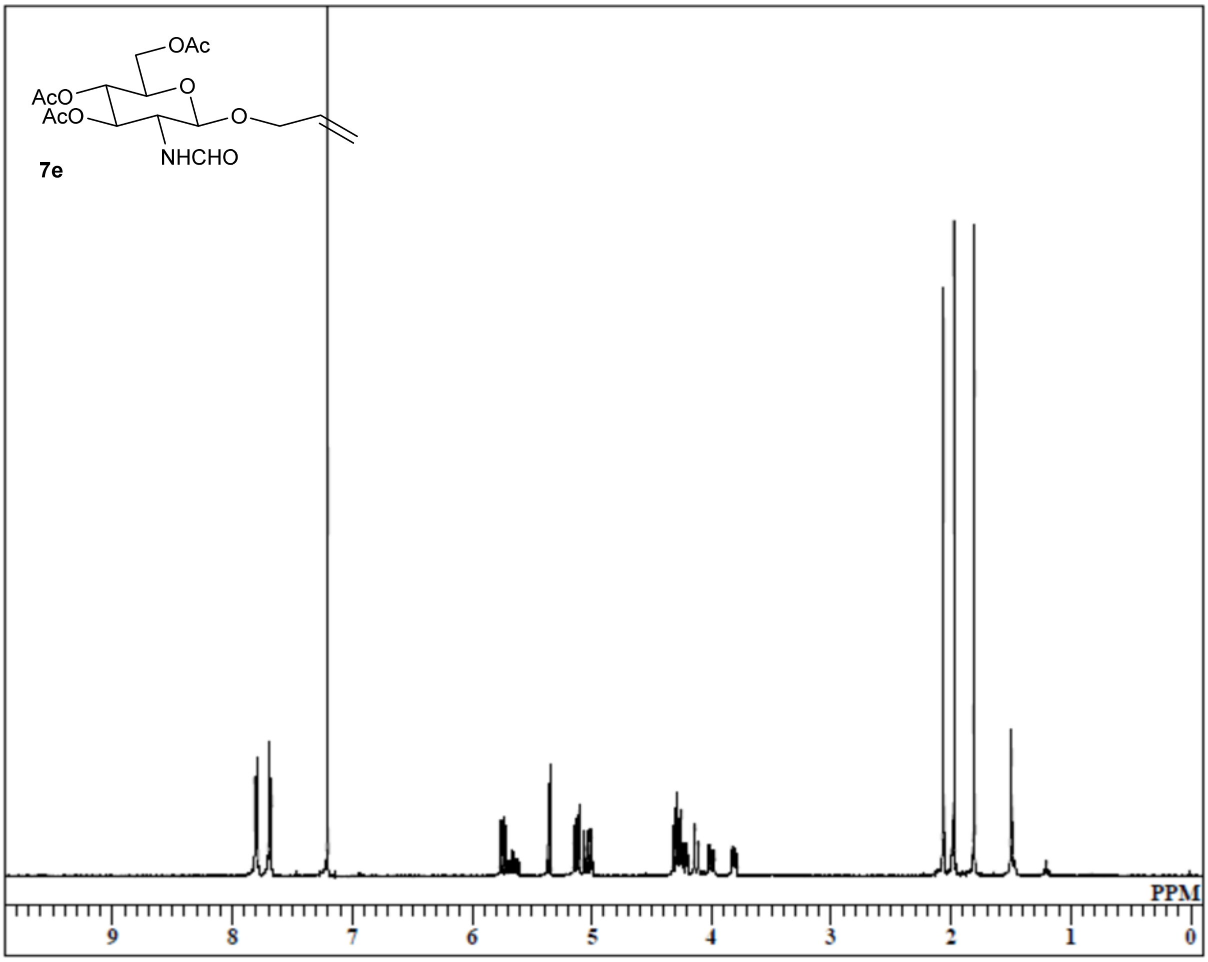
OAc
AcO
AcO
O
O
NHCHO
7e
PPM
9
8
7
6
5
4
3
2
1
0

7e

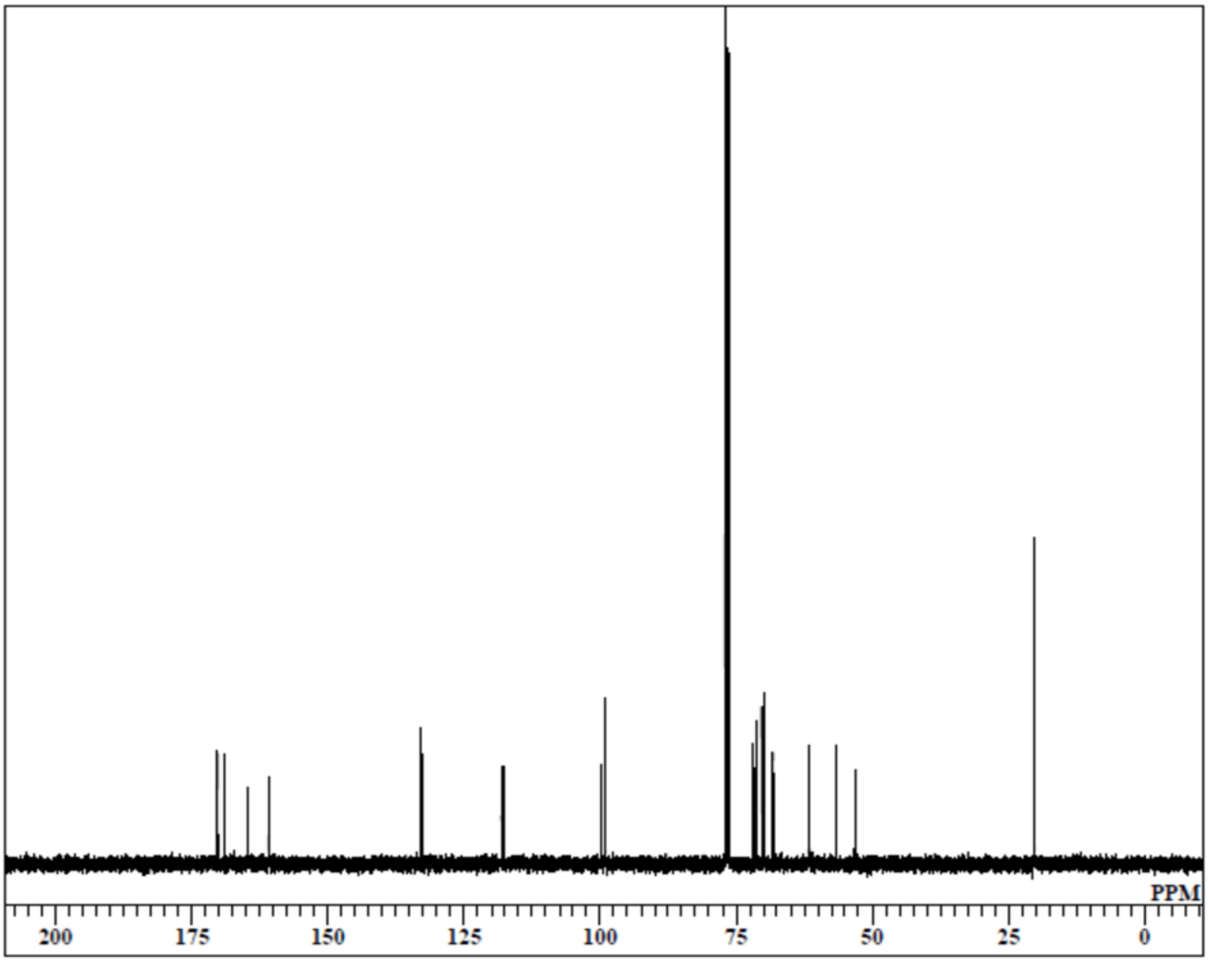
PPM
200
175
150
125
100
75
50
25
0

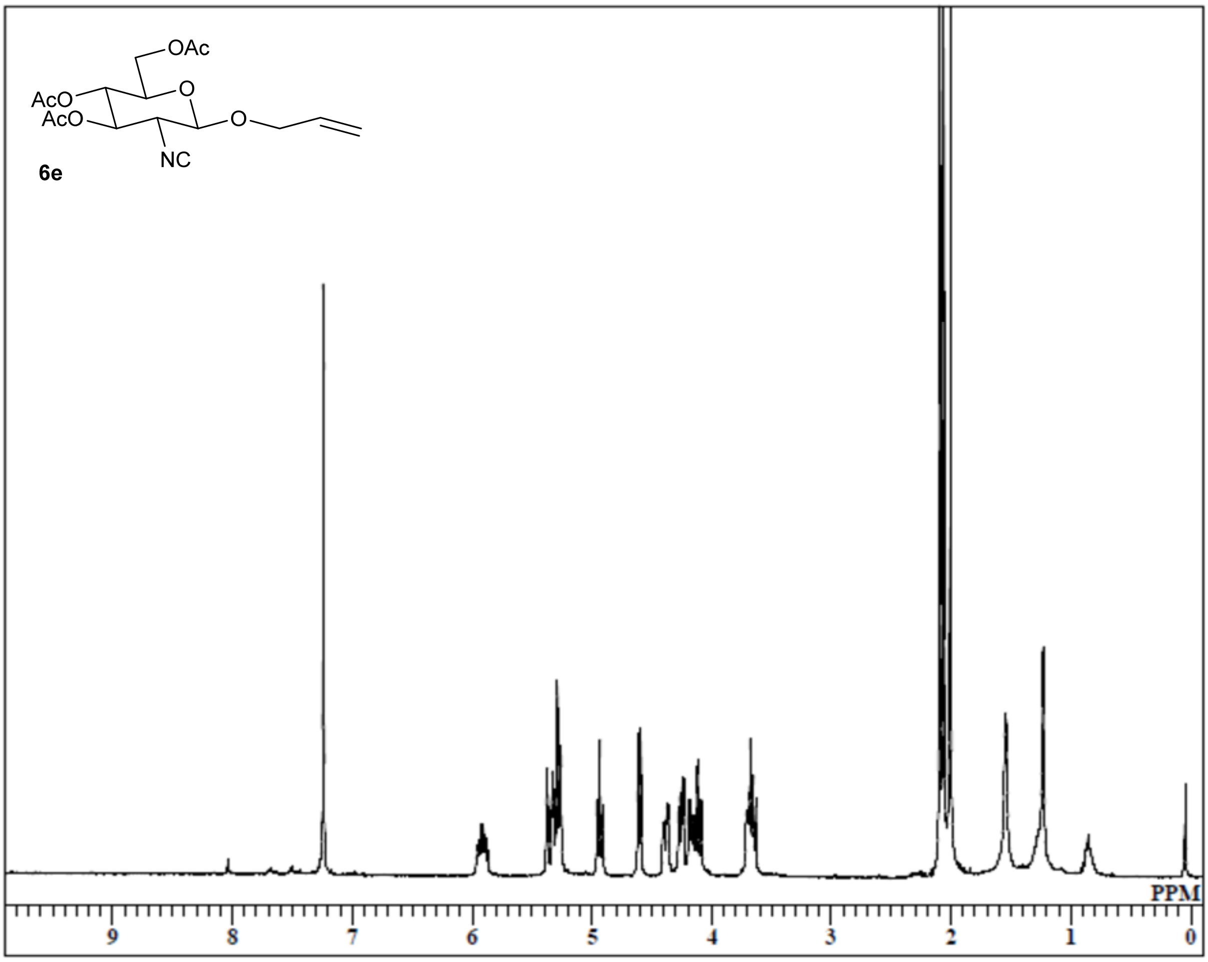
OAc
AcO
AcO
O
O
NC
6e
PPM
9
8
7
6
5
4
3
2
1
0

6e

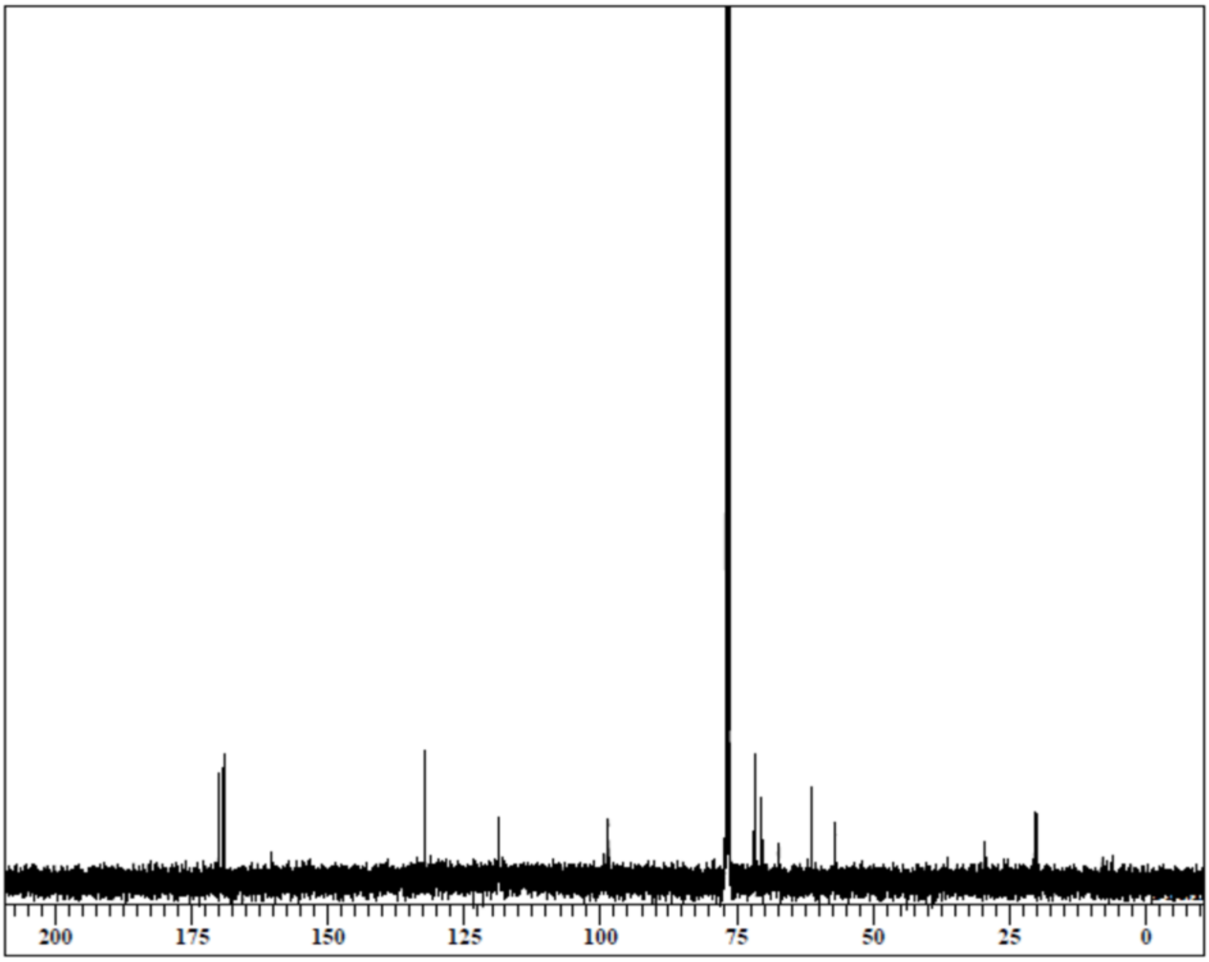
200
175
150
125
100
75
50
25
0

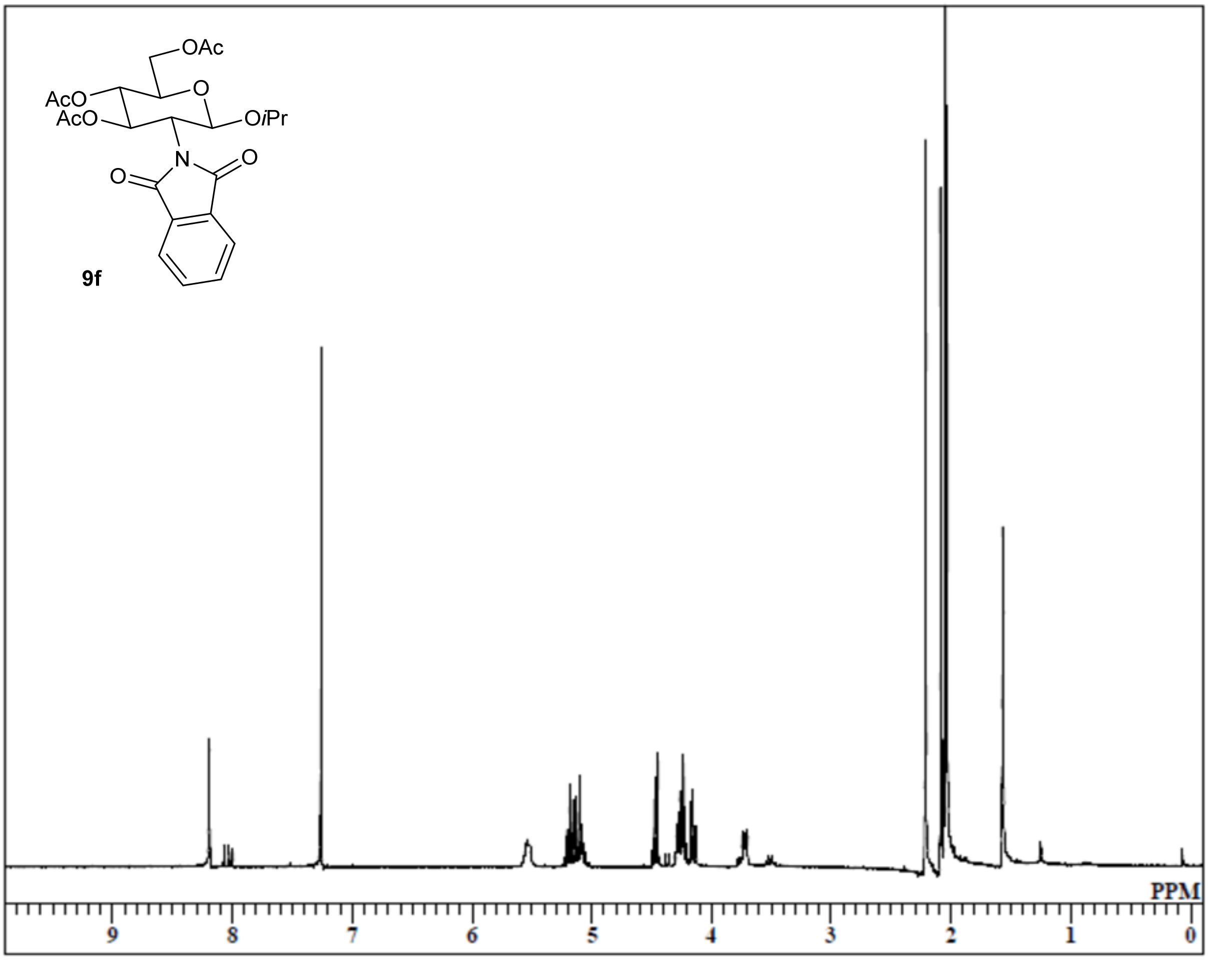
OAc
AcO
AcO
O
OiPr
N
O
O
9f
PPM
9
8
7
6
5
4
3
2
1
0
9f

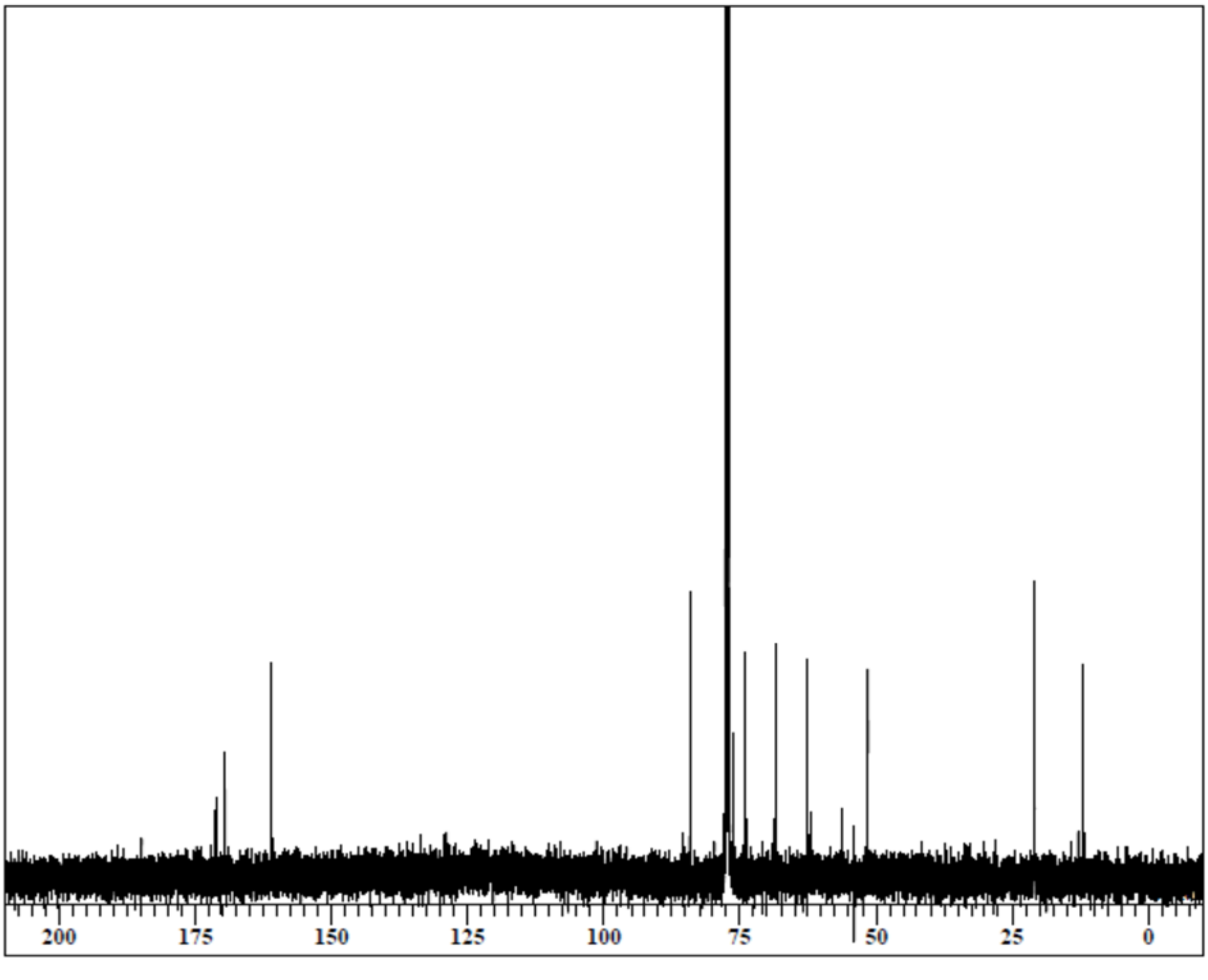
200
175
150
125
100
75
50
25
0

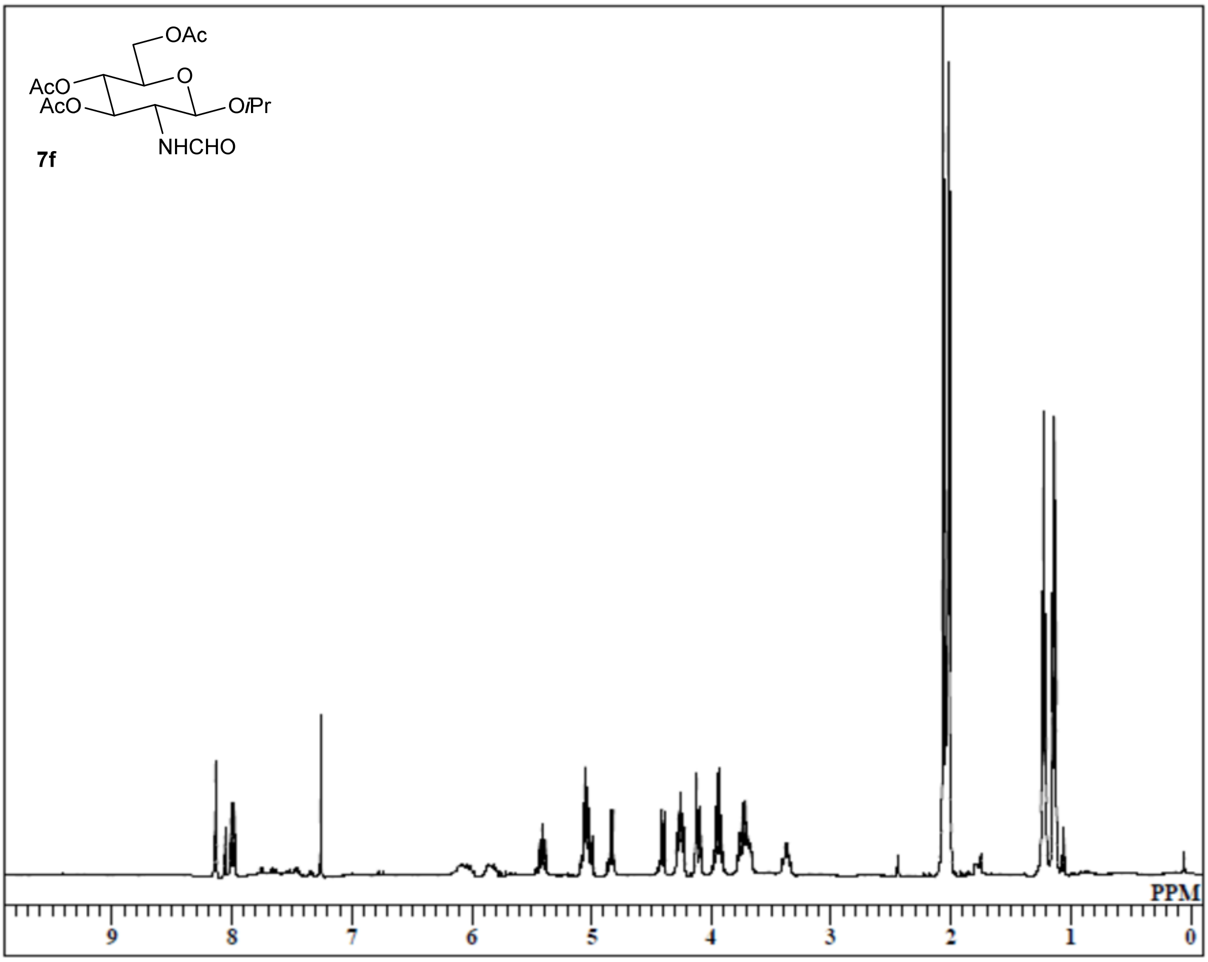
OAc
AcO
AcO
O
O*i*Pr
NHCHO
7f
PPM
9 8 7 6 5 4 3 2 1 0

7f

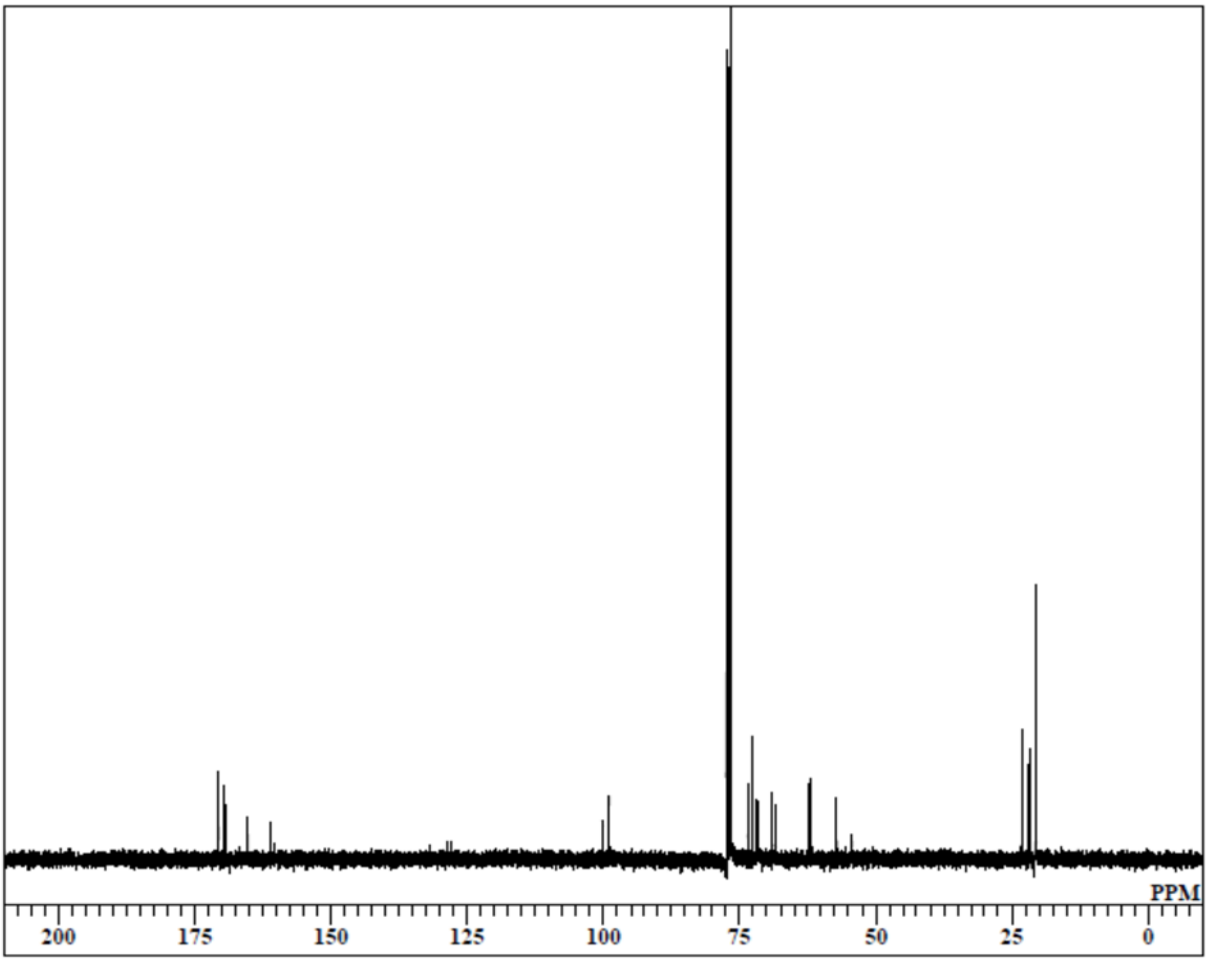
PPM
200 175 150 125 100 75 50 25 0

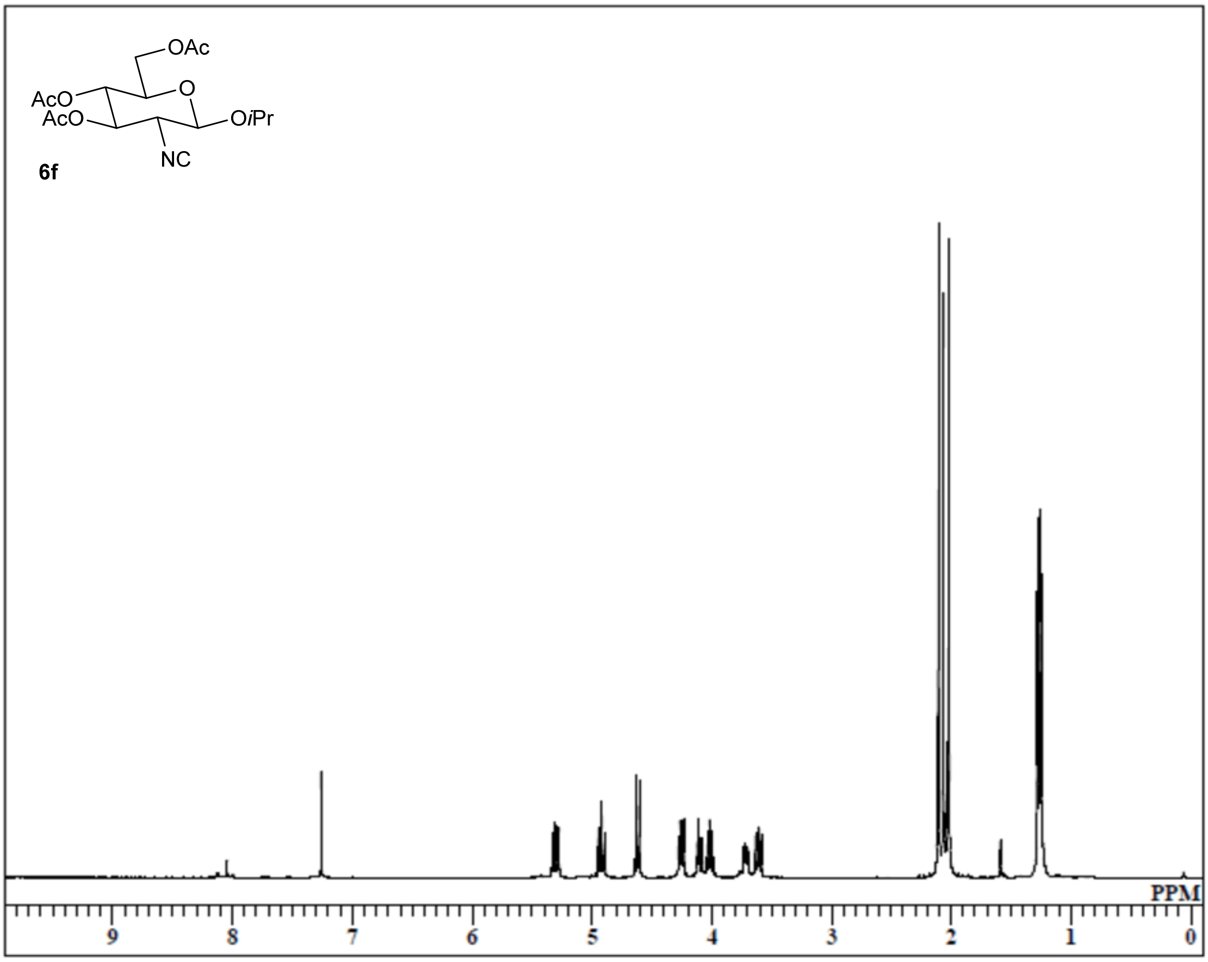
OAc
AcO
AcO
O
OiPr
NC
6f
PPM
9
8
7
6
5
4
3
2
1
0
6f

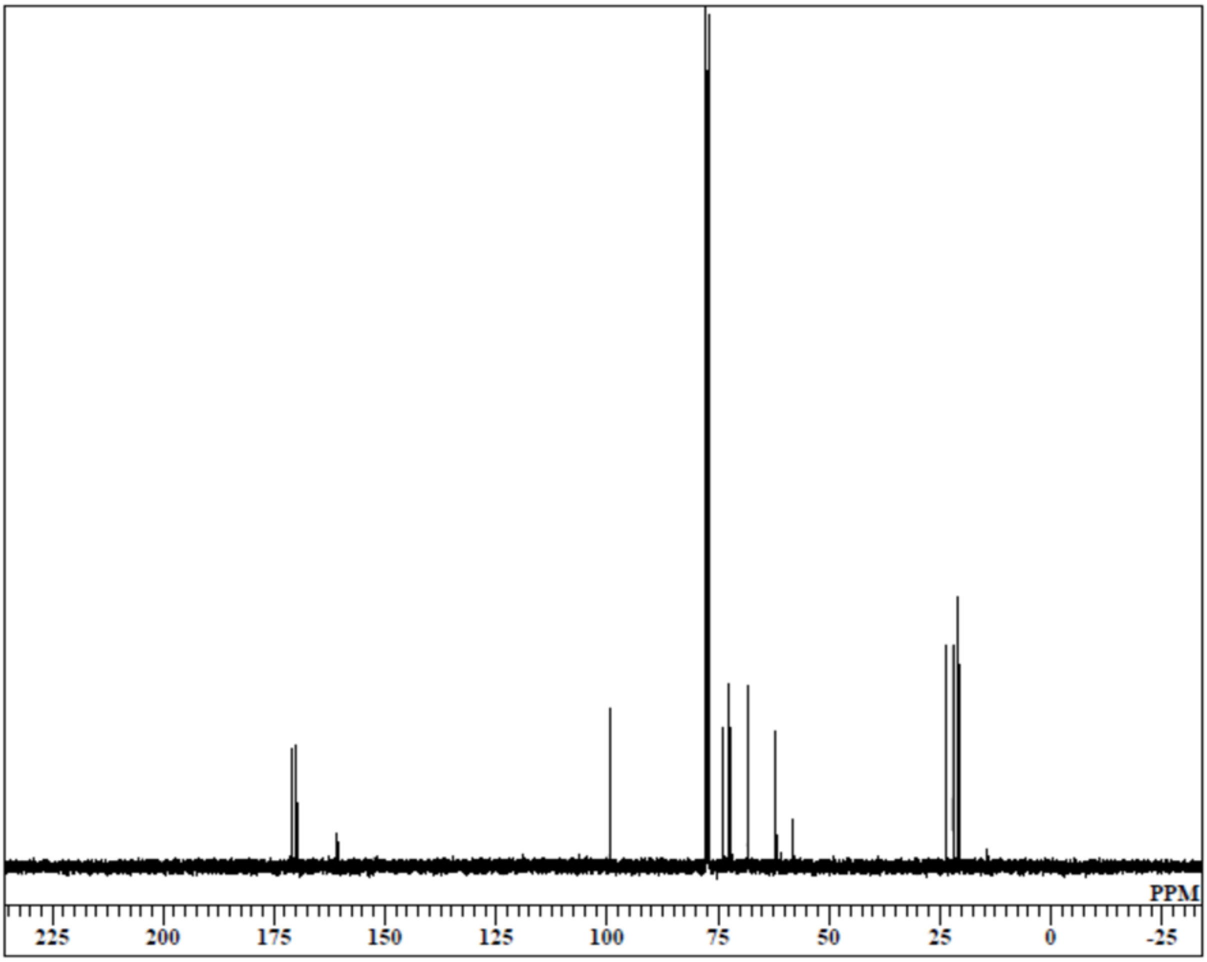
PPM
225
200
175
150
125
100
75
50
25
0
-25

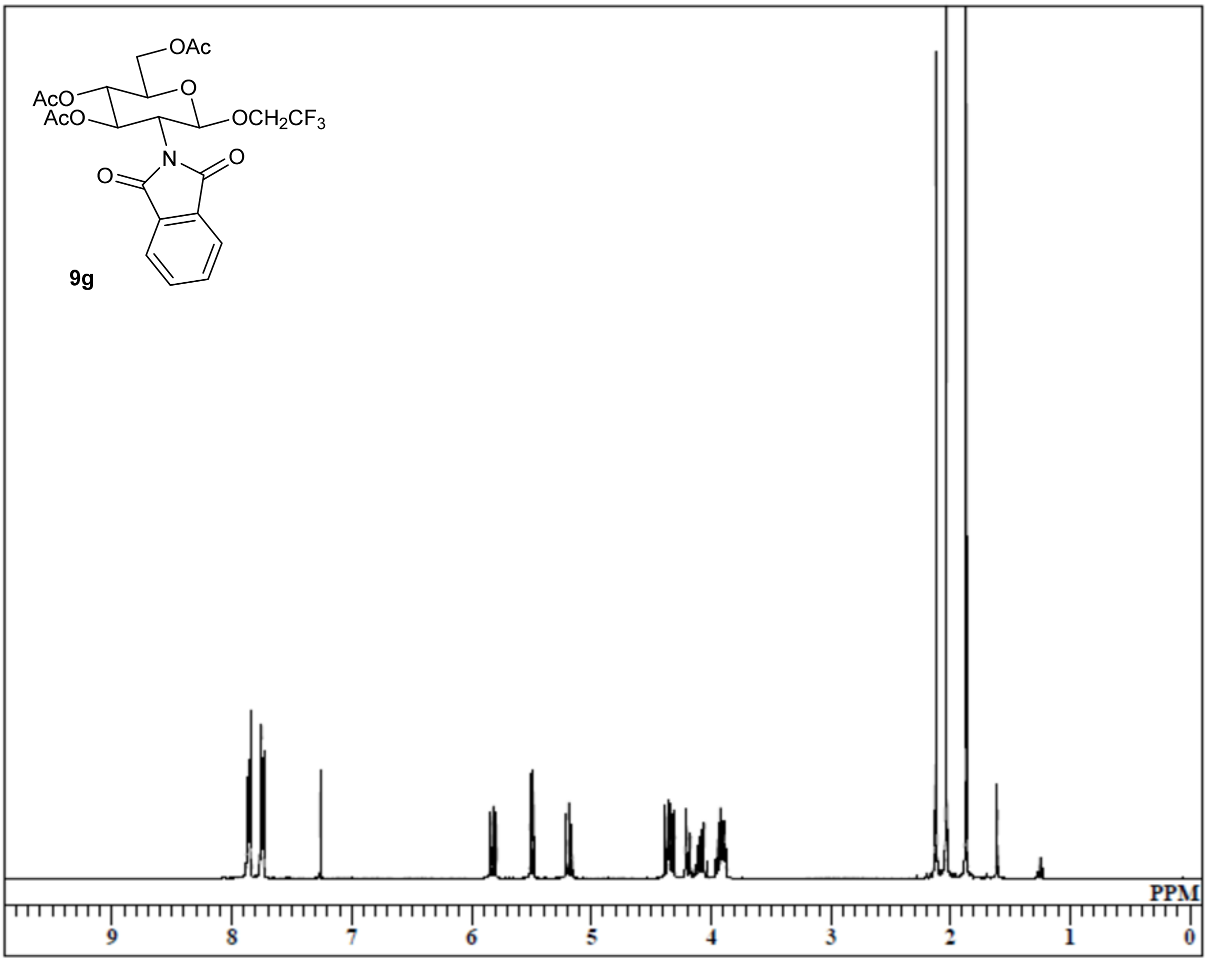
OAc
AcO
AcO
O
OCH2CF3
N
O
O
9g
PPM
9
8
7
6
5
4
3
2
1
0

9g

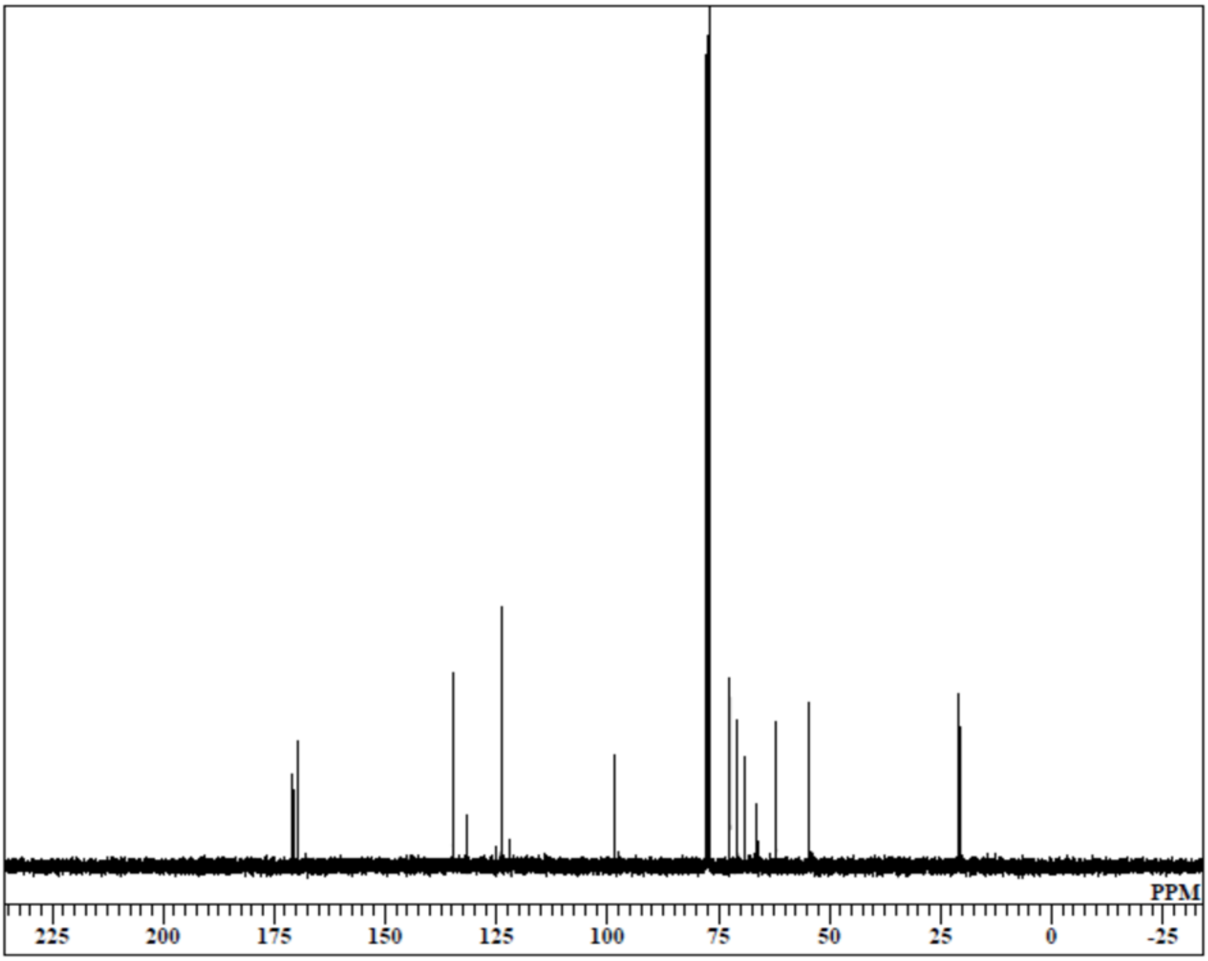
PPM
225
200
175
150
125
100
75
50
25
0
-25

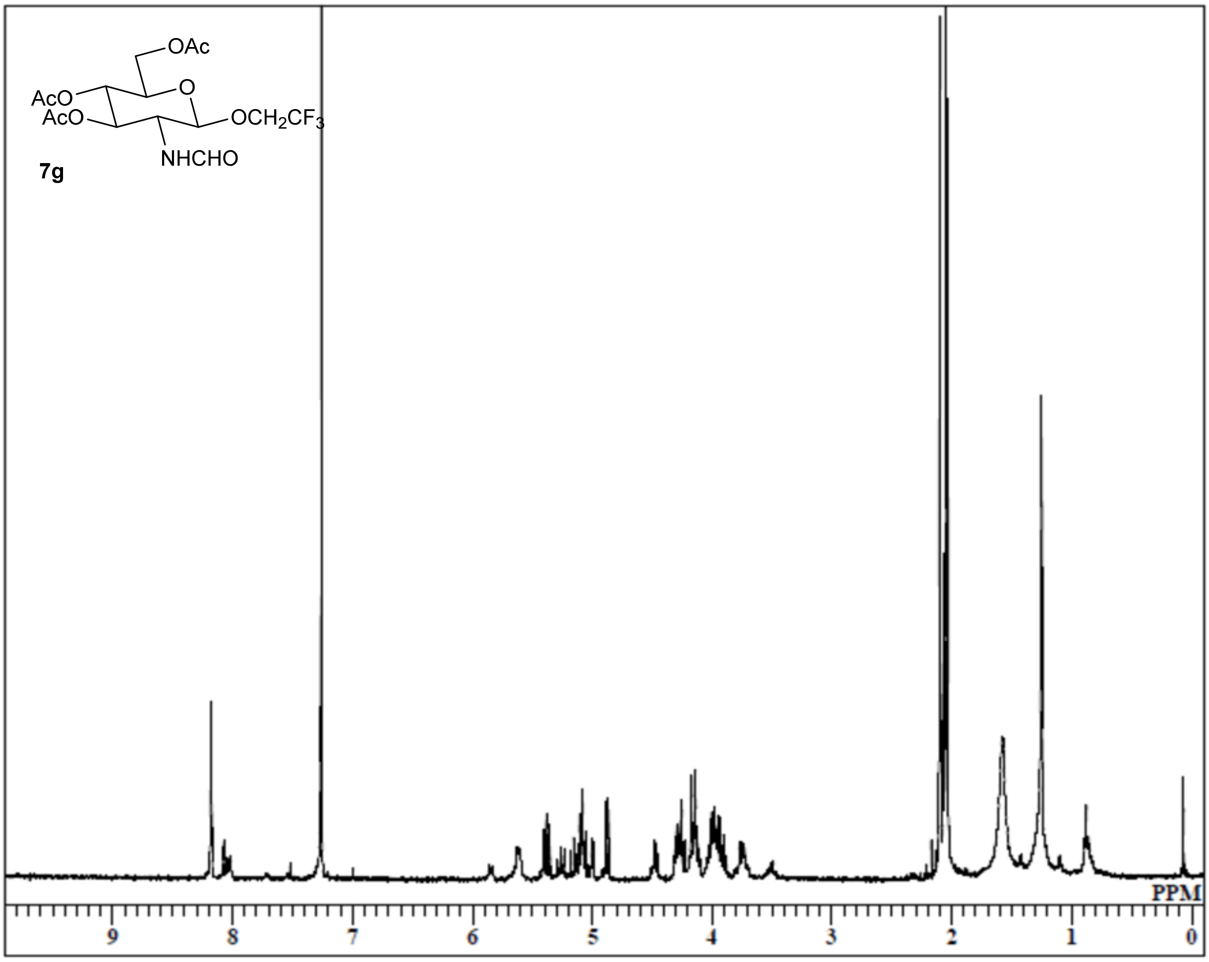
OAc
AcO
AcO
O
OCH2CF3
NHCHO
7g
PPM
9
8
7
6
5
4
3
2
1
0

7g

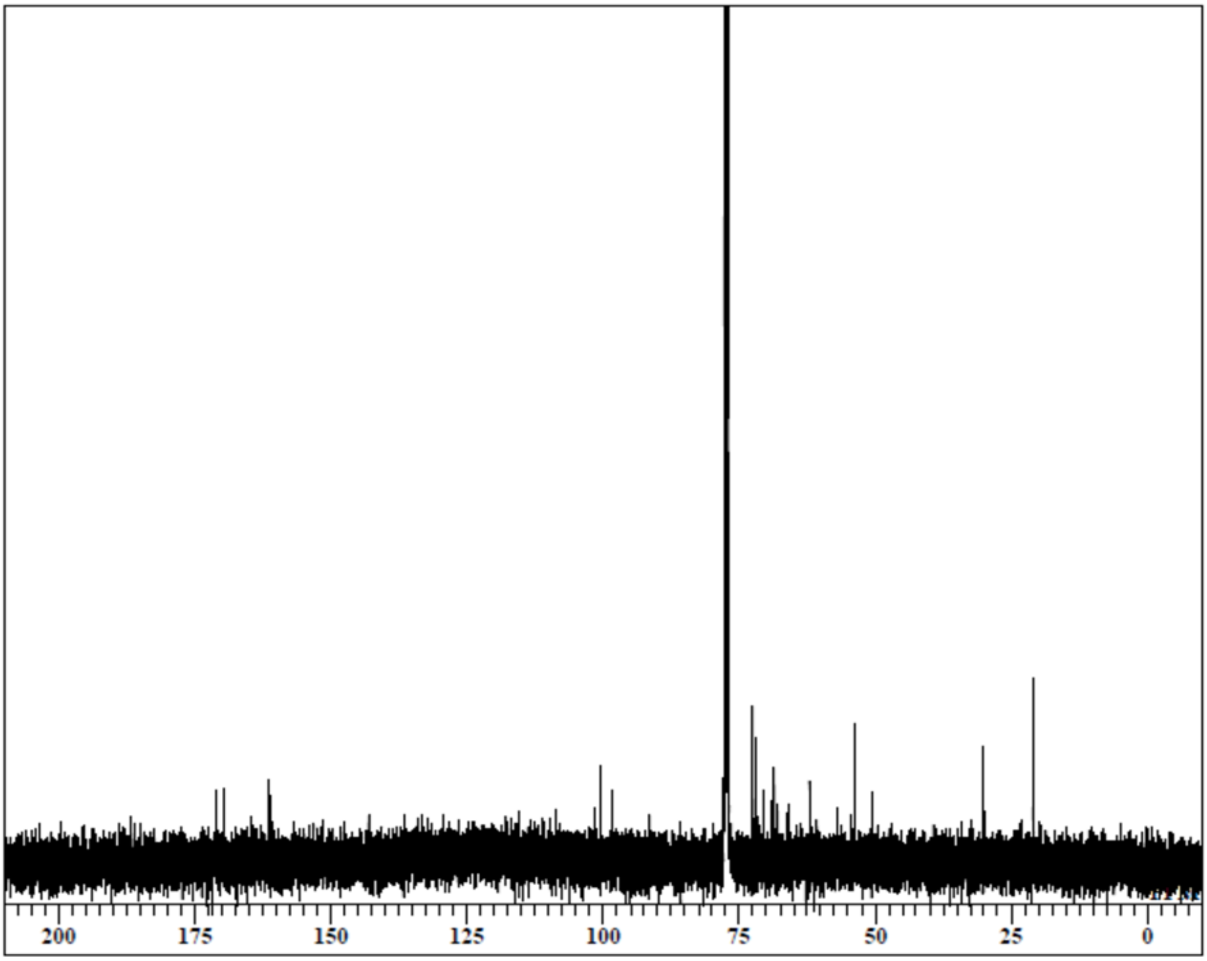
200
175
150
125
100
75
50
25
0

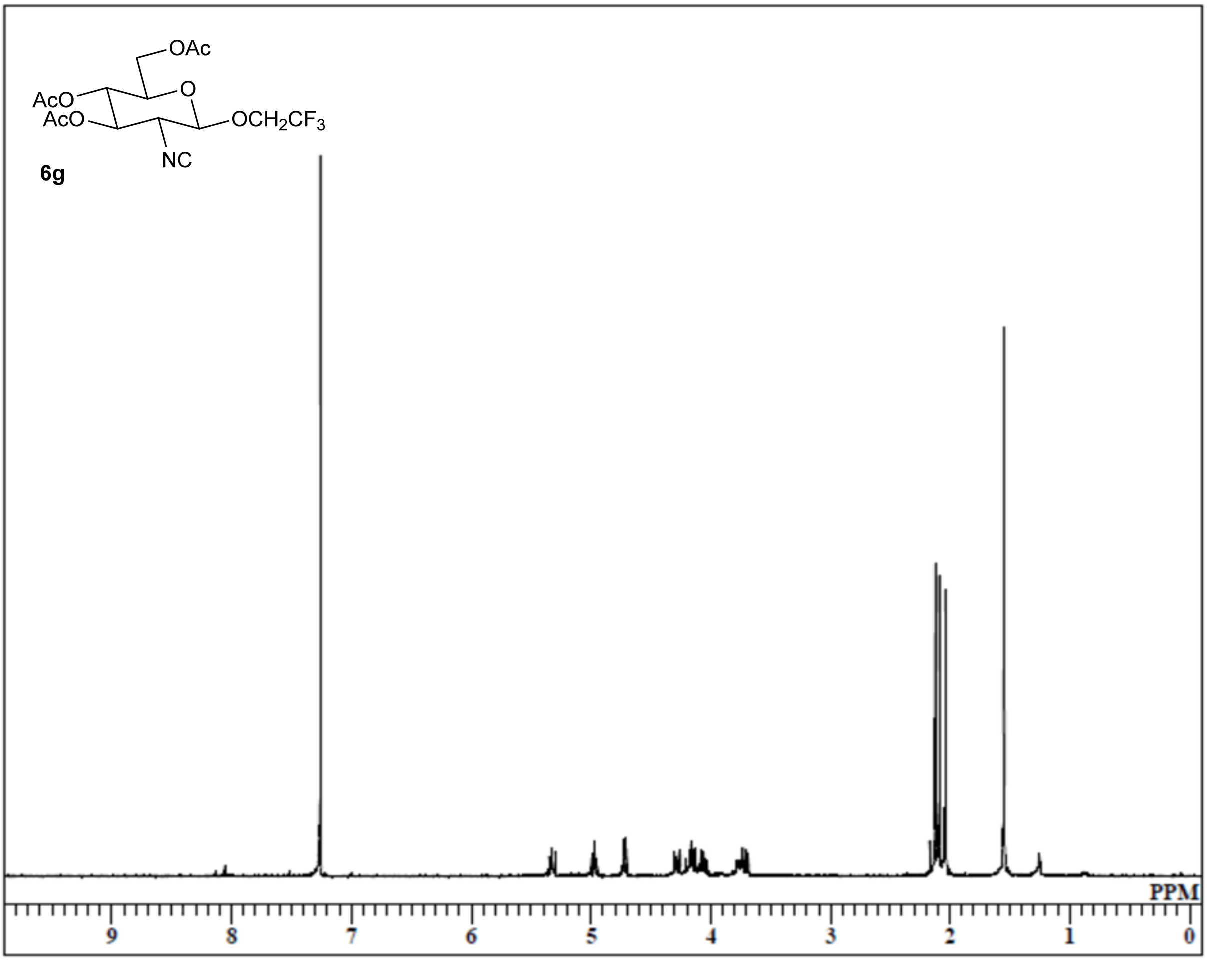
OAc
AcO
AcO
O
OCH2CF3
NC
6g
PPM
9
8
7
6
5
4
3
2
1
0

6g

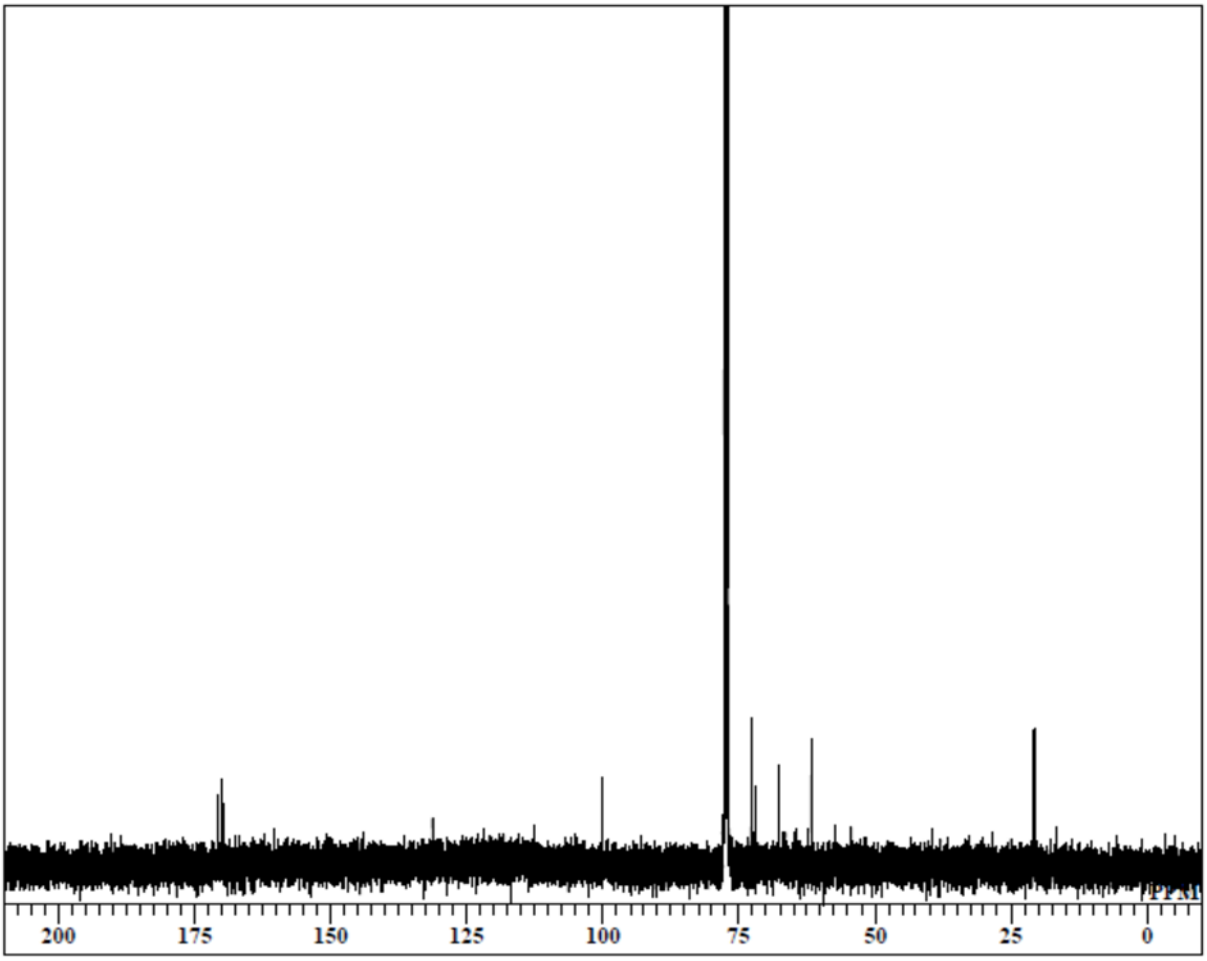
PPM
200
175
150
125
100
75
50
25
0

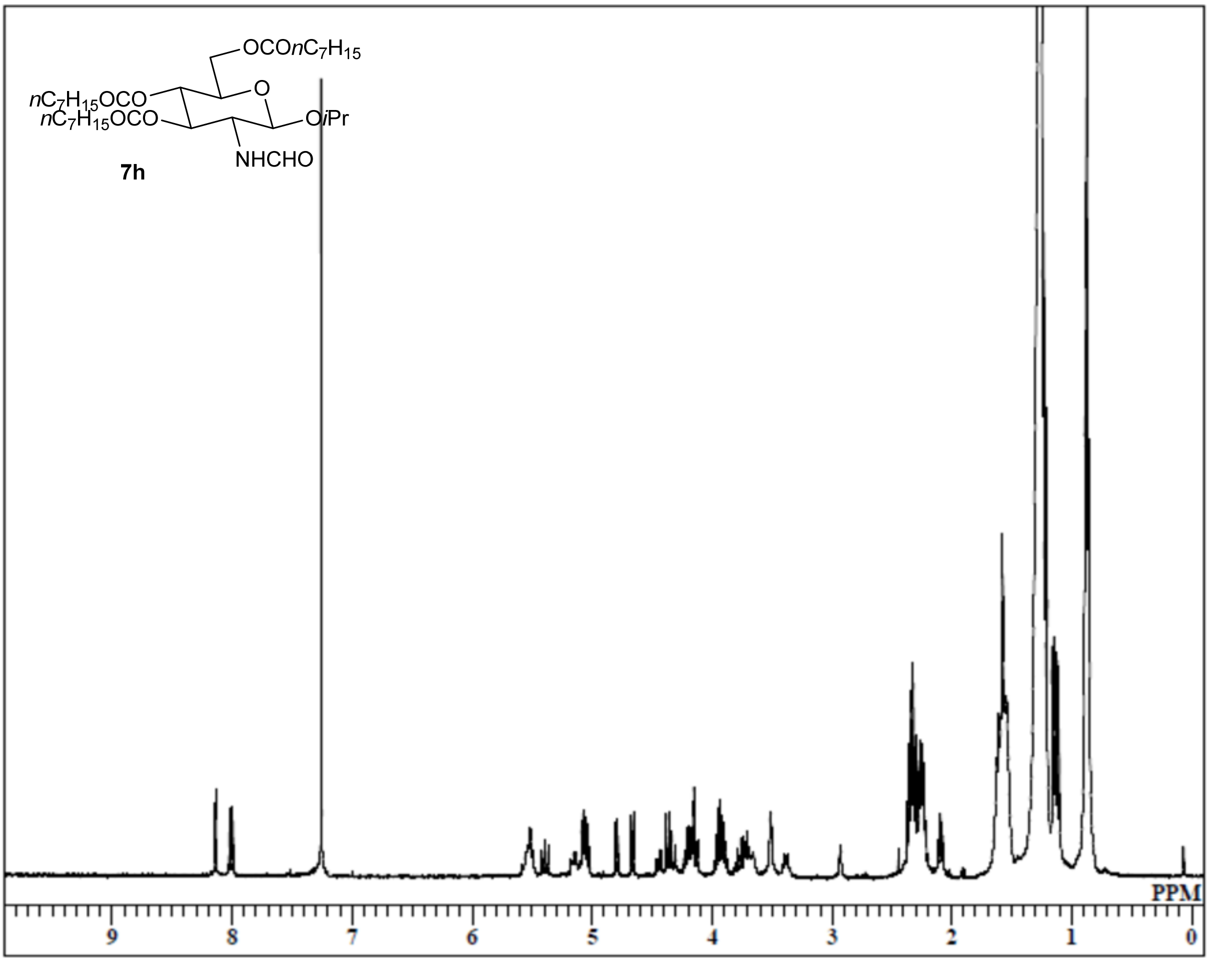
OCOnC7H15
nC7H15OCO
nC7H15OCO
O
OiPr
NHCHO
7h
PPM
9
8
7
6
5
4
3
2
1
0
7h

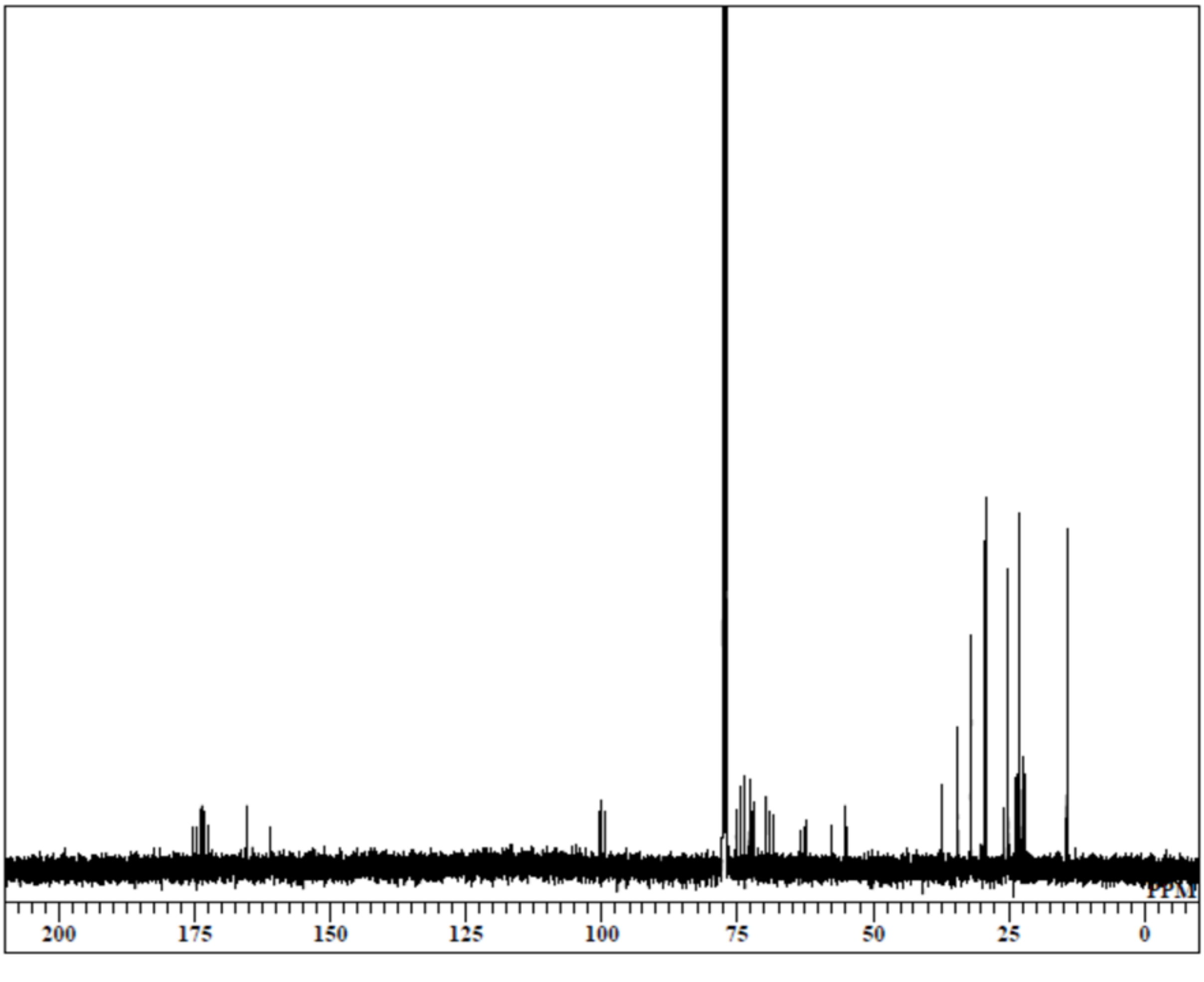
PPM
200
175
150
125
100
75
50
25
0

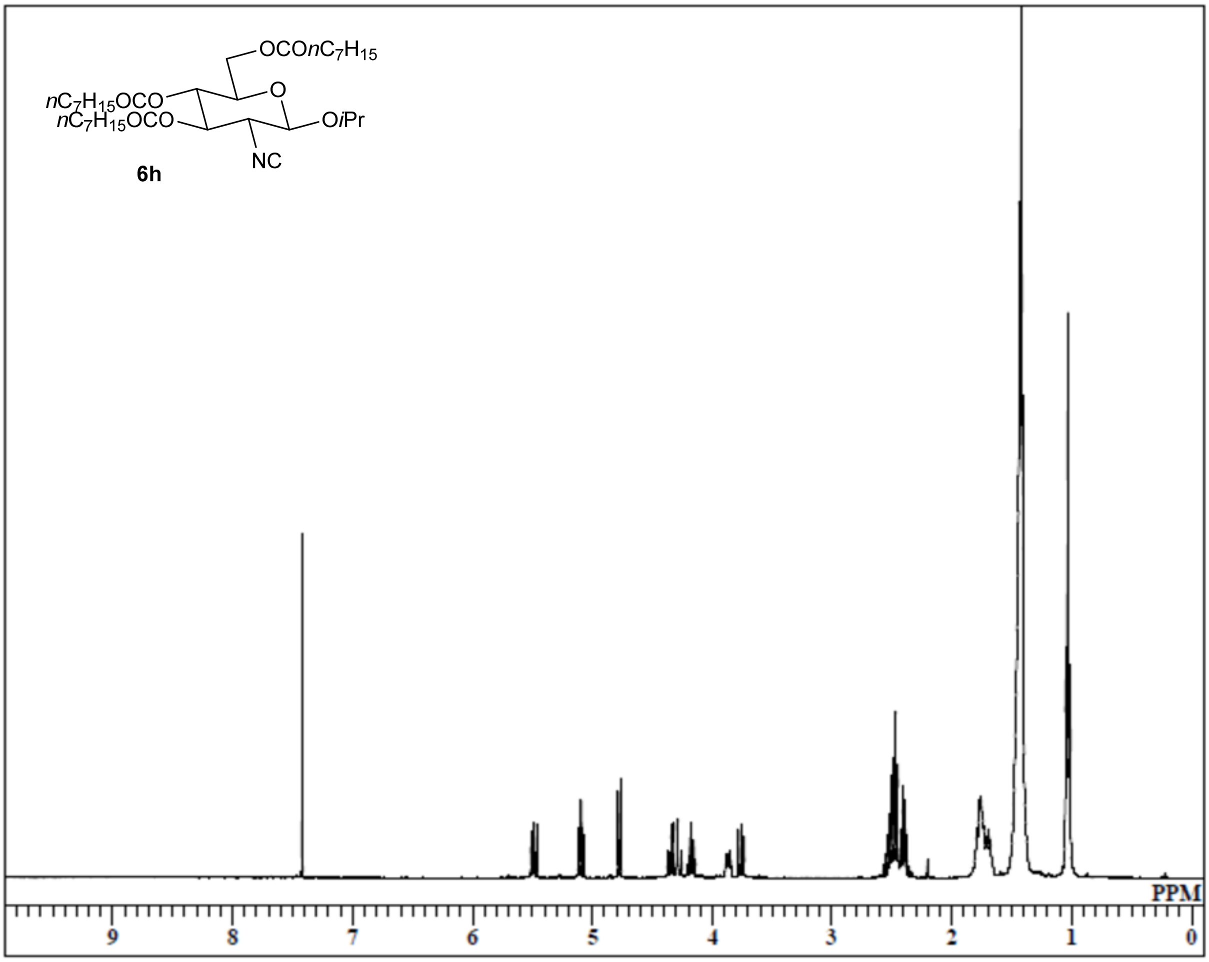
OCOnC7H15
nC7H15OCO
nC7H15OCO
O
OiPr
NC
6h
PPM
9
8
7
6
5
4
3
2
1
0

6h

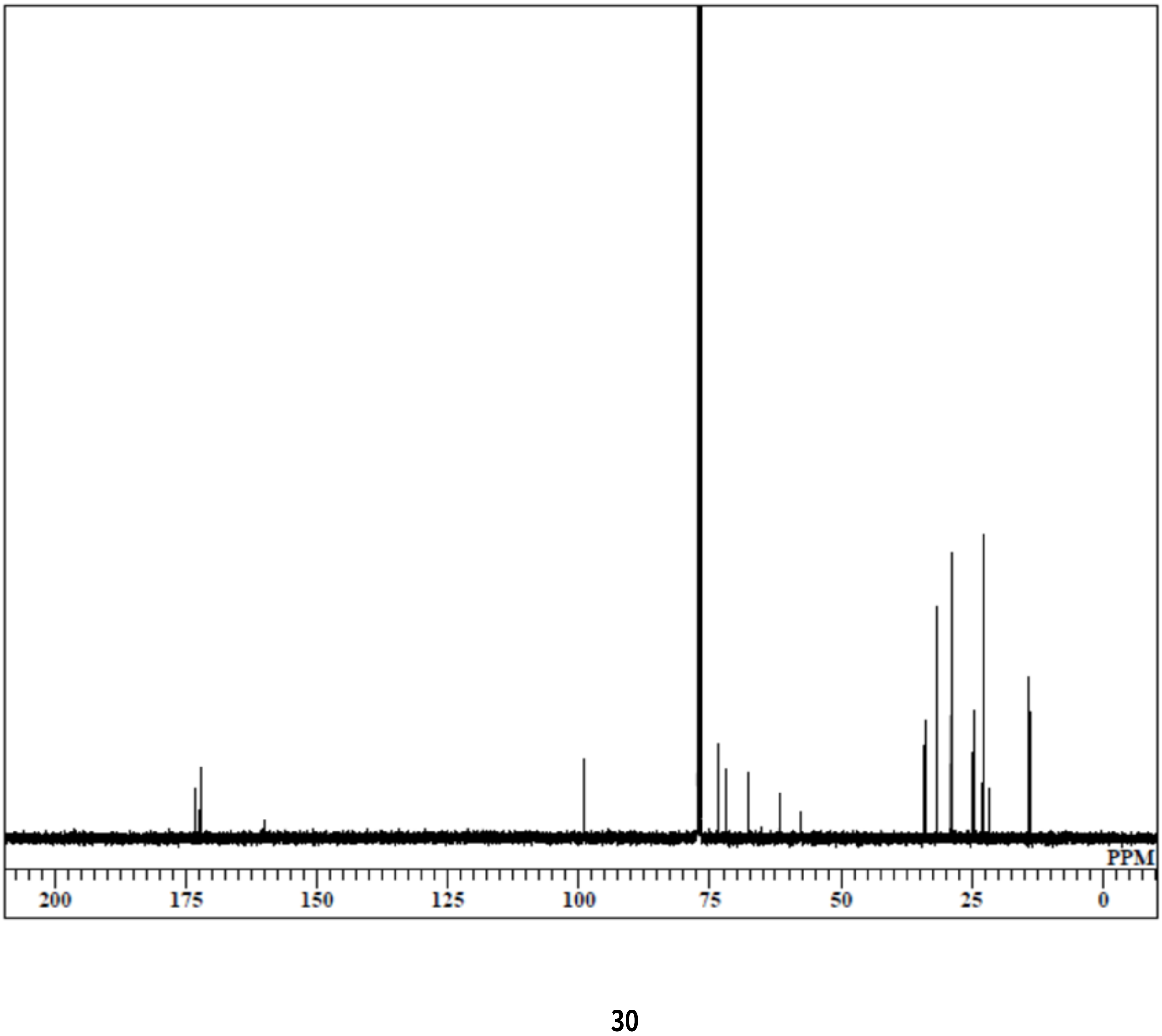
PPM
200
175
150
125
100
75
50
25
0

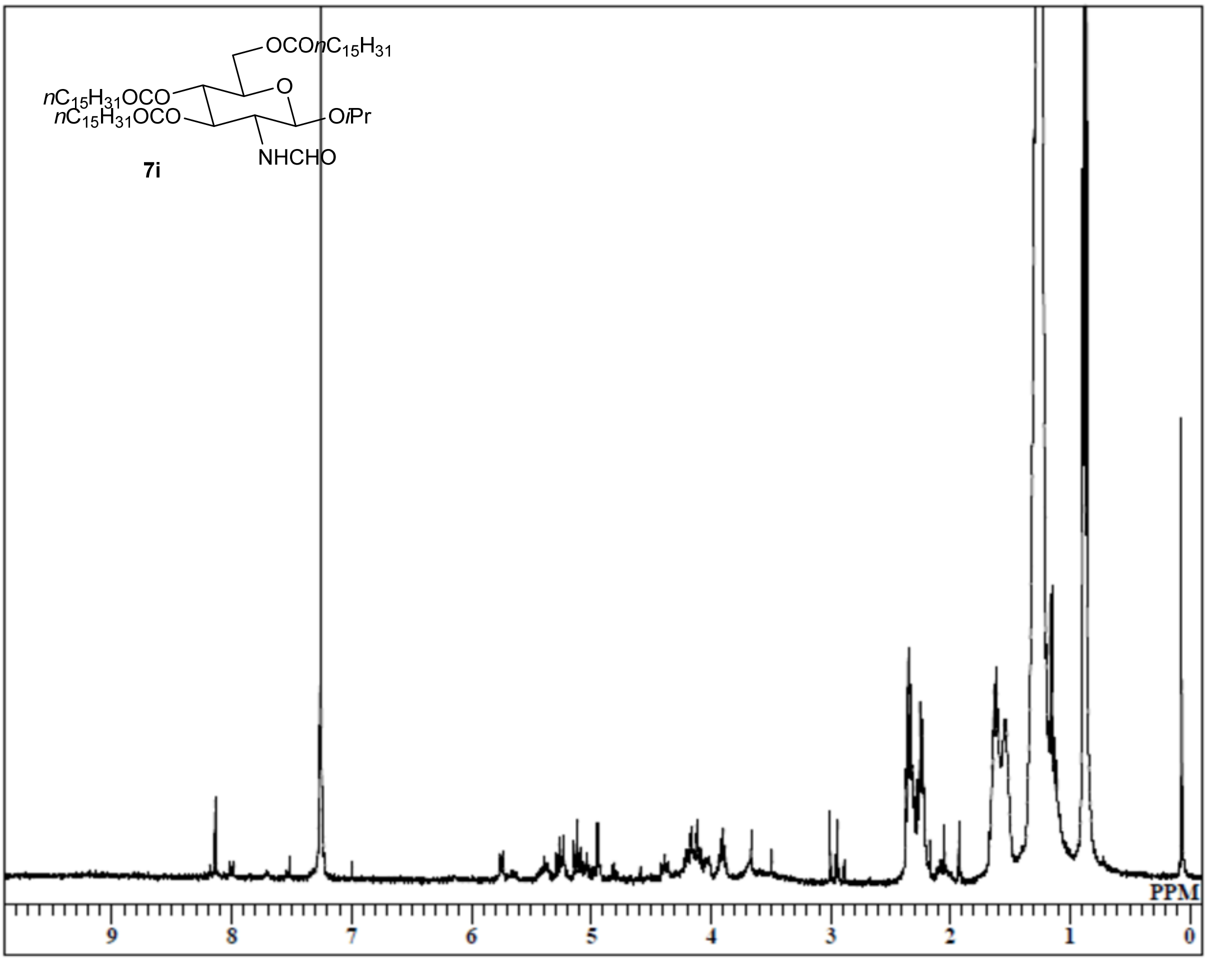
OCOnC15H31
nC15H31OCO
nC15H31OCO
O
OiPr
NHCHO
7i
PPM
9
8
7
6
5
4
3
2
1
0

7i

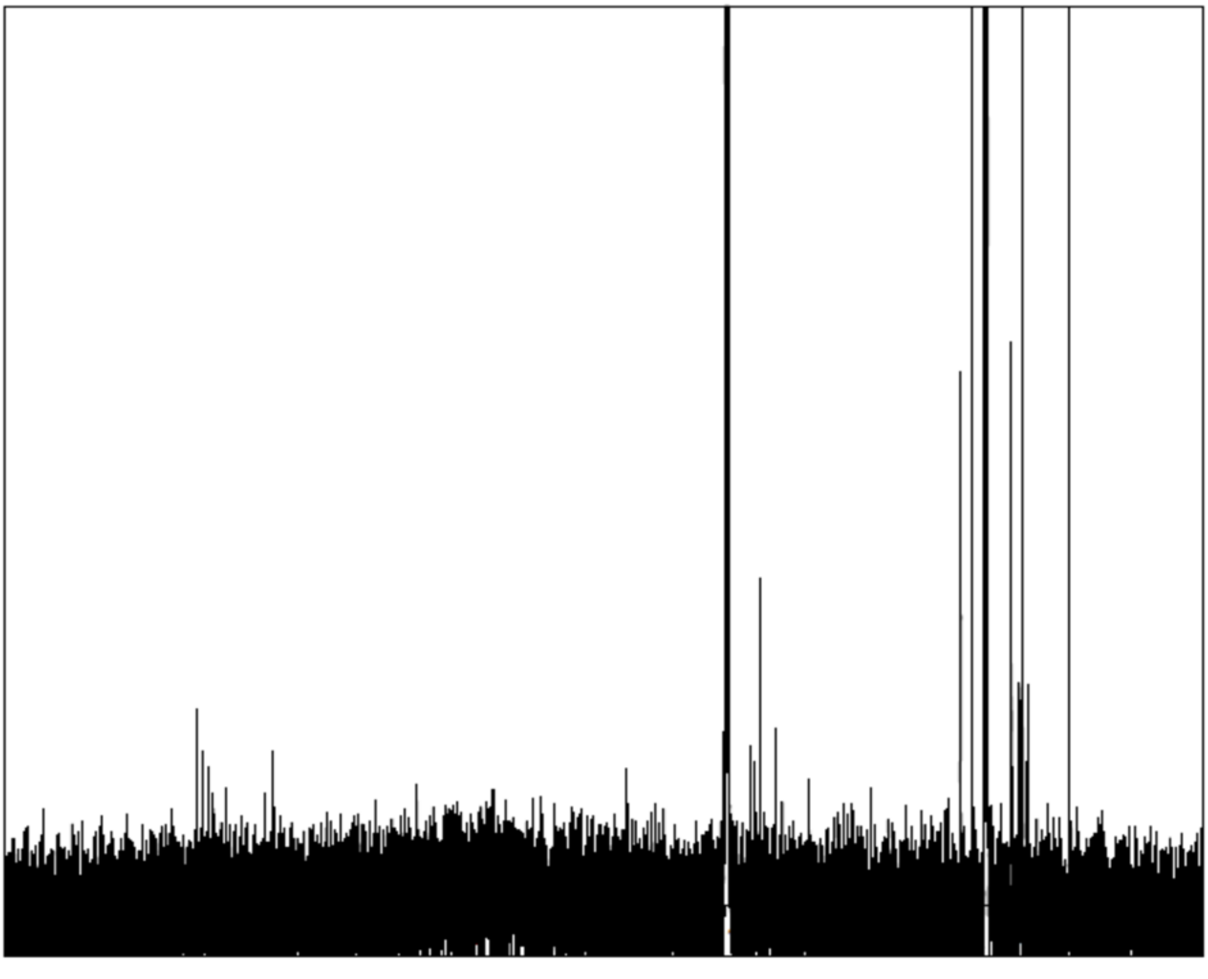

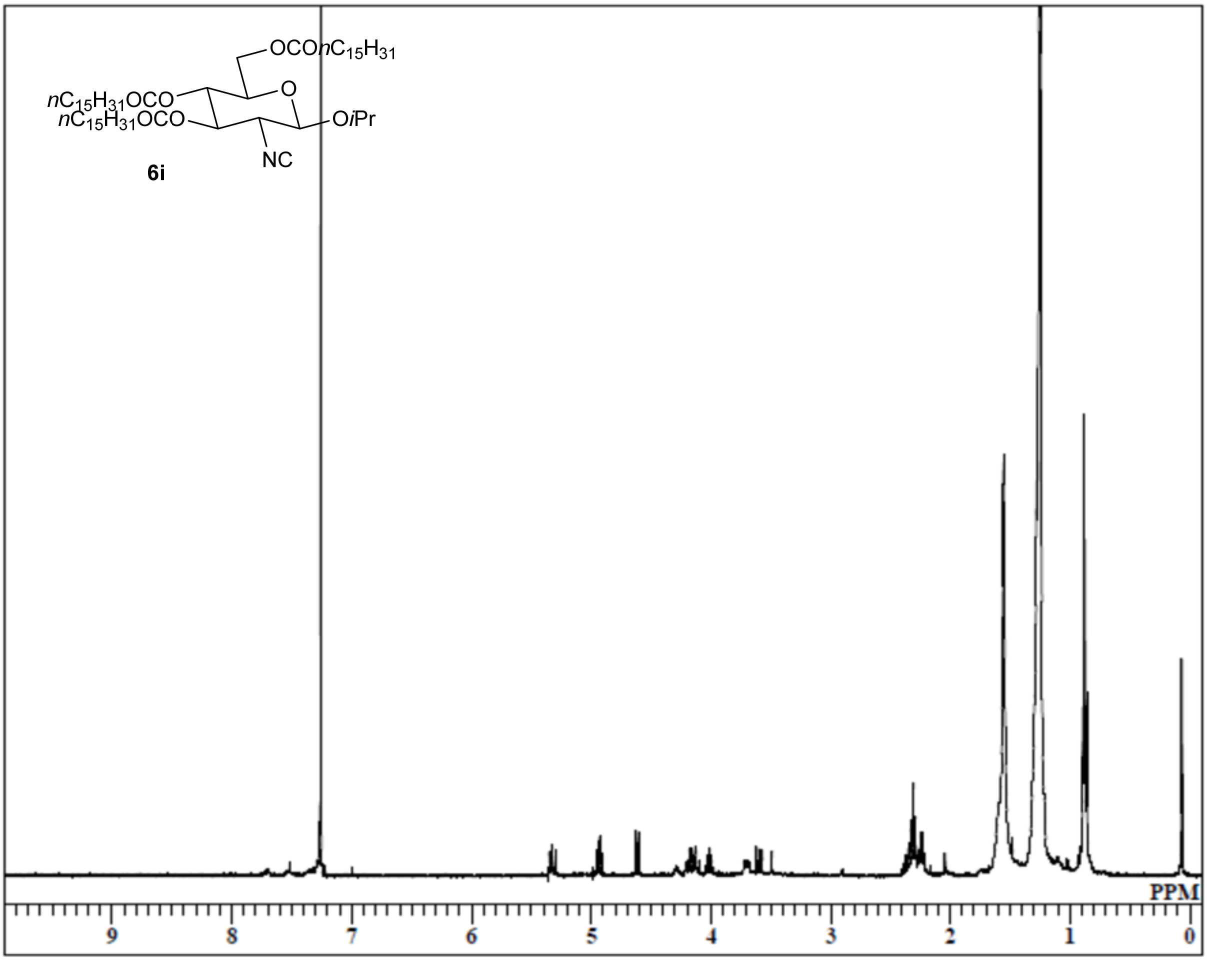
OCOnC15H31
nC15H31OCO
nC15H31OCO
O
OiPr
NC
6i
PPM
9
8
7
6
5
4
3
2
1
0

6i

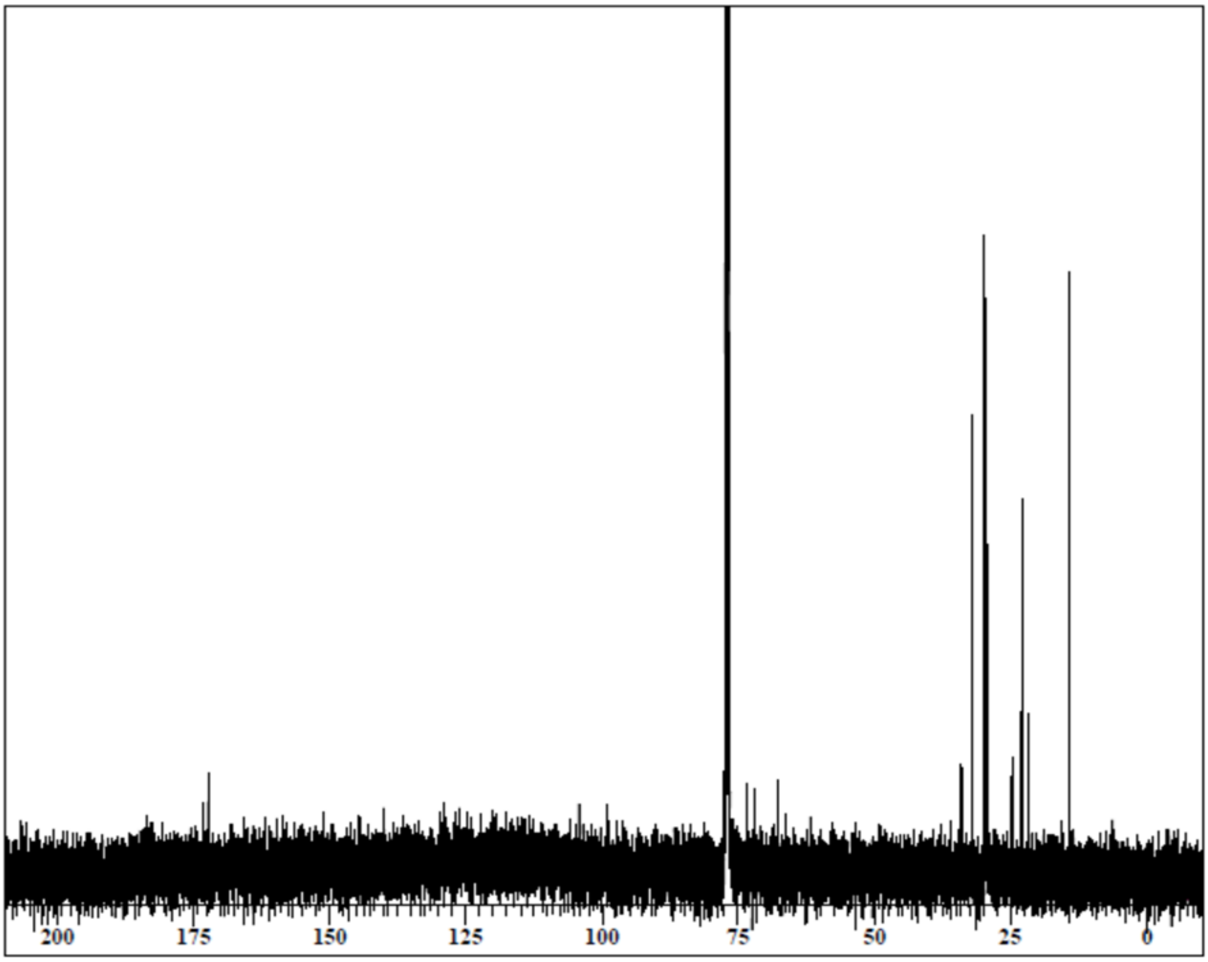
200
175
150
125
100
75
50
25
0

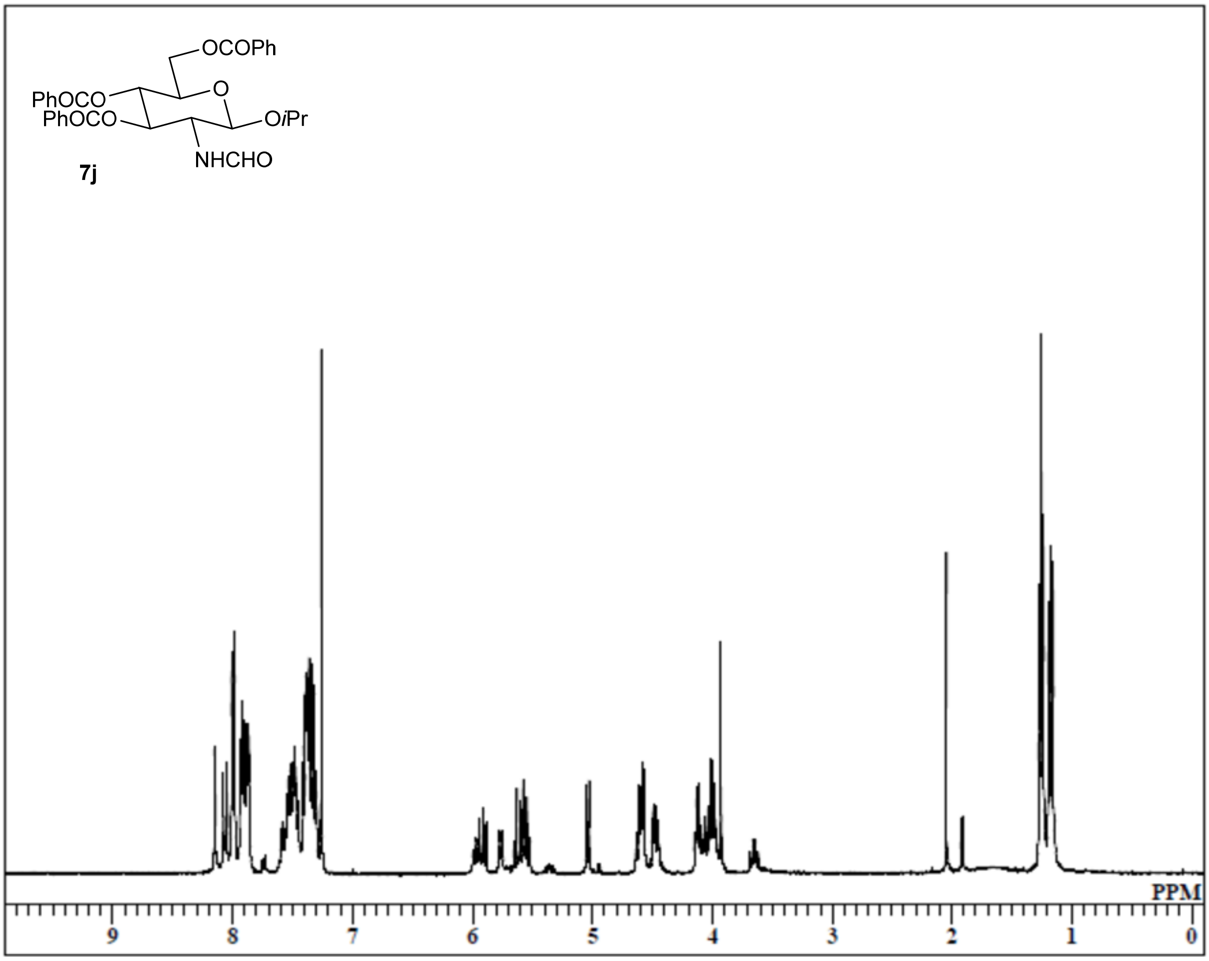
OCOPh
PhOCO
PhOCO
O
O*i*Pr
NHCHO
7j
PPM
9
8
7
6
5
4
3
2
1
0

7j

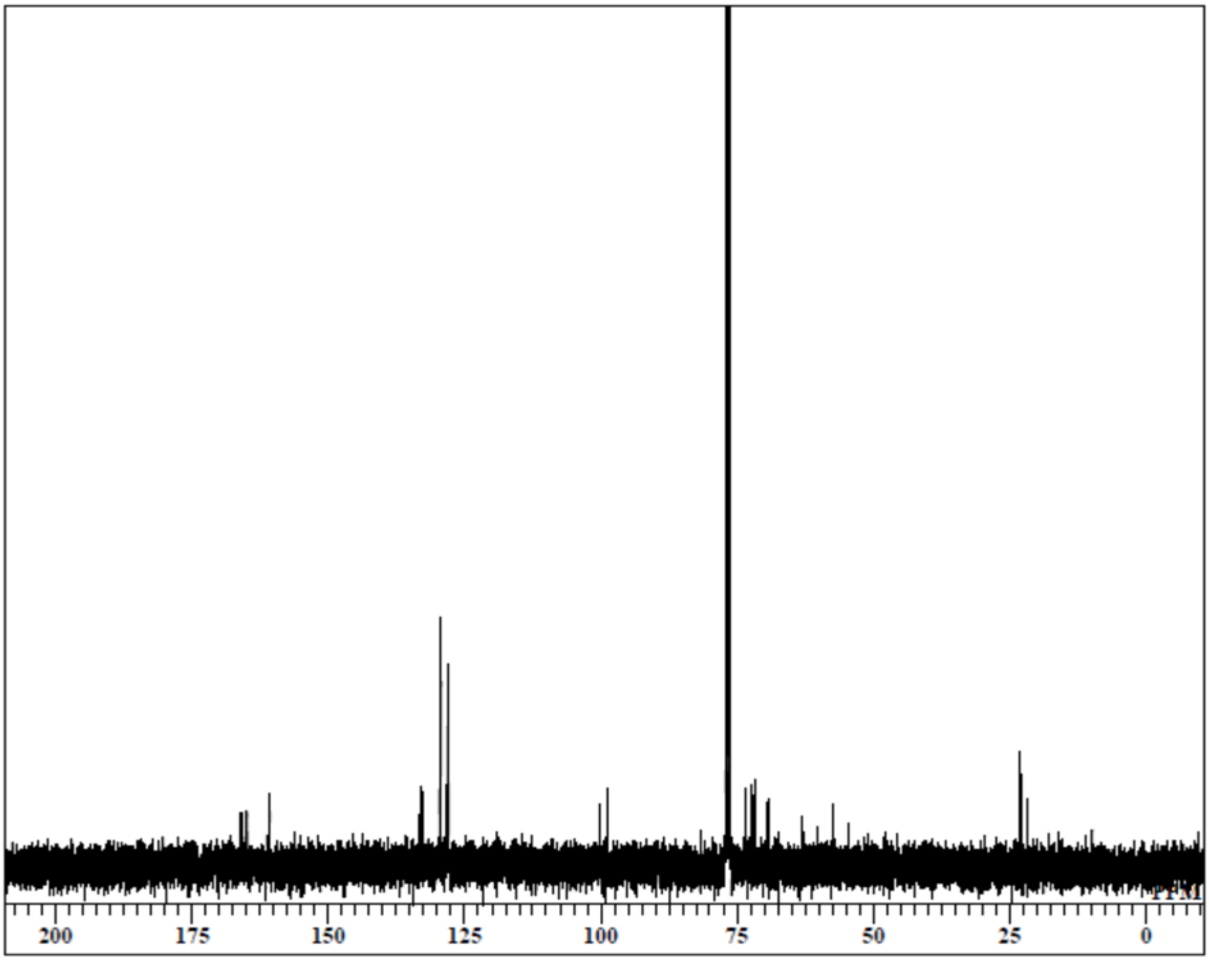
200
175
150
125
100
75
50
25
0

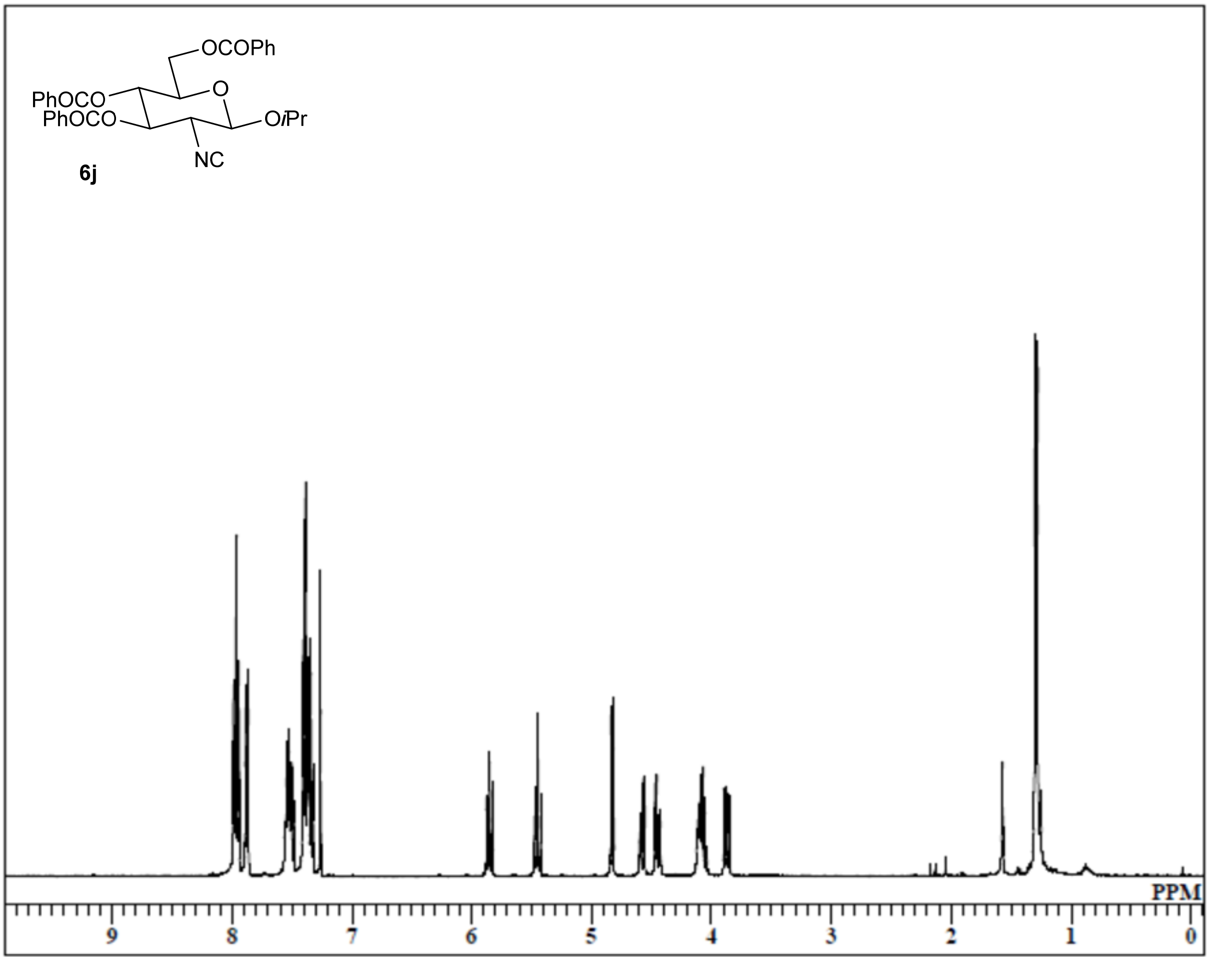
OCOPh
PhOCO
PhOCO
O
O*i*Pr
NC
6j
PPM
9 8 7 6 5 4 3 2 1 0

6j

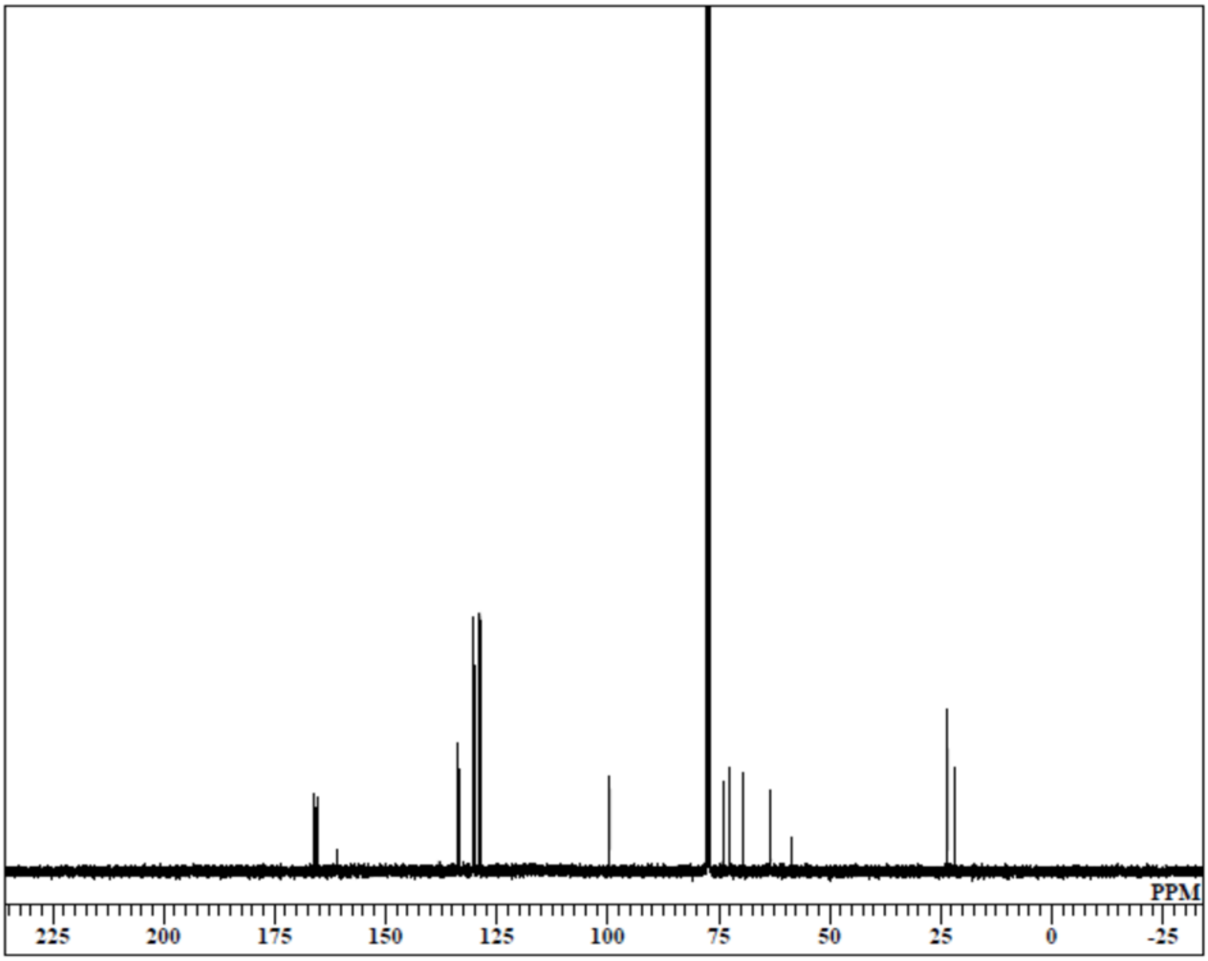
PPM
225 200 175 150 125 100 75 50 25 0 -25

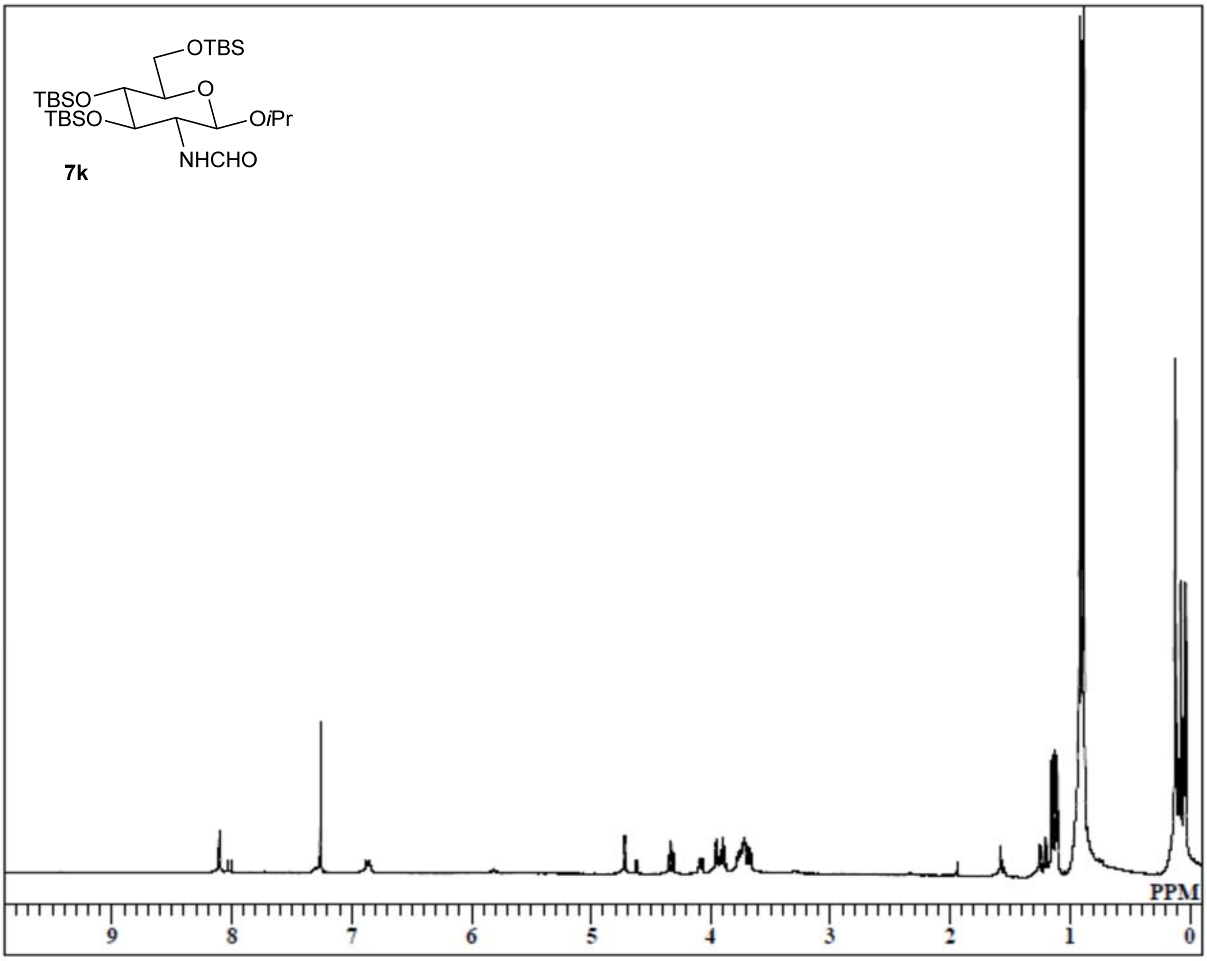
OTBS
TBSO
TBSO
O
OiPr
NHCHO
7k
PPM
9 8 7 6 5 4 3 2 1 0
7k

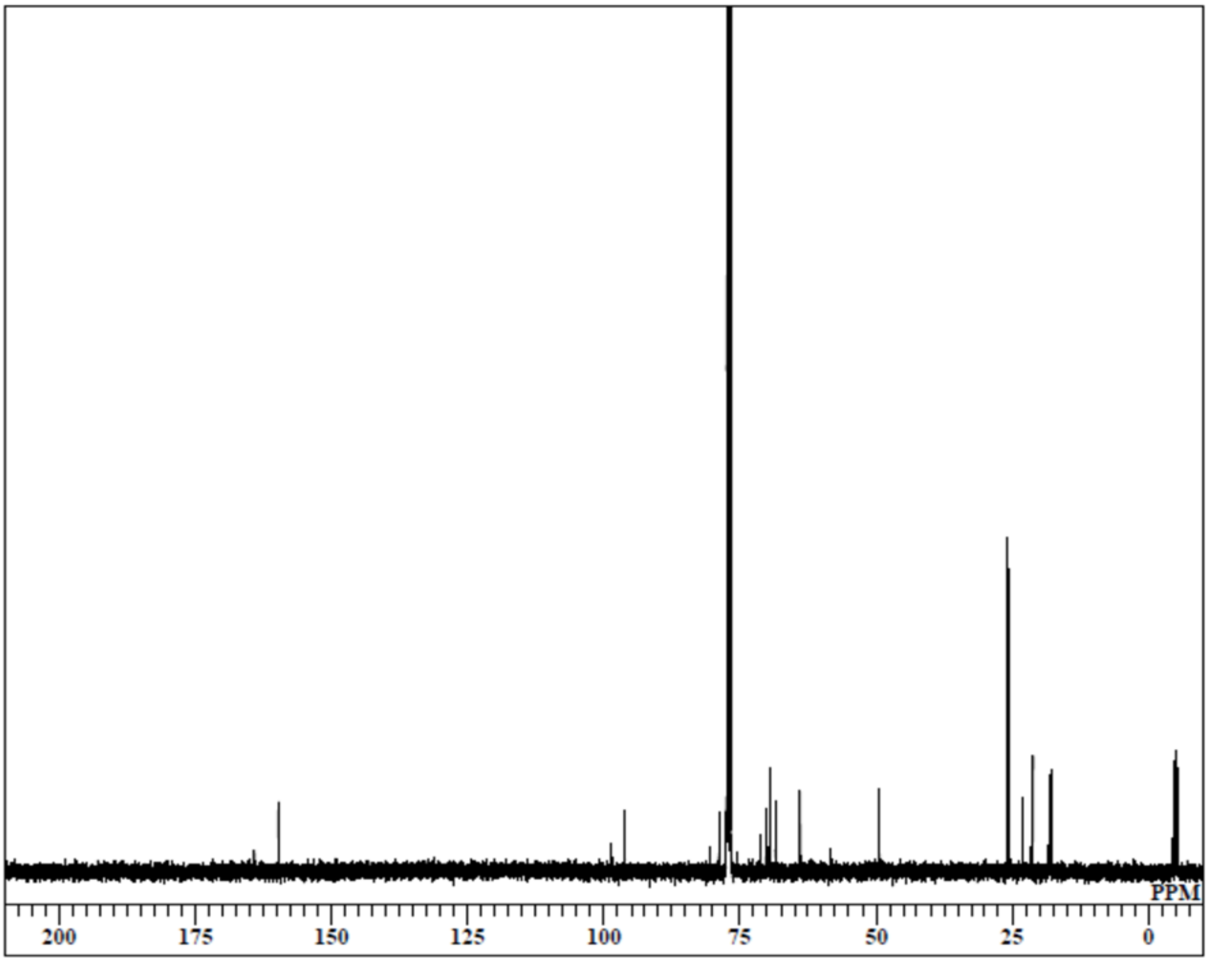
PPM
200 175 150 125 100 75 50 25 0

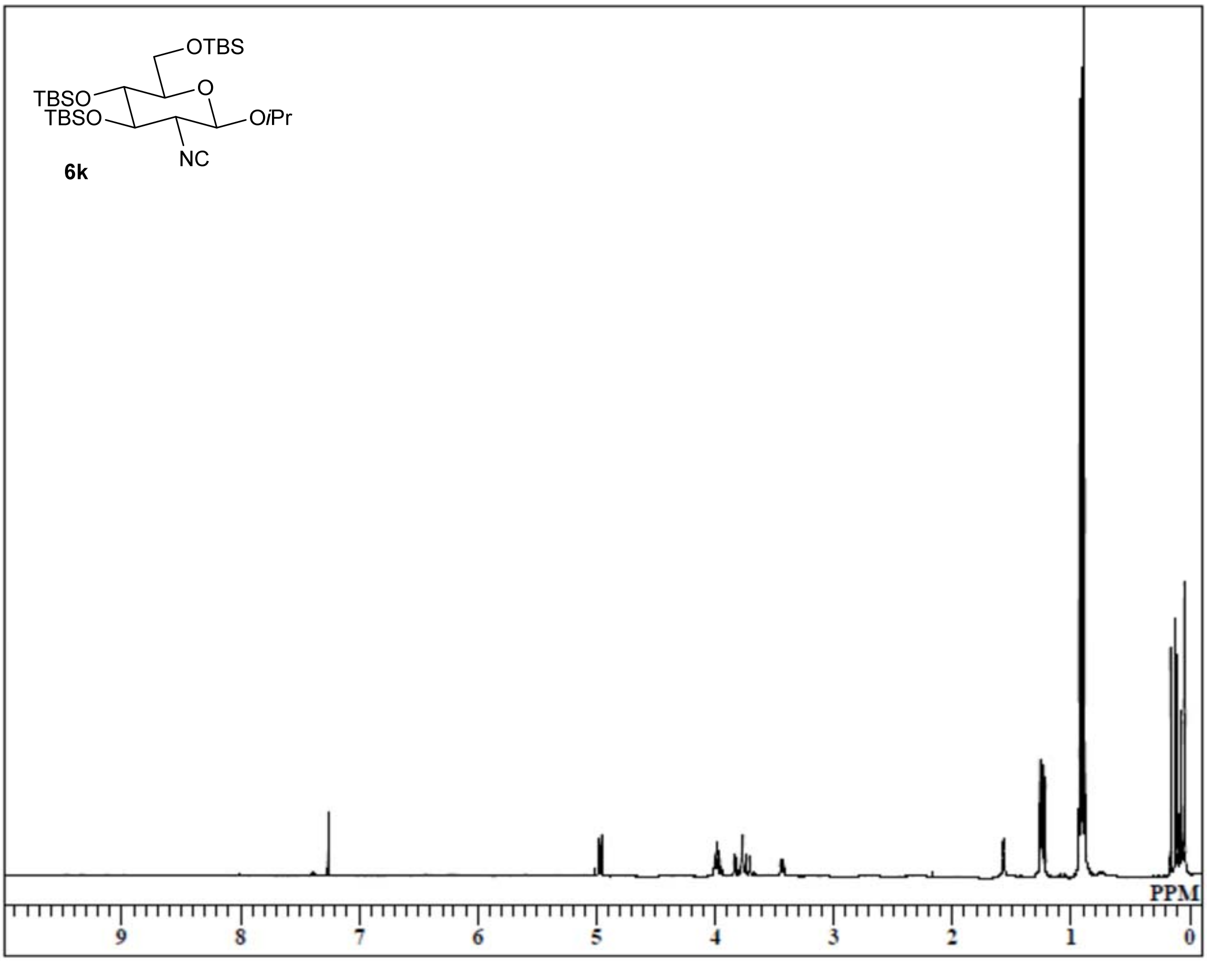
OTBS
TBSO
TBSO
O
O*i*Pr
NC
6k
PPM
9 8 7 6 5 4 3 2 1 0

6k

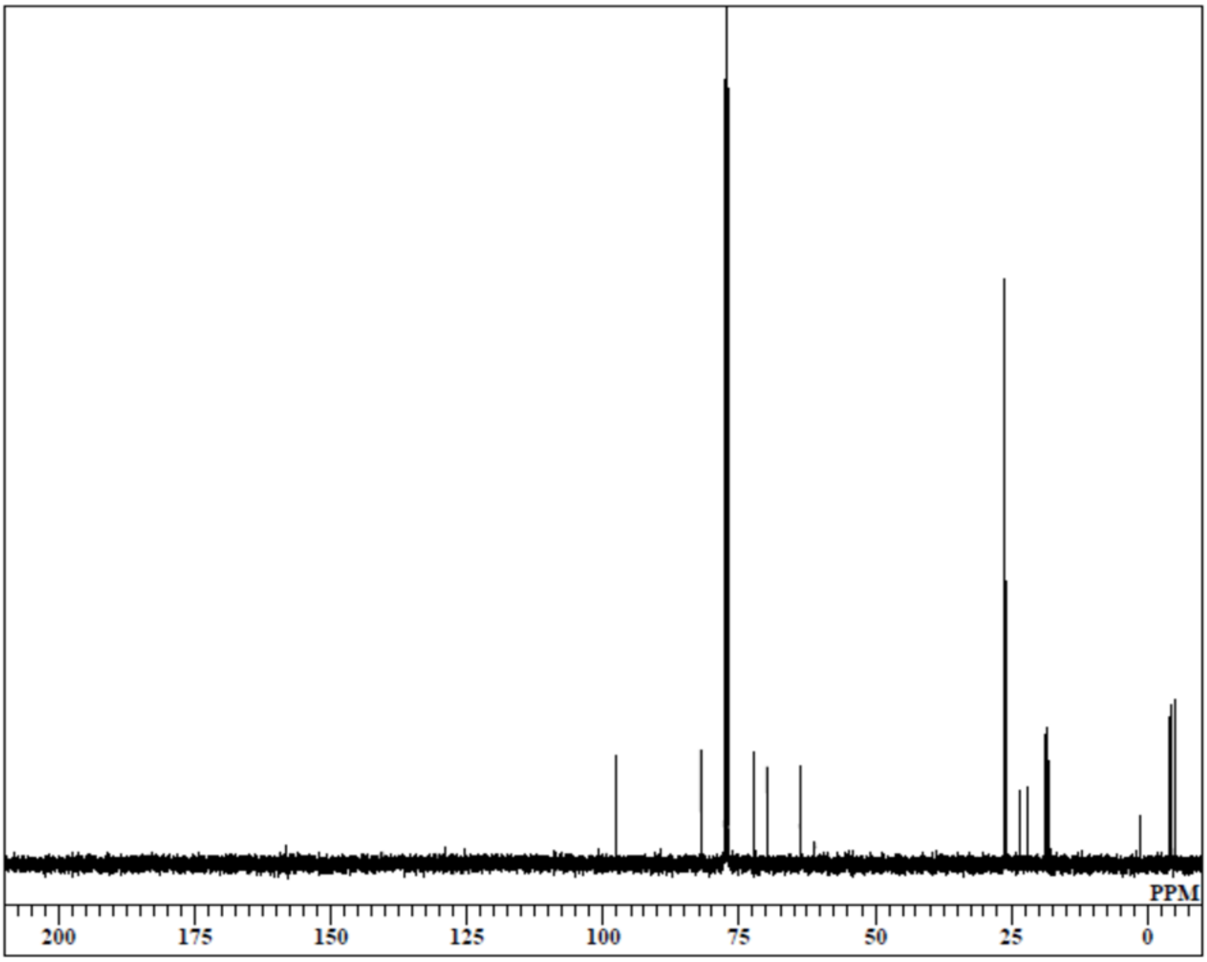
PPM
200 175 150 125 100 75 50 25 0
